# Unveiling Thermoelectric Properties of SURMOF Nanofilms: A New Frontier in Molecular Thermoelectrics

**DOI:** 10.1002/advs.202510730

**Published:** 2025-08-31

**Authors:** Jiwoo Park, Soo Jin Cho, Dong Su Lee, Sohyun Park

**Affiliations:** ^1^ School of Chemistry and Energy Sungshin Women's University Seoul 01133 Republic of Korea; ^2^ Institute of Advanced Composite Materials Korea Institute of Science and Technology (KIST) Wanju‐gun Jeonbuk 55324 Republic of Korea; ^3^ Center for NanoBio Applied Technology Sungshin Women's University Seoul 01133 Republic of Korea

**Keywords:** Molecular Thermoelectrics, Surface‐Mounted Metal‐Organic Framework (SURMOF), Self‐assembled monolayer (SAM), Power Factor, Electronic Structure Modulation

## Abstract

Molecular thermoelectric materials, which harness molecular‐level design principles to optimize energy conversion, have emerged as a promising strategy for addressing the limitations of bulk inorganic thermoelectrics, such as brittleness and high production costs. In this study, a layer‐by‐layer (LbL) engineered HKUST‐1 surface‐mounted metal‐organic framework (SURMOF) nanofilm is proposed as a promising thermoelectric nanostructure, systematically characterized across its thickness. By employing LbL growth of HKUST‐1 on self‐assembled monolayers (SC*_n_* COOH, *n* = 2, 10), nanofilms ranging from 5 to 30 nm in thickness are successfully fabricated. Thermoelectric characterization of these nanofilms revealed a significant enhancement in Seebeck coefficient (*S*) and power factor (PF), with PF values surpassing those of conventional organic SAMs by a factor of 10^3^. Ultraviolet photoelectron spectroscopy (UPS) measurements further confirmed a correlation between molecular orbital alignment and thermoelectric performance, particularly in junctions doped with guest molecules such as ferrocene (Fc) and 7,7,8,8‐tetracyanoquinodimethane (TCNQ). These findings establish SURMOF nanofilms as a viable molecular thermoelectric architecture, offering enhanced carrier transport, guest‐responsive electronic properties, and precise structural control at the nanoscale.

## Graphical abstract

Layer‐by‐layer engineered surface‐mounted metal‐organic framework (SURMOF) nanofilms exhibit enhanced thermoelectric performance through precise control of thickness, molecular alignment, and guest doping.

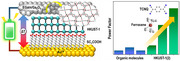

## 1 Introduction

As global warming intensifies, the accumulation of excess heat on Earth—now referred to as “global boiling”—underscores an urgent need for advanced thermoelectric materials capable of converting waste heat into electricity. Currently, bulk inorganic thermoelectric modules have shown high thermoelectric performance (ZT ≈1) ^[^ ^1^, ^2^, ^3^, ^4^, ^5^, ^6^, ^7^, ^8^, ^9^, ^10^, ^11^ ^]^; however, their intrinsic brittleness and high production costs continue to pose challenges in processing and practical applications.^[^ ^12^, ^13^, ^14^, ^15^ ^]^ Furthermore, the complexity of molecular packing within bulk structures and the limited understanding of interface properties and underlying mechanisms suggest that these materials are approaching their performance limits, complicating the optimization of key parameters for further efficiency improvements.^[^ ^13^, ^16^, ^17^, ^18^ ^]^ To overcome these challenges, a molecular‐level approach to thermoelectric materials is essential, providing atomic‐scale design principles, clarifying performance mechanisms, and enabling the prediction of high‐efficiency structures, while also highlighting the potential of individual molecules as effective thermoelectric materials.^[^ ^19^, ^20^, ^21^, ^22^, ^23^, ^24^ ^]^ Organic molecules, long a focus of research on molecular thermoelectrics, are valued for their accessibility, ease of fabrication, and well‐characterized interfaces.^[^ ^19^, ^25^, ^26^, ^27^, ^28^, ^29^, ^30^, ^31^, ^32^, ^33^, ^34^, ^35^ ^]^ Despite these advantages, their inherently low Seebeck coefficient and thermoelectric performance restrict their practical applications.

**Figure 1:**
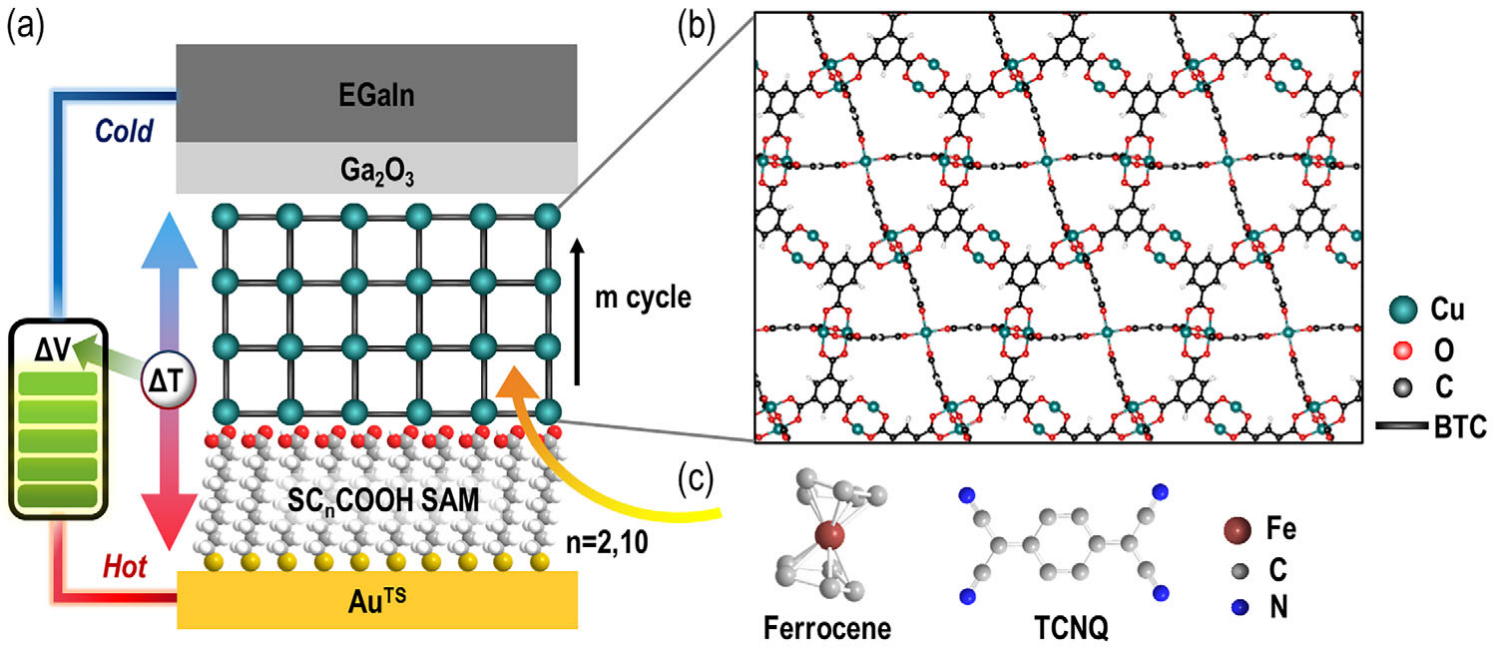
(a) Schematic illustration of the SURMOF structure and Au^TS^/SC*_n_* COOH/HKUST‐1//Ga_2_O_3_/EGaIn thermoelectric junctions. In this notation, “/” denotes an ionic or covalent interface, while “//” indicates a van der Waals interface. (b) Crystalline structure of HKUST‐1 layers. (c) Molecular structures of guest molecules used: Fc and TCNQ.

In this study, we present a molecular mixing strategy designed to enhance thermoelectric performance by integrating organic molecules with metal components in an orderly manner at the molecular level. The inclusion of metal components is pivotal, as they play essential roles in establishing electrical contact, facilitating charge injection, aligning energy levels, and enabling carrier transport within molecular junctions.^[^ ^23^, ^36^, ^37^, ^38^, ^39^, ^40^, ^41^, ^42^, ^43^ ^]^ Notably, we report for the first time the thermoelectric properties of surface‐mounted metal‐organic framework (SURMOF) nanofilms, with thicknesses ranging from 5 to 30 nm. These nanofilms exhibit a substantial increase in power factor (PF)—≈1700‐fold—compared to conventional organic self‐assembled monolayers (SAMs). To fabricate the nanofilms, HKUST‐1 MOF layers—composed of paddle‐wheel Cu(II) clusters coordinated with benzene‐1,3,5‐tricarboxylic acid (BTC)—were grown via a layer‐by‐layer (LbL) method on *n*‐mercaptoalkanoic acid (SC*_n_* COOH, *n* = 2, 10) SAMs, deposited onto ultra‐flat template‐stripped gold (Au^TS^) substrates (Figure 1a,b). The SURMOF nanofilms were electrically and thermoelectrically characterized using a liquid metal top electrode comprising eutectic Ga–In alloy (EGaIn) coated with a conductive native oxide layer. This liquid‐electrode‐based large‐area junction technique minimizes damage to nanofilms while enabling the reproducible formation of 15 – 40 molecular junctions across multiple samples, ensuring highly reliable measurements.

The Seebeck coefficient (*S*) is a critical parameter for evaluating thermoelectric performance, defined as the voltage difference generated across a material in response to a temperature gradient (*S* = –Δ*V*/Δ*T*). At the molecular junction level, *S* can be described using the Mott formula, which is expressed in terms of the Lorentzian transmission function, *T*(*E*) as shown in Equations (1) and (2).^[^ ^44^, ^45^, ^46^, ^47^ ^]^

(1) $$S\approx {\left.\frac{\pi^{2}{k_{B}}^{2}T}{3e}\frac{\partial \ln T\left(E\right)}{\partial E}\right|}_{E=E_{F}}$$

(2) $$T\left(E\right)\;=\sum_{i=1}^{2}\frac{\Gamma_{1}\Gamma_{2}}{{\left(E-\;E_{\mathrm{MO}}\right)}^{2}+{\left(\Gamma_{1}+\;\Gamma_{2}\right)}^{2}/4}$$

Here, *k_B_* is the Boltzmann constant, *T* is the average temperature of the junction, *e* is the electron charge, *E*_F_ is the Fermi energy, Γ_*i*_(*i* = 1, 2) represents the coupling strength between molecular energy levels and electrode energy states, and *E*_MO_ denotes the energy level of the accessible molecular orbital (typically, the HOMO or LUMO). Based on the model, the energy topography across a molecular junction and *S* values are closely correlated: the slope of *T*(*E*) of *E*_MO_ near *E*_F_ strongly affects *S*, with steeper slopes resulting in higher *S* values.

To explore strategies for enhancing the thermoelectric performance of SURMOF nanofilms, particularly the Seebeck coefficient (*S*) and power factor (PF) values, we adopted structural engineering approaches aimed at tailoring the electronic structure of the SURMOF. These modifications involved (1) varying the SAM chain lengths, (2) optimizing the number of MOF fabrication cycles, and (3) introducing guest molecules, such as ferrocene (Fc) and 7,7,8,8‐tetracyanoquinodimethane (TCNQ), into the MOF pores (**Figure** **1**c). Ultraviolet photoelectron spectroscopy (UPS) measurements confirmed that the improved electrical conductivity and thermopower of both doped and undoped SURMOFs—compared to conventional organic SAMs—originated from a reduced energy offset (Δ*E* = *E*_MO_ – *E*_F_) of ≈0.2 eV between the Fermi level and the HOMO energy level.

## 2 Result and Discussion

### 2.1 Preparation and Characterization of SURMOF Nanofilms

HKUST‐1 layers were synthesized on SC*_n_* COOH (*n* = 2, 10) SAMs, which were uniformly fabricated ultra‐flat Au^TS^ substrates using a step‐by‐step dipping process.^[^ ^48^ ^]^ This method involved alternating immersions in solutions of benzene‐1,3,5‐tricarboxylic acid (BTC) and copper acetate, with dipping cycles ranging from 1 to 7 cycles. The exposed carboxylic acid (–COOH) groups on the SAMs coordinate with Cu^2^⁺ ions, forming surface‐bound metal sites that initiate the vertical growth of HKUST‐1 through subsequent BTC coordination. This interfacial bonding plays a key role in achieving uniform and oriented MOF nanolayers.^[^ ^49^ ^]^ SAMs of varying alkyl chain lengths were prepared by immersing Au^TS^ chips in ethanolic solutions of 3 mm 3‐mercaptopropionic acid or 11‐mercaptoundecanoic acid. Guest molecule loading was conducted by immersing dried SURMOF‐coated chips in saturated solutions of guest molecules (Fc or TCNQ) for 24 h at room temperature. These loading conditions were optimized to maximize and stabilize dopant incorporation into the SURMOF structure and confirmed by monitoring current density (*J*) variations at a given voltage. Detailed preparation procedures and doping optimization results are provided in the Supporting Information (Figures S1–S4, Supporting Information). For clarity, SURMOF structures are denoted as HKUST‐1(*n*), where “*n*” represents the number of carbon atoms in the alkyl chain backbone of the SAM. Guest molecule‐loaded structures are designated as Fc@HKUST‐1(*n*) or TCNQ@HKUST‐1(*n*).

**Figure 2:**
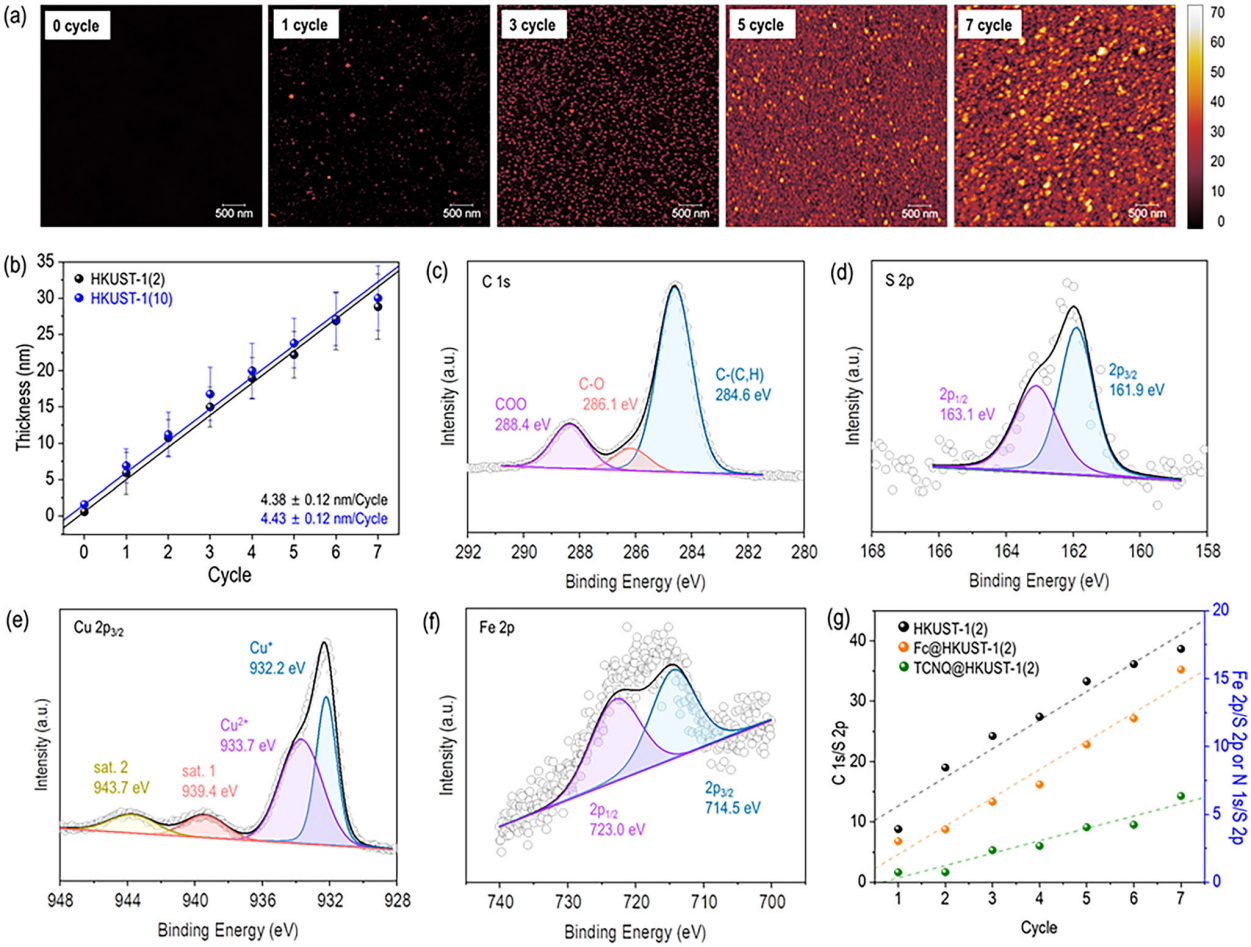
(a) AFM images of HKUST‐1 SURMOF growth on SC_2_COOH SAMs after 0, 1, 3, 5, and 7 cycles. (b) Average film thickness of HKUST‐1(*n*) (*n* = 2 or 10 versus growth cycles). (c‐f) High‐resolution XPS spectra of Fc@HKUST‐1(2) at 6 cycles for C 1s, S 2p, Cu 2p_3/2_, and Fe 2p. (g) Plots of peak integration ratios: C 1s/S 2p for HKUST‐1(2), Fe 2p/S 2p for Fc@HKUST‐1(2), and N 1s/S 2p for TCNQ@HKUST‐1(2) as a function of growth cycle.

The surface topography and thickness of SURMOF films were analyzed using atomic force microscopy (AFM) (**Figure** **2**a,b). As the number of growth cycles increased, the film thickness increased at an average rate of ≈4.4 nm per cycle, accompanied by an increase in surface coverage. The thickness of HKUST‐1(10) was slightly greater than that of HKUST‐1(2), attributed to the longer chain length of the SC_10_COOH SAM. This slight thickness difference likely arises from the higher packing density and vertical alignment of longer‐chain SC₁₀COOH SAMs, which provide a more extended surface for MOF growth.^[^ ^50^, ^51^ ^]^ The growth orientation of HKUST‐1(2) and (10) after 7 cycles was further confirmed through out‐of‐plane grazing incidence X‐ray diffraction (GI‐XRD), which indicated that HKUST‐1(10) exhibited a more preferred [100] orientation compared to HKUST‐1(2) (Figure S5, Supporting Information). This observation aligns with previous studies showing that HKUST‐1 grown on longer alkyl chains tends to exhibit enhanced [100] orientation.^[^ ^49^, ^50^ ^]^ Additional AFM images of HKUST‐1(2) and HKUST‐1(10) at all growth stages are provided in Figures S6, S7 (Supporting Information).

The chemical composition and epitaxial growth of SURMOFs were examined using X‐ray photoelectron spectroscopy (XPS) after each growth cycle. Representative high‐resolution XPS spectra of Fc@HKUST‐1(2) at 6 cycles were acquired for C 1s, S 2p, Cu 2p, and Fe 2p (Figure 2c–f). The C 1s spectrum was deconvoluted into three peaks at 284.6, 286.1, and 288.4 eV, corresponding to C–(C, H), C–O, COO bonds, respectively (Figure 2c). The C–(C, H) peak arises from the aromatic ring in BTC, while the C–O peak originates from the carboxylic groups in BTC.^[^ ^52^ ^]^ The S 2p spectrum was deconvoluted into two peaks at 161.9 and 163.1 eV, indicative of chemisorbed thiolate species (Figure 2d).^[^ ^35^ ^]^ The Cu 2p_3/2_ spectrum included four peaks at 932.2, 933.7, 939.4, and 943.7 eV, corresponding to Cu^+^, Cu^2+^, and two satellite peaks (Figure 2e).^[^ ^52^, ^53^ ^]^ The Fe 2p spectrum was deconvoluted into two peaks at 714.5 and 723.0 eV, associated with the 2p_3/2_, 2p_1/2_ states of Fc, respectively (Figure 2f).^[^ ^54^, ^55^, ^56^ ^]^

Introduction of TCNQ into SURMOF pores led to the detection of nitrogen (N), with its N 1s spectrum showing peaks at 398.5, 400.0, and 401.4 eV, corresponding to TCNQ^−1^/TCNQ^−2^, TCNQ⁰, and shake‐up states, respectively (Figure S22, Supporting Information).^[^ ^57^ ^]^ High‐resolution XPS spectra for other SURMOFs exhibited similar results and are detailed in Figures S8–S28 (Supporting Information). The ratios of peak integrations for C 1s and S 2p increased linearly with the number of HKUST‐1 growth cycles, confirming epitaxial growth (Figure 2g). Furthermore, the integration ratios for doping elements (Fe 2p for Fc and N 1s for TCNQ) relative to S 2p increased proportionally with the growth cycles, indicating a linear increase in doping concentration. Overall, these XPS results support the successful formation of the desired SURMOF structures on Au^TS^ substrates.

### 2.2 Electrical Properties of HKUST‐1 SURMOF Nanofilms

**Figure 3:**
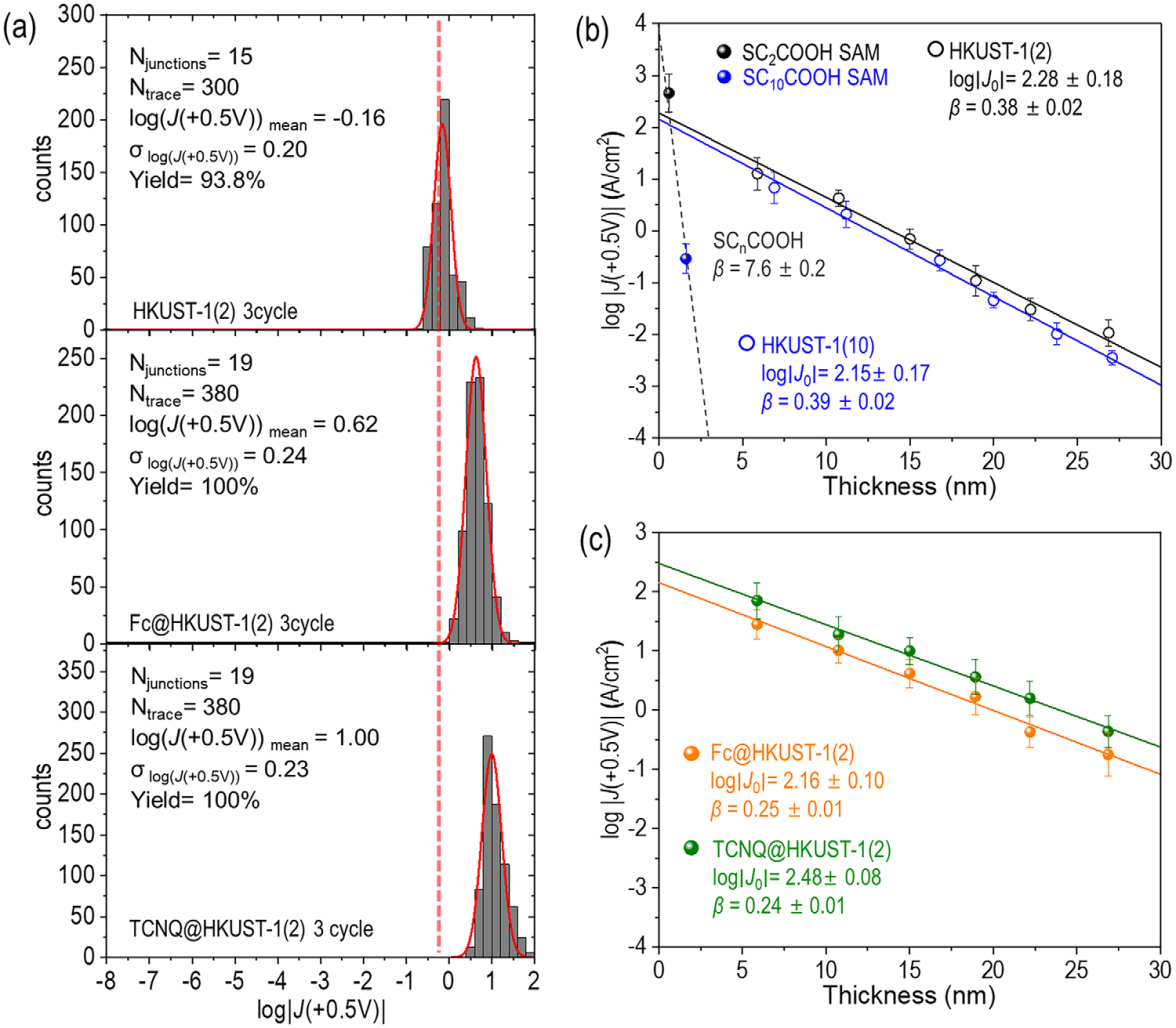
(a) Exemplary histograms of log|*J* (+0.5 V)| of HKUST‐1(2), Fc@HKUST‐1(2), and TCNQ@HKUST‐1(2) after 3 growth cycles. *N*_junction_ is the number of junctions measured, *N*_traces_ is the number of *J‐V* traces. (b) Plot of log (|*J*(+0.5 V)|) for HKUST‐1 (2) and (10) as a function of SURMOF thickness. Solid black circles represent SC_2_COOH, and solid blue circles represent SC_10_COOH SAM. Empty black circles correspond to HKUST‐1(2) and empty blue circles correspond to HKUST‐1(10). The dashed line indicates the reported length dependence of log (|*J*(+0.5 V)|) and associated *β* value for SC*_n_* COOH SAM.^[^ ^60^ ^]^ (c) Plot of the log (|*J*(+0.5 V)|) of guest@HKUST‐1(2) as a function of growth cycles.

The electrical properties of SURMOF films were investigated by measuring tunneling current density (*J*) as a function of applied voltage (*V*) ranging from +0.5 to –0.5 V using EGaIn‐based technique.^[^ ^58^, ^59^ ^]^ The EGaIn top electrode enables reversible and non‐invasive electric contacts with SURMOF films. The HKUST‐1//Ga_2_;O_3_ interface is assumed to be governed by van der Waals interactions and physisorption, due to the liquid nature of EGaIn and weak experimental adhesion. Similar non‐covalent interactions between carboxylic acid‐functionalized surfaces and Ga_2_O_3_ have also been reported previously.^[^ ^60^ ^]^ From 9 to 19 separate junctions for HKUST‐1(2) and (10), Fc@HKUST‐1(2), and TCNQ@HKUST‐1(2) SURMOF samples, we obtained 180–380 *J*–*V* traces (Figures S29–S32 and Table S1–S4, Supporting Information). The yield of working junctions ranged from 71 to 100%, demonstrating high reproducibility and reliability in SURMOF nanofilm fabrication and ensuring consistent electrical performance across multiple measurements. Values of *J* at +0.5 V across Au^TS^/HKUST‐1(*n*)//Ga_2_O_3_/EGaIn junctions exhibited log‐normal distributions (**Figure** **3**a). The mean (log|*J(*+0.5 V|_mean_) and standard deviation (*σ*_log|_ *_J_*_|_) were extracted by fitting of log|*J*| histograms with single Gaussian functions. Incorporation of TCNQ into HKUST‐1 pores led to a ≈1 order increase in *J*, with log*J*(+0.5 V, A·cm^−2^) reaching a log|*J*(+0.5 V)|_mean_ of 1.00, compared to the initial value of −0.16 for HKUST‐1(2) without guest molecules. Similarly, Fc loading increased log|*J*(+0.5 V)|_mean_ to 0.62. These results confirm that the integration of guest molecules into SURMOF nanostructures leads to an increase in hole density while maintaining the structural integrity and morphology of the frameworks.

Figure 3b,c shows that log|*J*| decreased linearly with the increasing HKUST‐1 layers. This linear regression means the tunneling current density across the junctions aligns closely with the behavior predicted by the simplified Simmons model.^[^ ^30^, ^61^, ^62^ ^]^

(3) $$J=J_{0}\times \exp\left(-\beta \times d\right)$$

where *J*_0_ (A·cm^−2^) is the charge injection current density, *β* (nm^−1^) is the tunneling decay constant, and *d* (nm) is the film thickness. From the slope and intercept of the log|*J*|_mean_ versus thickness plot at +0.5 V, the *β* values for pristine HKUST‐1(2) and HKUST‐1(10) were determined to be 0.38 ± 0.02 and 0.39  ± 0.02 nm^−1^, respectively (Figure 3b). These values are ≈20 times lower than those observed in organic SAMs (7.6 ± 0.3 nm^−1^ for SC*_n_*_,_ 7.6 ± 0.2 nm^−1^ for SC*_n_* COOH),^[^ ^30^, ^60^ ^]^ highlighting that as the length increases, the reduction in electrical conductivity is effectively minimized. The log (|*J*(+0.5 V)|) values of SC_2_COOH and SC_10_COOH SAMs (Figure 3b) were consistent with previously reported values (Figure S33 and Table S5, Supporting Information).^[^ ^60^ ^]^ As shown in Figure 3c, doping with guest molecules such as TCNQ and Fc further reduced *β* values compared to pristine HKUST‐1(2). After doping with TCNQ, *β* resulted in 0.24 ± 0.01 nm^−1^ while Fc doping resulted in a *β* value of 0.25 ± 0.01 nm^−1^.

### 2.3 Thermoelectric Properties of HKUST‐1 SURMOF Nanofilms

**Figure 4:**
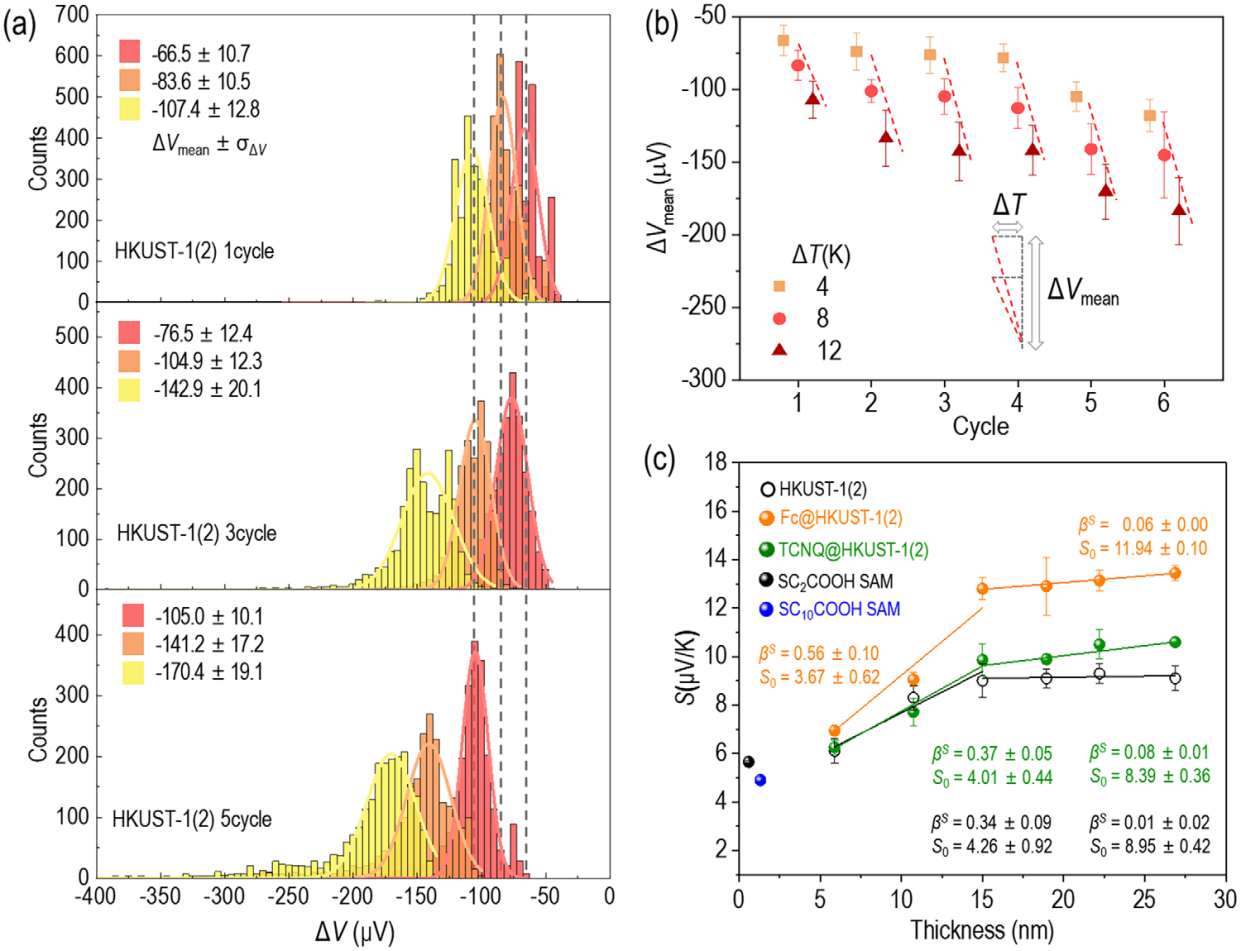
(a) Exemplary thermovoltage histograms of HKUST‐1(2) after 1, 3, and 5 growth cycles. (b) Trends of Δ*V* as a function of different Δ*T* for the HKUST‐1(2) SURMOFs. (c) Plot of *S* (µV·K^−1^) for HKUST‐1 (2) and (10), and guest@HKUST‐1(2) as a function of SURMOF thickness. Solid black circle represents SC_2_COOH, and solid blue circle represents SC_10_COOH SAM. Open black circles denote HKUST‐1(2), solid orange circles correspond Fc@HKUST‐1(2), and solid green circles correspond to TCNQ@HKUST‐1(2), respectively.

Employing an identical junction architecture, we systematically measured the Seebeck coefficient and power factor (PF) for each SURMOF nanofilm thickness. During the process, a SURMOF‐grown Au^TS^ chip was subjected to controlled heating. A microelectrode composed of EGaIn was subsequently positioned onto the film, facilitating the measurement of the thermoelectric voltage (Δ*V*) generated by the applied temperature differential (Δ*T*) across the junction. The Δ*T* value was determined by recording the temperatures of the bottom electrode (Au^TS^; hot side) and the top electrode (Ga_2_O_3_/EGaIn; cold side) using thermocouples, in accordance with previously reported methodologies (refer to section 4 and Supporting Information, Figure S34, Supporting Information).^[^ ^29^, ^32^, ^37^ ^]^ We measured Δ*V* values for Δ*T*  = 4, 8, and 12 K. **Figure** **4**a shows exemplary histograms of thermovoltage (Δ*V*, µV), from which mean thermovoltage (Δ*V*_mean_) and standard deviation (σ_Δ_ *_V_*) were extracted via single Gaussian fitting. The yield of working thermoelectric junctions ranged from 53 to 100%, demonstrating good device reproducibility. All SURMOF junctions exhibited positive Seebeck coefficients, wherein an increase in *ΔT* corresponded to a negative shift in *ΔV*. This trend suggests that hole transport is the dominant charge carrier mechanism, indicating HOMO‐governed charge transport rather than LUMO‐based conduction. A summary of the thermopower measurement data is provided in Table S6–S9 (Supporting Information). In addition, the overall distribution of Δ*V* broadened as Δ*T* increased and as the number of growth cycles progressed, as shown in the Δ*V* histograms (Figure 4b, Figure S35–S38, the Supporting Information). This trend was consistent with observations reported for other thermoelectric junctions.^[^ ^32^, ^35^, ^37^ ^]^ The increase in Δ*T* and the growing structural complexity induced greater degrees of freedom and intensified internal vibrational modes, leading to a wider dispersion in Δ*V* measurements.

By performing linear square fitting on the plot of Δ*V*_mean_ as a function of Δ*T*, we determined *S* values of entire junctions of SAMs and SURMOFs (Figure S34, Supporting Information). Figure 4c shows *S* values of SC*_n_* COOH SAMs, as well as pristine and doped HKUST‐1(2), plotted as a function of thickness. The *S* values of SC_2_COOH and SC_10_COOH were measured to be 5.5 and 4.9 µV·K^−1^(Figure S39 and Table S10, Supporting Information), respectively, exhibiting a decreasing trend consistent with alkanethiol (SC*_n_*) SAM systems (*n* = 2–10) cases.^[^ ^31^ ^]^ In contrast, HKUST‐1(2) exhibited an increasing trend in *S* values, ranging from 6.1 to 9.0 µV·K^−1^, as a function of SURMOF thickness up to 15 nm (cycle 3). This length dependence can be described using the following semi‐empirical parametric equation:

(4) $$S=S_{0}+d\times \beta^{s}$$

where *S*_0_ (µV·K^−1^) represents the *S* value at a hypothetical non‐shorting junction lacking an insulating organic component, while *β* ^S^ (µV·K^−1^·nm^−1^) denotes the slope of *S* as a function of film thickness (*d*, nm). The regression yielded *β* ^S^ and *S*_0_ values of 0.34 ± 0.09 µV·K^−1^·nm^−1^ and 4.26 ± 0.92 µV·K^−1^, respectively. Notably, at a thickness of 15 nm (cycle 3), a transition in *β* ^S^ was observed, decreasing from 0.34 to 0.01 µV·K^−1^·nm^−1^. Guest@HKUST‐1(2) exhibited an overall enhancement in the Seebeck coefficient (Figure 4c, orange and green). The *S* values for Fc@HKUST‐1(2) ranged from 6.9 to 13.5 µV·K^−1^, while those for TCNQ varied between 6.3 to 10.6 µV·K^−1^. Similar to pristine HKUST‐1(2), a transition in *β* ^S^ was observed at 15 nm (cycle 3), where it decreased from 0.56 to 0.06 µV·K^−1^·nm^−1^ for Fc@HKUST‐1(2) and from 0.37 to 0.08 µV·K^−1^·nm^−1^ for TCNQ@HKUST‐1(2). These length dependencies and transitions are attributed to variations in the electronic structure during SURMOF growth; further details are discussed in section 2.5.

### 2.4 Power Factor Calculation and Enhanced Thermoelectric Properties

**Figure 5:**
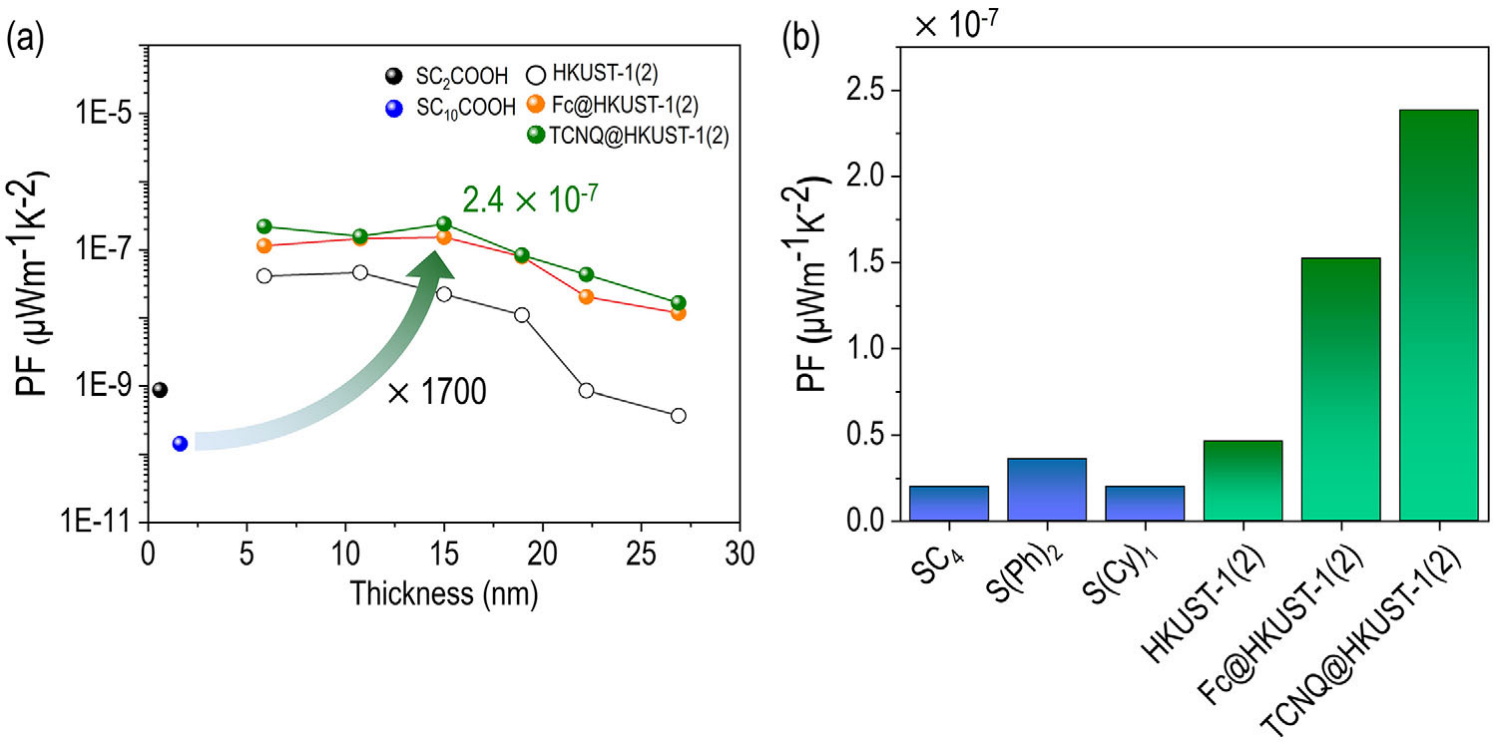
(a) Plot of PF of SC_n_COOH, HKUST‐1(2), and guest@HKUST‐1(2). (b) Comparison PF values of HKUST‐1 nanofilms and conventional organic based SAM (SC_4_ = butane‐1‐thiol, S(Ph)_2_ = biphenyl‐4‐thiol, S(Cy)_1_ = cyclohexane thiol) on Au^TS^ substrates.

The power factor (PF) is a key parameter that quantifies a material's ability to generate electricity at a given temperature. PF is defined as *σ* × *S* ^2^, where σ represents electrical conductivity and *S* denotes the Seebeck coefficient. To determine PF for nanometer‐scale films, tunneling conductivity (*σ*, µS·cm^−1^) was calculated from current density (*J*) in the ohmic region at +0.05 V, using the equation: *σ* =  *J* /*E* , where *E* is the applied electric field (V·m^−1^).^[^ ^30^ ^]^ **Figure** **5**a shows PF values of SC*_n_* COOH SAMs, as well as pristine and doped HKUST‐1(2), as a function of film thickness. Compared to pristine organic SAMs, the introduction of MOF and dopants resulted in an increase in PF, attributed to enhancements in both current density (*J*) and *S* values. For HKUST‐1(2), PF ranged from 4.6 × 10^−^^8^ to 3.7 × 10^−^^10^ µW·m^−1^·K^−^^2^, exceeding the values observed for SC_2_COOH (8.7 × 10^−^^10^ µW·m^−1^·K^−^^2^) and SC_10_COOH (1.4 × 10^−^^10^ µW·m^−1^·K^−^^2^). Upon guest molecule loading, PF values of Fc@HKUST‐1(2) and TCNQ@HKUST‐1(2), increased by ≈1 order of magnitude compared to their pristine counterparts, reaching 1.5 × 10^−7^ and 2.4 × 10^−7^ µW·m^−1^·K^−2^ at 3 cycles, respectively. Notably, the PF value of TCNQ@HKUST‐1(2) was 1700 times higher than that of organic SC_10_COOH SAM (Figure 5a), representing a significant enhancement relative to previously reported maximum PF values for nonconjugated and conjugated organic molecule SAMs (SC_4_ = butane‐1‐thiol, S(Ph)_2_ = biphenyl‐4‐thiol, S(Cy)_1_ = cyclohexane thiol) (Figure 5b).^[^ ^30^ ^]^ Additionally, while molecular SAMs typically achieve peak PF values only at ultrathin lengths (<1 nm) and experience a rapid decline as thickness increases, SURMOF structures show elevated PF values over 10 nm range, exhibiting a diminished length‐dependent decrease.^[^ ^30^ ^]^

### 2.5 Electronic Structure–Thermopower Relationship

**Figure 6:**
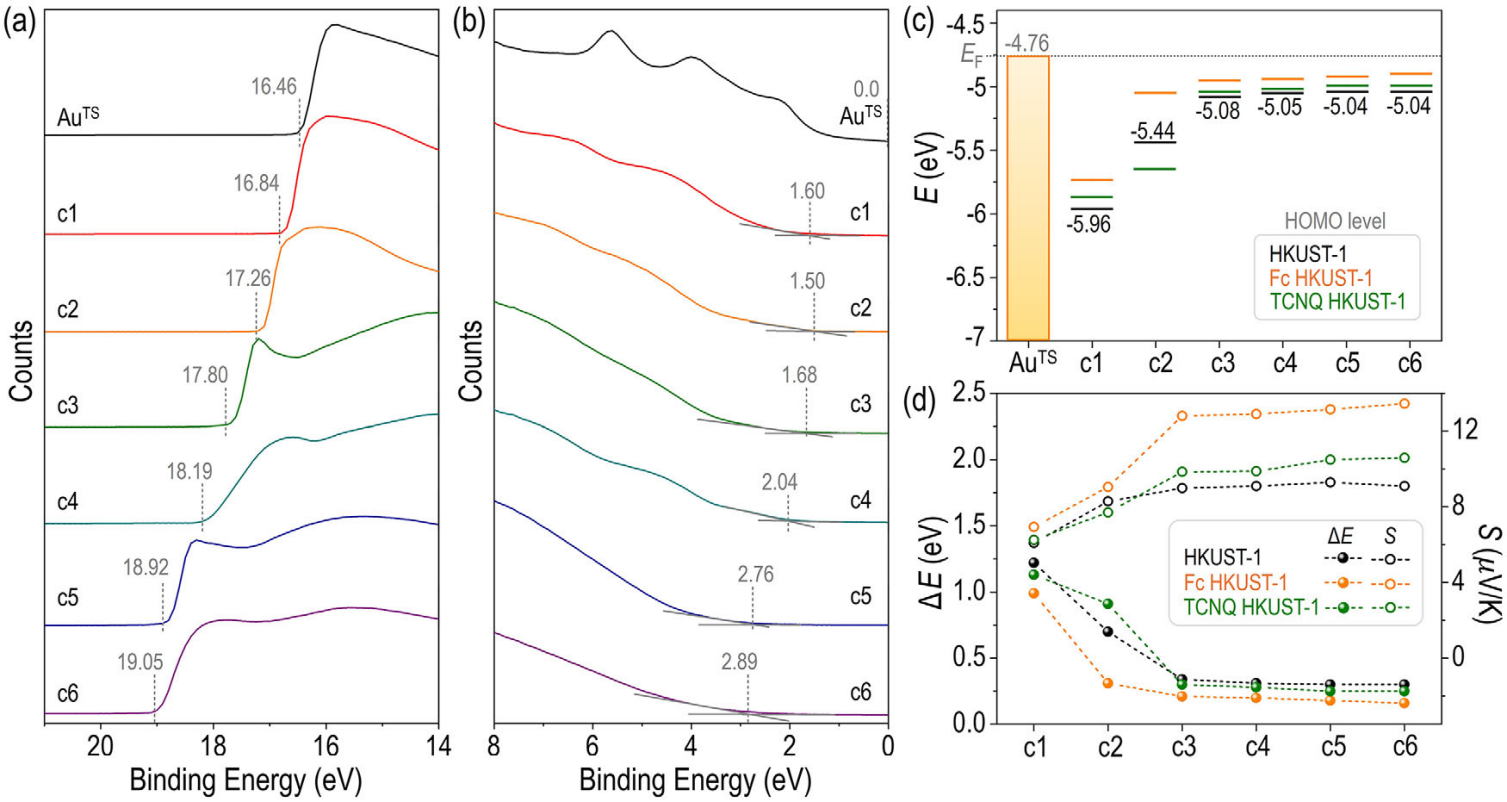
UPS spectra of HKUST‐1(2) (a) in the cut‐off region and (b) in the on‐set region. (c) Energy level diagram of doped and undoped HKUST‐1(2) relative to the work function of Au^TS^. (d) Plots of *S* and Δ*E* values of doped and undoped HKUST‐1(2) from 1 to 6 cycles.

To investigate the underlying causes of the observed trend and crossover point ≈15 nm in thermoelectric performances of SURMOF nanofilms, we experimentally determined the accessible orbital level —specifically, the HOMO levels— at each thickness relative to the Fermi energy (*E*_F_) using ultraviolet photoelectron spectroscopy (UPS). **Figure** **6**a,b shows the high‐binding‐energy cutoff (*E*_cutoff_) and onset (*E*_onset_) regions of the UPS spectra for HKUST‐1(2) from cycles 1 to 6 (UPS spectra of doped HKUST‐1 films are shown in Figures S40–S42, Supporting Information). The HOMO level was calculated using the equation: HOMO level = *hv –* (*E*_cutoff_ − *E*_onset_),^[^ ^63^, ^64^, ^65^ ^]^ where *hv* represents the irradiated photon energy (He(I) at 21.2 eV). As the number of growth cycles increased, the *E*_cutoff_ region progressively shifted toward higher energy, which is attributed to the formation of an interfacial dipole layer.^[^ ^63^, ^66^ ^]^ Notably, the relative HOMO energy of HKUST‐1(2) approaches the *E*_F_ of Au^TS^ (−4.76 eV) from cycle 1 to cycle 3, after which it remains constant (Figure 6c). A similar trend was observed for Fc@HKUST‐1(2) and TCNQ@HKUST‐1(2). Figure 6d presents the energy offset (Δ*E* = *E*_F_ − *E*_HOMO_) plotted against *S* values for various growth cycles. The Fc@HKUST‐1(2) exhibited consistently higher *S* values than both TCNQ‐doped and undoped HKUST‐1(2), along with the smallest energy offset. These results experimentally validate that a decrease in Δ*E* directly contributes to an increase in *S*, as described in Equations (1) and (2). Through UPS measurements, we have confirmed the underlying origin of thermoelectric performance trends in SURMOF structures and demonstrated the critical role of *ΔE* in determining both *S* and power factor (PF) values.

## 3 Conclusion

We have successfully demonstrated the thermoelectric performance of SURMOF nanofilms for the first time and their potential in molecular thermoelectric applications. We systematically optimized the Seebeck coefficient and power factor through precise structural engineering, achieving significant improvements over conventional organic SAM‐based junctions. However, since the observed Seebeck coefficient (≈10 µV·K^−1^) remains lower than those reported for other organic thermoelectric systems,^[^ ^24^, ^67^, ^68^ ^]^ further research is needed to enhance this parameter. Notably, doping with guest molecules such as ferrocene (Fc) and TCNQ further improved thermoelectric performance, confirming the critical role of orbital energy alignment in charge transport efficiency. UPS measurements validated the correlation between energy level alignment and Seebeck coefficient modulation, reinforcing the significance of controlling molecular electronic structure in thermoelectric junctions. These findings open new avenues for the design and integration of SURMOFs as advanced molecular thermoelectric materials, particularly by optimizing carrier mobility, orbital energy alignment, and long‐range charge transport characteristics.

## 4 Experimental Section

### Molecules and Materials

All reagents were used as received unless otherwise specified. Copper(II) acetate monohydrate, benzene‐1,3,5‐tricarboxylic acid (BTC), 7,7,8,8‐tetracyanoquinodimethane (TCNQ), ferrocene (Fc), 3‐mercaptopropionic acid, and 11‐mercaptoundecanoic acid were obtained from Tokyo Chemical Industry. Thiol‐based compounds were maintained under N_2_ atmosphere at temperatures below 4 °C to prevent oxidation and degradation. The eutectic gallium–indium (EGaIn; 99.99%) was purchased from Sigma–Aldrich. Gold layers with a thickness of 200 nm were deposited on silicon wafers (100 mm diameter, resistivity range: 1–10 Ω·cm, thickness: 525 ± 50 µm) via electron‐beam evaporation. Glass substrates for template stripping were acquired from Matsunami and precisely cut into 1 cm × 1 cm sections. Optical adhesive (Norland NOA81) was used as supplied.

### Sample Preparation—Template‐Stripped Gold (Au^TS^) Preparation

Ultraflat gold substrates were fabricated following a previously reported protocol.^[^ ^69^ ^]^ Glass substrates (1 cm × 1 cm) were bonded to silicon wafers coated with a 200 nm gold layer via optical adhesive (OA), which was cured overnight under 365 nm UV light exposure. The gold‐coated glass chips (designated as Au^TS^: glass/OA/gold chip) were then detached from the silicon wafer using a razor blade.

### Sample Preparation—SAM Preparation

SAMs were prepared following a previously established procedure.^[^ ^60^ ^]^ Template‐stripped gold substrates (Au^TS^) were immersed in a 3 mm ethanolic solution (99.9%, anhydrous) of mercaptoalkanoic acid and incubated for 18 h at room temperature in the dark. After incubation, SAM‐coated Au^TS^ chips were thoroughly rinsed with clean ethanol and dried under ambient conditions.

### Sample Preparation—SURMOF preparation and Loading Guest Molecules

SURMOFs were synthesized using the liquid phase epitaxy (LPE) method. SAM‐functionalized Au^TS^ chips were immersed in a 1 mm ethanolic solution of Cu(II) acetate monohydrate for 15 min, followed by thorough rinsing with ethanol and drying under N_2_ gas. Subsequently, the chips were submerged in a 0.1 mm ethanolic solution of BTC ligand for 30 min, rinsed with ethanol, and dried with N_2_ gas. This deposition cycle was repeated 1 to 7 times at 50 °C to achieve the desired film thickness. Following the cyclic deposition process, the guest molecules were introduced into the framework. The SURMOF samples were first dried at 60 °C under a vacuum of 0.026 MPa for 20 min to remove residual solvent from the pores. The dried samples were then immersed in guest molecule solution (0.1 mm Fc or 2 mm TCNQ with ethanol solvents) for 24 h at room temperature, followed by rinsing.^[^ ^70^, ^71^ ^]^

### Characterization

Atomic force microscopy (AFM) measurements were performed using a Park Systems NX10 system operating in tapping mode with NCHR probes (Park Systems; spring constant: 42 N·m^−1^, resonance frequency: 330 kHz). The free oscillation amplitude was ≈15 nm, and the set point amplitude during scanning was ≈10 nm. AFM images were acquired over a 5 µm × 5 µm area at a scan rate of 0.97 Hz for the initial substrate before MOF growth and at scan rates between 0.2 and 0.59 Hz after MOF growth. AFM imaging was conducted to analyze the step height of particles formed during different cycles of MOF growth.

Out‐of‐plane grazing incidence X‐ray diffraction (GI‐XRD) patterns of SURMOF nanofilms were recorded using a Rigaku SmartLab X‐ray diffractometer with an incident wavelength (λ) of 1.5416 Å. Diffractograms were acquired over a 2θ range of 5 ° to 18 °, with a step size of 0.05 °, a scan speed of 3 ° min^−1^, and an incidence angle of 0.2 °.

X‐ray photoelectron spectroscopy (XPS) measurements were conducted using a Thermo Scientific K‐Alpha system equipped with a monochromated Al Kα X‐ray source (1486.6 eV).

Ultraviolet photoelectron spectroscopy (UPS) measurements were performed using a Thermo Fisher Scientific Escalab 250 Xi system equipped with a He I (21.2 eV) discharge lamp. A gold film was employed as a reference for Fermi edge calibration. The analysis results and interpretations can be found in the Results and Discussion section and Supporting Information for further details.

### Electric Measurements and Analysis—Tip Formation

The formation of EGaIn conical tips followed a previously established procedure.^[^ ^72^ ^]^ In brief, a 10 µL gas‐tight syringe was loaded with EGaIn, and a droplet was carefully extruded from the syringe needle. This hanging droplet was then brought into contact with an adhesive surface (e.g., bare Au), allowing the EGaIn to adhere. Using a micromanipulator, the needle was gently withdrawn, resulting in the formation of a conical tip upon detachment from the bulk EGaIn. To ensure consistency and prevent contamination, a new EGaIn conical tip was fabricated for each tunneling junction. In cases where whisker formation was observed during tip preparation, the affected tips were discarded, and new ones were generated.

### Electric Measurements and Analysis—Junction formation

Junction fabrication and subsequent measurements were conducted under ambient conditions, following established protocols.^[^ ^58^ ^]^ Electrical measurement of SURMOF junctions was performed using a Keithley 2450 Source Meter to measure tunneling current. The contact area diameter was determined at high magnification (≈×360), and assuming a circular geometry, the area was calculated to derive current density (*J*, A·cm^−^^2^). The presence of a SAM or a SURMOF was verified through an initial *J*– scan; if tunneling behavior was observed, 20 scans were recorded to ensure measurement reliability. Each trace followed a voltage sweep sequence of 0 V → +0.5 V → 0 V → ‐0.5 V → 0 V with a step size of 0.05 V, where a single trace represented two complete voltage scans. The yield of functional junctions was determined by comparing the total number of working junctions to the number of shorted ones. For histogram generation, the bin size was maintained at a fixed width of 0.2 for *J* values on a logarithmic scale, ensuring consistency in statistical analysis.

### Electric Measurements and Analysis—Determination of the Electric Field, Tunneling Conductivity, and Power Factor

Due to the use of ultraflat Au^TS^ as the bottom electrode, the electric field in a SAM or SURMOF‐based molecular junction was assumed to be uniform at all points. The electric field strength (*E*) can be approximated by maintaining a constant voltage (electric potential difference) between the gold bottom electrode and the EGaIn top electrode. Under the assumption of an infinite surface, *E* is defined as *E* = *V* /*d* (GV·m^−1^) where *V* represents the applied voltage between the electrodes, and *d* is the electrode separation distance. The thickness of the SURMOF nanofilms was used to estimate *d* and subsequently determine the electric field value.

Electrical conductivity (*σ*) was calculated using a previously established method.^[^ ^30^ ^]^ A *J* value measured at +0.05 V was used to determine the electric filed (*E*, V·m^−1^). Since electrical conductivity (*σ*) is the inverse of resistivity (*ρ*), it is defined as: *σ* = 1/*ρ* (µS·cm^−1^). In general, resistivity at a given point was defined as the ratio of the electric field to the density of the current induced at that point: *ρ* = *E*/*J* (cm·µS^−1^) where ρ is the resistivity of the conductive material, *J* (A·cm^−2^) is the current density, and *E* (GV·m^−1^) is the electric field strength. Since conductivity is the reciprocal of resistivity, it can also be expressed as: *σ* = 1/*ρ* = *J*/*E* (µS·cm^−1^).

The power factor (PF) was calculated using the determined *σ* values and the measured Seebeck coefficient (*S*) via the following relation: PF = *σ* *S* ^2^ (µW·m^−1^·K^−^^2^) where *S* is expressed in microvolts per kelvin (µV·K^−1^), and the resultant unit of PF is microwatts per meter per square kelvin (µW·m^−1^·K^−^^2^).

### Thermoelectric Measurements and Analysis

Thermoelectric measurement of SURMOF junctions was performed using a Keithley 2182A nanovoltmeter. The Seebeck coefficient (*S*, µV·K^−1^) of HKUST‐1(*n*) or SAM was determined based on the method outlined in.^[^ ^29^ ^]^ The *S* value was extracted using an equivalent circuit model for Au^TS^/HKUST‐1(*n*) or SAM//Ga_2_O_3_/EGaIn junctions, as illustrated in Figure S34 (Supporting Information). The *S*_HKUST‐1(_ *_n_*_) or SAM_ was calculated using the relation: Δ*V* = − (*S*_HKUST‐1(_ *_n_*_)_ −  *S*_W tip_) Δ*T* where *S*_W tip_ = 1 µV·K^−1^.^[^ ^73^ ^]^

## Conflict of Interest

The authors declare no conflict of interest.

## Supporting information



Supporting Information

## Data Availability

The data that support the findings of this study are available from the corresponding author upon reasonable request.

## References

[1] S. Yamanaka , A. Kosuga , K. Kurosaki , Alloys Compd. 352, 2003, 275.

[2] I. T. Witting , T. C. Chasapis , F. Ricci , M. Peters , N. A. Heinz , G. Hautier , G. J. Snyder , Adv. Electron. Mater. 2019, 5, 1800904.

[3] K. Imasato , S. D. Kang , G. J. Snyder , Energy Environ. Sci. 2019, 12, 965.

[4] J. Mao , H. Zhu , Z. Ding , Z. Liu , G. A. Gamage , G. Chen , Z. Ren , Science 2019, 365, 495. 31320557 10.1126/science.aax7792

[5] R. Shu , Y. Zhou , Q. Wang , Z. Han , Y. Zhu , Y. Liu , Y. Chen , M. Gu , W. Xu , Y. Wang , Adv. Funct. Mater. 2019, 29, 1807235.

[6] L. Hu , H. Wu , T. Zhu , C. Fu , J. He , P. Ying , X. Zhao , Adv. Eng. Mater. 2015, 5, 1500411.

[7] B. Poudel , Q. Hao , Y. Ma , Y. Lan , A. Minnich , B. Yu , X. Yan , D. Wang , A. Muto , D. Vashaee , Science 2008, 320, 634. 18356488 10.1126/science.1156446

[8] L.‐D. Zhao , S.‐H. Lo , Y. Zhang , H. Sun , G. Tan , C. Uher , C. Wolverton , V. P. Dravid , M. G. Kanatzidis , Nature 2014, 508, 373. 24740068 10.1038/nature13184

[9] C. Asker , C. Pipitone , F. Ursi , K. Chen , A. G. Ricciardulli , E. S. S. Galindez , S. Luong , P. Samorì , M. Reece , A. Martorana , J. Mater. Chem. A 2025, 13, 26009.

[10] T. Ghellab , H. Baaziz , Z. Charifi , Comput. Condens. Matter. 2025, 44, 01083.

[11] Y. Ding , L. Chen , Q. Zhang , R. Li , R. Li , L. Fan , X. Tan , J. Wu , G.‐Q. Liu , J. Jiang , Mater. Today Phys. 2025, 52, 101697.

[12] G. Li , Q. An , W. Li , W. A. Goddard III , P. Zhai , Q. Zhang , G. J. Snyder , Mater. 27, 2015, 6329.

[13] B. Russ , A. Glaudell , J. J. Urban , M. L. Chabinyc , R. A. Segalman , Nat. Rev. Mater. 2016, 1, 16050.

[14] H. Jin , J. Li , J. Iocozzia , X. Zeng , P. C. Wei , C. Yang , N. Li , Z. Liu , J. H. He , T. Zhu , Angew. Chem., Int. Ed. 2019, 58, 15206. 10.1002/anie.201901106 30785665

[15] Y. Du , S. Z. Shen , K. Cai , P. S. Casey , Prog. Polym. Sci. 2012, 37, 820.

[16] M. S. Dresselhaus , G. Chen , M. Y. Tang , R. Yang , H. Lee , D. Wang , Z. Ren , J. P. Fleurial , P. Gogna , Adv. Mater. 2007, 19, 1043.

[17] O. Bubnova , X. Crispin , Energy Environ. Sci. 2012, 5, 9345.

[18] C. Gayner , K. K. Kar , Prog. Mater. Sci. 2016, 83, 330.

[19] P. Reddy , S.‐Y. Jang , R. A. Segalman , A. Majumdar , Science 2007, 315, 1568. 17303718 10.1126/science.1137149

[20] K. Wang , E. Meyhofer , P. Reddy , Adv. Funct. Mater. 2020, 30, 1904534.

[21] A. Tan , S. Sadat , P. Reddy , Appl. Phys. Lett. 2010, 96, 013110.

[22] S. Park , H. Kang , H. J. Yoon , J. Mater. Chem. A 2019, 7, 14419.

[23] S. Mondal , A. Panda , T. N. Das , F. A. Rahimi , S. Kumar , P. Singh , V. Kaliginedi , T. K. Maji , J. Am. Chem. Soc. 2025, 147, 25201. 40403283 10.1021/jacs.5c00670

[24] M. Cohen Jungerman , S. Shmueli , P. Shekhter , Y. Selzer , Nano Lett. 2025, 25, 2756. 39918237 10.1021/acs.nanolett.4c05852 PMC11849033

[25] S. K. Yee , J. A. Malen , A. Majumdar , R. A. Segalman , Nano Lett. 2011, 11, 4089. 21882860 10.1021/nl2014839

[26] J. R. Widawsky , W. Chen , H. Vazquez , T. Kim , R. Breslow , M. S. Hybertsen , L. Venkataraman , Nano Lett. 2013, 13, 2889. 23682792 10.1021/nl4012276

[27] C. Evangeli , K. Gillemot , E. Leary , M. T. Gonzalez , G. Rubio‐Bollinger , C. J. Lambert , N. Agrait , Nano Lett. 2013, 13, 2141. 23544957 10.1021/nl400579g

[28] E. J. Dell , B. Capozzi , J. Xia , L. Venkataraman , L. M. Campos , Nat. Chem. 2015, 7, 209. 25698329 10.1038/nchem.2160

[29] S. Park , H. J. Yoon , Nano Lett. 2018, 18, 7715. 30418032 10.1021/acs.nanolett.8b03404

[30] S. Park , S. Kang , H. J. Yoon , ACS Cent. Sci. 2019, 5, 1975. 31893227 10.1021/acscentsci.9b01042 PMC6936095

[31] S. Park , N. Cho , H. J. Yoon , Chem. Mater. 2019, 31, 5973.

[32] S. Park , H. R. Kim , J. Kim , B. H. Hong , H. J. Yoon , Adv. Mater. 2021, 33, 2103177. 10.1002/adma.202103177 34453364

[33] S. Park , J. Jang , H. J. Yoon , J. Phys. Chem. C 2021, 125, 20035.

[34] S. Park , H. J. Yoon , ACS Appl. Mater. Interfaces 2021, 14, 22818. 34961308 10.1021/acsami.1c20840

[35] S. Park , J. W. Jo , J. Jang , T. Ohto , H. Tada , H. J. Yoon , Nano Lett. 2022, 22, 7682. 36067367 10.1021/acs.nanolett.2c03083

[36] J. Park , S. Park , ChemSusChem 2025, 18, 202402077. 10.1002/cssc.202402077 39582066

[37] S. Park , J. Jang , Y. Tanaka , H. J. Yoon , Nano Lett. 2022, 22, 9693. 36441166 10.1021/acs.nanolett.2c03974

[38] M. H. Garner , H. Li , Y. Chen , T. A. Su , Z. Shangguan , D. W. Paley , T. Liu , F. Ng , H. Li , S. Xiao , Nature 2018, 558, 415. 29875407 10.1038/s41586-018-0197-9

[39] A. K. Ismael , L. Rincón‐García , C. Evangeli , P. Dallas , T. Alotaibi , A. A. Al‐Jobory , G. Rubio‐Bollinger , K. Porfyrakis , N. Agraït , C. J. Lambert , Nanoscale Horiz. 2022, 7, 616. 35439804 10.1039/d1nh00527h

[40] L. Pan , Z. Ji , X. Yi , X. Zhu , X. Chen , J. Shang , G. Liu , R. W. Li , Adv. Funct. Mater. 2015, 25, 2677.

[41] L. G. Albano , T. P. Vello , D. H. de Camargo , R. M. da Silva , A. C. Padilha , A. Fazzio , C. C. Bufon , Nano Lett. 2020, 20, 1080. 31917590 10.1021/acs.nanolett.9b04355

[42] A. Chandresh , X. Liu , C. Wöll , L. Heinke , Adv. Sci. 2021, 8, 2001884. 10.1002/advs.202001884 PMC8024988 33854871

[43] J. Park , E. Im , S. Park , Coord. Chem. Rev. 2025, 540, 216761.

[44] M. Paulsson , S. Datta , Phys. Rev. B 2003, 67, 241403.

[45] J. A. Malen , S. K. Yee , A. Majumdar , R. A. Segalman , Chem. Phys. Lett. 2010, 491, 109.

[46] K. Baheti , J. A. Malen , P. Doak , P. Reddy , S.‐Y. Jang , T. D. Tilley , A. Majumdar , R. A. Segalman , Nano Lett. 2008, 8, 715. 18269258 10.1021/nl072738l

[47] H. Basch , R. Cohen , M. A. Ratner , Nano Lett. 2005, 5, 1668. 16159203 10.1021/nl050702s

[48] M. Hanke , H. K. Arslan , S. Bauer , O. Zybaylo , C. Christophis , H. Gliemann , A. Rosenhahn , C. Wöll , Langmuir 2012, 28, 6877. 22471238 10.1021/la300457z

[49] A. Summerfield , I. Cebula , M. Schröder , P. H. Beton , J. Phys. Chem. C 2015, 119, 23544. 10.1021/acs.jpcc.5b07133 PMC4689388 26709359

[50] T. P. Vello , M. Strauss , C. A. R. Costa , C. C. Corrêa , C. C. B. Bufon , Phys. Chem. Chem. Phys. 2020, 22, 5839. 32107524 10.1039/c9cp05717j

[51] A. Snow , G. Jernigan , M. Ancona , Analyst 2011, 136, 4935. 22005722 10.1039/c1an15664k

[52] M. Kjærvik , P. M. Dietrich , A. Thissen , J. Radnik , A. Nefedov , C. Natzeck , C. Wöll , W. E. Unger , J. Electron Spectrosc. Relat. Phenom. 2021, 247, 147042.

[53] P. St. Petkov , G. N. Vayssilov , J. Liu , O. Shekhah , Y. Wang , C. Wöll , T. Heine , ChemPhysChem 2012, 13, 2025. 22517762 10.1002/cphc.201200222

[54] Y. Nie , Y. Li , J. Li , L. Chen , X. Wang , T. Chen , Z. Cai , Colloids Surf. A: Physicochem. Eng. Asp. 2024, 680, 132742.

[55] V. Mugnaini , M. Tsotsalas , F. Bebensee , S. Grosjean , A. Shahnas , S. Bräse , J. Lahann , M. Buck , C. Wöll , Chem. Commun. 2014, 50, 11129. 10.1039/c4cc03521f 25105178

[56] W. Luo , L. Tang , X. Wang , S.‐J. Lin , Z. Cai , ACS Appl. Nano Mater. 2023, 6, 22406.

[57] C. Schneider , D. Ukaj , R. Koerver , A. A. Talin , G. Kieslich , S. P. Pujari , H. Zuilhof , J. Janek , M. D. Allendorf , R. A. Fischer , Chem. Sci. 2018, 9, 7405. 30542544 10.1039/c8sc02471e PMC6237122

[58] F. C. Simeone , H. J. Yoon , M. M. Thuo , J. R. Barber , B. Smith , G. M. Whitesides , J. Am. Chem. Soc. 2013, 135, 18131. 24187999 10.1021/ja408652h

[59] L. Cademartiri , M. M. Thuo , C. A. Nijhuis , W. F. Reus , S. Tricard , J. R. Barber , R. N. Sodhi , P. Brodersen , C. Kim , R. C. Chiechi , J. Phys. Chem. C 2012, 116, 10848.

[60] C. M. Bowers , K.‐C. Liao , H. J. Yoon , D. Rappoport , M. Baghbanzadeh , F. C. Simeone , G. M. Whitesides , Nano Lett. 2014, 14, 3521. 24840009 10.1021/nl501126e

[61] A. Vilan , J. Phys. Chem. C 2007, 111, 4431. 10.1021/jp071695u 17474730

[62] J. G. Simmons , J. Appl. Phys. 1963, 34, 2581.

[63] H. Ishii , K. Sugiyama , E. Ito , K. Seki , Adv. Mater. 1999, 11, 605.

[64] S. Braun , W. R. Salaneck , M. Fahlman , Adv. Mater. 2009, 21, 1450.

[65] M. T. Greiner , M. G. Helander , W.‐M. Tang , Z.‐B. Wang , J. Qiu , Z.‐H. Lu , Nat. Mater. 2012, 11, 76. 10.1038/nmat3159 22057388

[66] C. J. Lim , M. G. Park , M. S. Kim , J. H. Han , S. Cho , M.‐H. Cho , Y. Yi , H. Lee , S. W. Cho , Molecules 2018, 23, 449. 29463008 10.3390/molecules23020449 PMC6017094

[67] C. L. Mthembu , R. C. Chiechi , Nano Lett. 2024, 24, 10921. 39186321 10.1021/acs.nanolett.4c02783

[68] J. Liu , B. van der Zee , R. Alessandri , S. Sami , J. Dong , M. I. Nugraha , A. J. Barker , S. Rousseva , L. Qiu , X. Qiu , Nat. Commun. 11, 2020, 5694. 33173050 10.1038/s41467-020-19537-8 PMC7655812

[69] E. A. Weiss , G. K. Kaufman , J. K. Kriebel , Z. Li , R. Schalek , G. M. Whitesides , Langmuir 2007, 23, 9686. 17696377 10.1021/la701919r

[70] K. J. Erickson , F. Léonard , V. Stavila , M. E. Foster , C. D. Spataru , R. E. Jones , B. M. Foley , P. E. Hopkins , M. D. Allendorf , A. A. Talin , Adv. Mater. 2015, 27, 3453. 25925161 10.1002/adma.201501078

[71] Z. Wang , D. Nminibapiel , P. Shrestha , J. Liu , W. Guo , P. G. Weidler , H. Baumgart , C. Wöll , E. Redel , ChemNanoMat 2, 2016, 67.

[72] R. C. Chiechi , E. A. Weiss , M. D. Dickey , G. M. Whitesides , Angew. Chem., Int. Ed. 2008, 47, 142. 10.1002/anie.200703642 18038438

[73] J. Blatt , Thermoelectric power of metals, Springer Science & Business Media, New York, 2012,

