# Supplementary material for: Unveiling Thermoelectric Properties of SURMOF Nanofilms: A New Frontier in Molecular Thermoelectrics
Source: Adv Sci (Weinh). 2025 Aug 31;12(44):e10730. doi: 10.1002/advs.202510730 (PMC12667524; doi:10.1002/advs.202510730)
Supplement: Supplementary file 1 — Supporting Information [file ADVS-12-e10730-s001.pdf]

# Supporting Information

## Unveiling Thermoelectric Properties of SURMOF Nanofilms: A New Frontier in Molecular Thermoelectrics

Jiwoo Park,[1] Soo Jin Cho,[2] Dong Su Lee,[2] Sohyun Park[1,3]*

[1] School of Chemistry and Energy, Sungshin Women's University, Seoul, 01133, Republic of Korea
[2] Institute of Advanced Composite Materials, Korea Institute of Science and Technology (KIST), Wanju-gun, Jeonbuk, 55324, Republic of Korea
[3] Center for NanoBio Applied Technology, Sungshin Women's University, Seoul, 01133, Republic of Korea

*Corresponding author's email: sohyun@sungshin.ac.kr

**Table of Contents**

**1. Further Discussions**

1.1 Loading Conditions------------------------------------------------------------------------ 3

1.2 Grazing Incidence X-ray Diffraction (GI-XRD)---------------------------------------4

1.3 X-ray Photoelectron Spectroscopy (XPS)----------------------------------------------4

**2. Supplementary Figures and Tables**------------------------------------------------------5

**3. References**---------------------------------------------------------------------------------61

## 1. Further Discussions

### 1.1 Loading Conditions

The guest molecule solution was prepared by dissolving 0.1 mM ferrocene (Fc) or 2 mM tetracyanoquinodimethane (TCNQ) in ethanol. To determine optimal loading conditions, dried HKUST-1 nanofilms were immersed in guest molecule solutions with varying concentrations and soaking durations. Current density measurements were conducted to identify the conditions under which guest molecule incorporation was maximized. All HKUST-1 nanofilms used in the loading optimization process were fabricated with three layer-by-layer growth cycles. For Fc loading, HKUST-1 nanofilms were immersed in Fc solutions ranging from 0.05 mM to 1 mM for 24 hours, and current density measurements were performed (Figure S1). The analysis of the $\log J((+0.5\ V))_{mean}$ values as a function of concentration indicated a maximum at 0.1 mM, which was subsequently fixed for further experiments. The soaking time was then varied between 10 minutes and 48 hours, with the highest $\log J((+0.5\ V))_{mean}$ observed at 24 hours (Figure S2). Therefore, Fc incorporation was optimized at 0.1 mM and 24 hours of soaking. A similar optimization procedure was applied for TCNQ loading. HKUST-1 nanofilms were immersed in TCNQ solutions at concentrations ranging from 0.05 mM to 2 mM for 24 hours, and current density measurements were obtained (Figure S3). The highest $\log J((+0.5\ V))_{mean}$ was recorded at 2 mM, which was then used for further soaking time adjustments. Soaking durations varied from 2 to 72 hours, and the maximum current density was reached at 24 hours of immersion (Figure S4). Consequently, TCNQ incorporation was optimized at 2 mM and 24 hours of soaking.

### 1.2 Grazing Incidence X-ray Diffraction (GI-XRD)

The XRD pattern in Figure S5 exhibits consistency with the preferred orientation of HKUST-1 grown on COOH-functionalized surfaces, as reported in [1, 2, 3]. Previous study have shown that HKUST-1 grown on long alkyl chains tends to adopt a preferred [100] orientation.[4] As depicted in Figure S5, the XRD pattern of HKUST-1(10) demonstrates a higher peak intensity for the [100] plane compared to HKUST-1(2), which can be attributed to the increased packing density of longer alkyl chains, leading to the formation of well-defined octahedral crystallites. In contrast, short alkyl chains exhibit weaker van der Waals interactions due to their lower packing density, resulting in poor alignment of the terminal -COOH groups and a predominance of triangular [111] facets.[4]

### 1.3 X-ray Photoelectron Spectroscopy (XPS)

Photoelectron spectra were analyzed using the XPS Peak Fit program, with a linear background applied for spectral deconvolution. High-resolution XP spectra were obtained for HKUST-1(2), including Au 4f, C 1s, Cu $2p_{3/2}$, O 1s, and S 2p core levels. Additionally, Fe 2p and N 1s spectra were recorded for Fc@HKUST-1(2) and TCNQ@HKUST-1(2), respectively. Figures S8–S28 present the high-resolution spectra of both loaded and unloaded HKUST-1(2) across 1 to 7 deposition cycles.

The Au 4f spectrum was deconvoluted into doublet peaks at 84.0 ± 0.1 eV and 87.7 ± 0.1 eV, corresponding to the $4f_{5/2}$ and $4f_{7/2}$ states, of the $Au^{TS}$ substrate, respectively.[5, 6] The O 1s spectrum was deconvoluted into two distinct peaks at 531.4 ± 0.2 eV and 533.0 ± 0.3 eV, assigned to $COO^{-}$ functional groups and C-$O_X$ species, respectively, with the latter indicative of adsorbed $H_2O$.[7]

## 2. Supplementary Figures and Tables

**Figure S1.** (a) Histograms of current density values measured after immersing dried HKUST-1(2) nanofilms in ethanol-based Fc solutions of varying concentrations for 24 hours. $N_{junction}$ is the number of junctions measured, $N_{traces}$ indicates the number of $J$-$V$ traces collected, $\log(J(+0.5\ V))_{mean}$ denotes the mean value, $\sigma_{\log J((+0.5\ V))}$ represents the standard deviation, obtained via Gaussian fitting of the histogram. (b) Plot of $\log(J(+0.5\ V))_{mean}$ as a function of Fc concentration in solution.

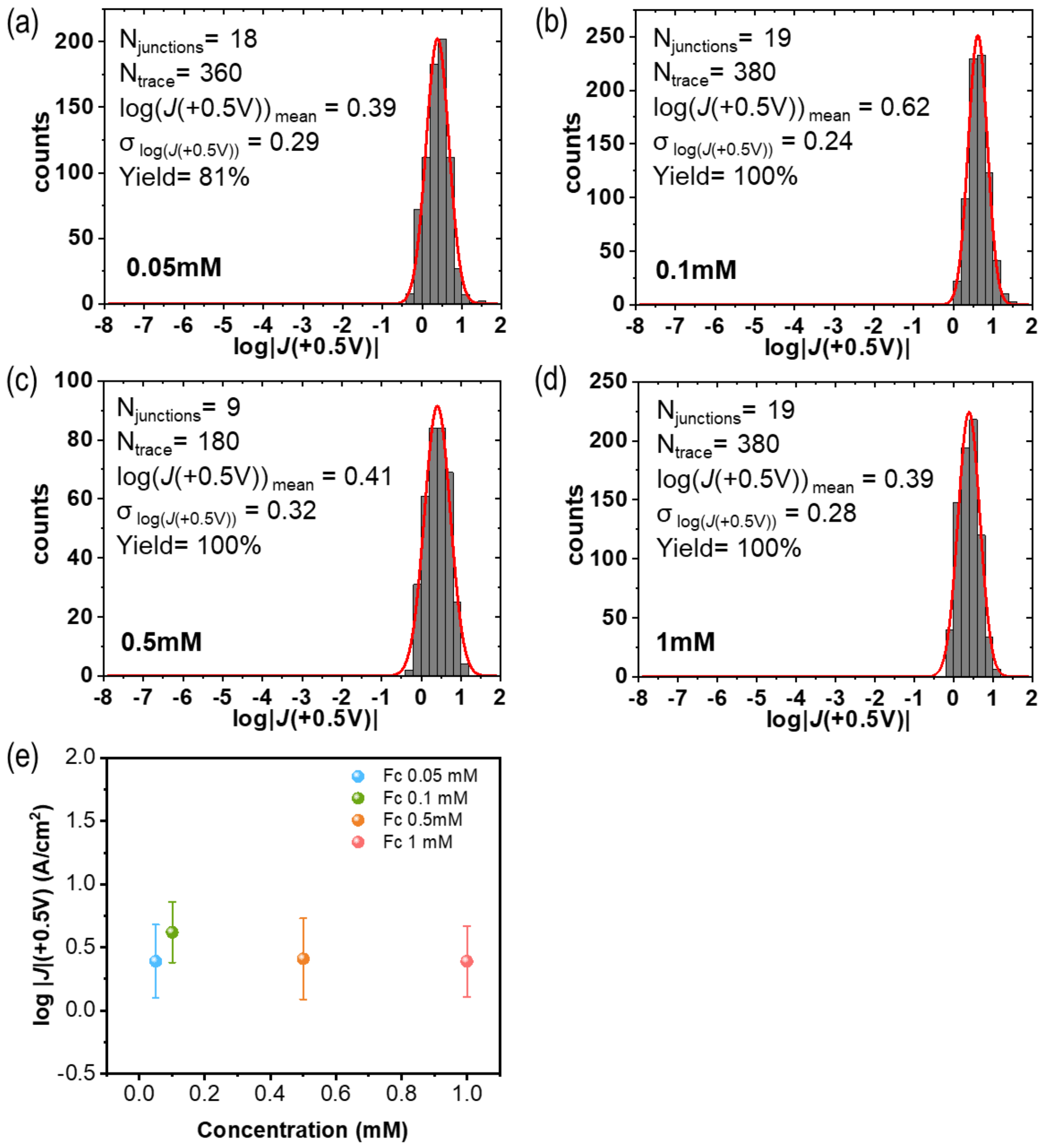

**Figure S2.** Histograms of current density values measured after immersing dried HKUST-1(2) nanofilms in an ethanol solution containing 0.1 mM Fc for different durations. (b) Plot of log($J$(+0.5 V))$_{mean}$ as a function of loading time.

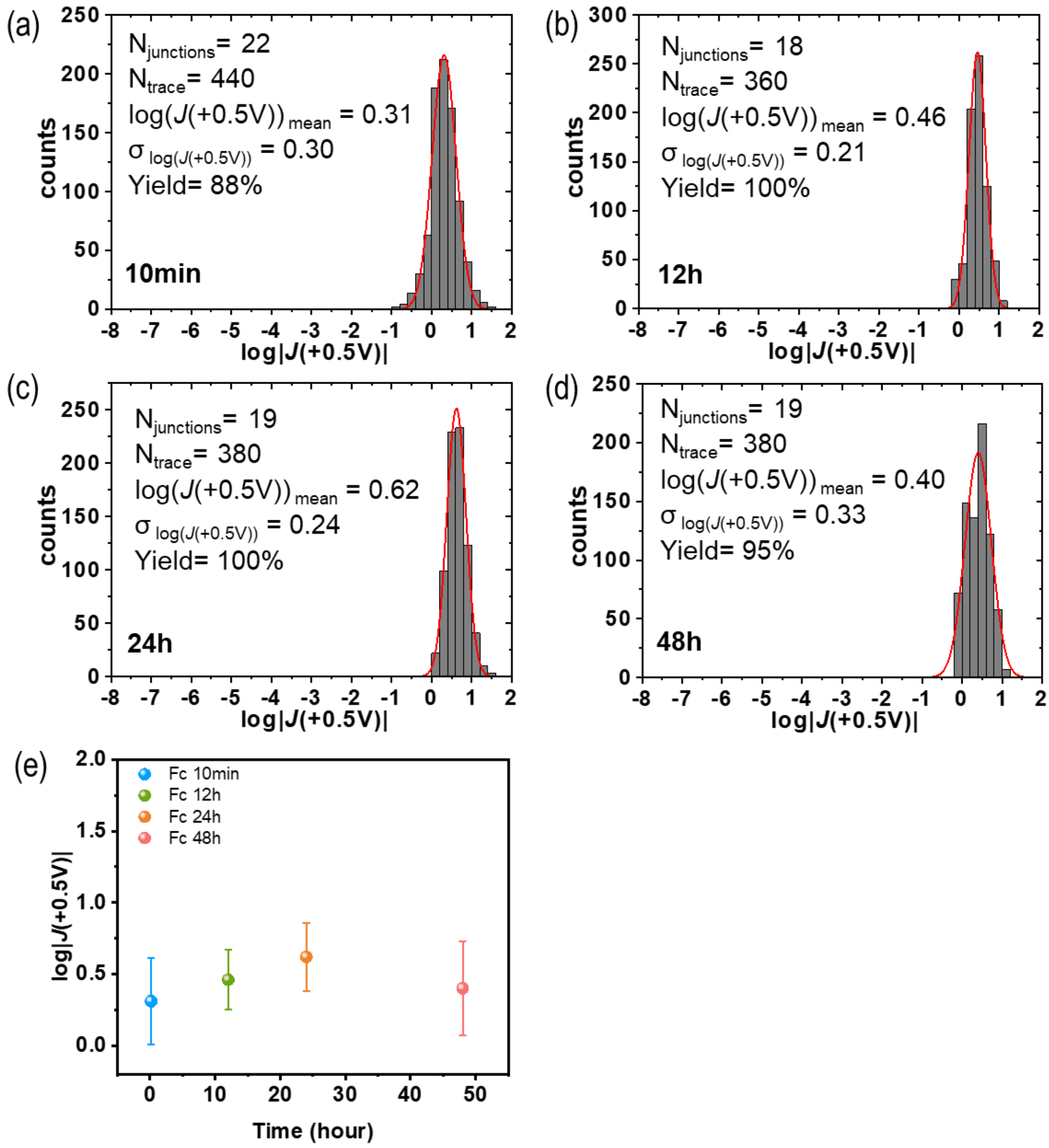

**Figure S3.** Histograms of current density values measured after immersing dried HKUST-1(2) nanofilms in ethanol-based TCNQ solutions of varying concentrations for 24 hours. (b) Plot of log($J$(+0.5 V))$_{mean}$ as a function of TCNQ concentration in solution.

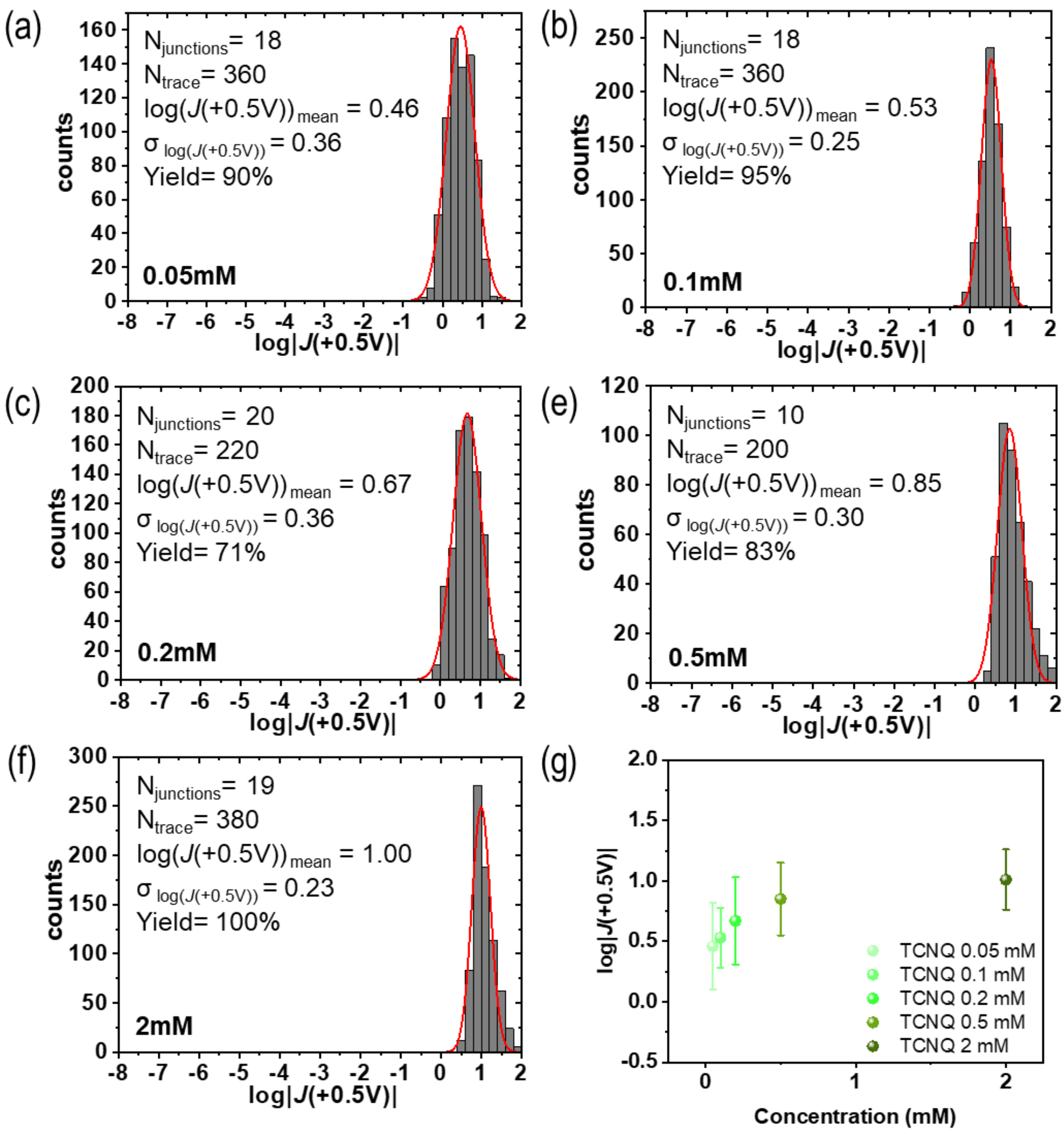

**Figure S4.** Histograms of current density values measured after immersing dried HKUST-1(2) nanofilms in an ethanol solution containing 2 mM TCNQ for different durations. (b) Plot of log($J$(+0.5 V))$_{mean}$ as a function of loading time.

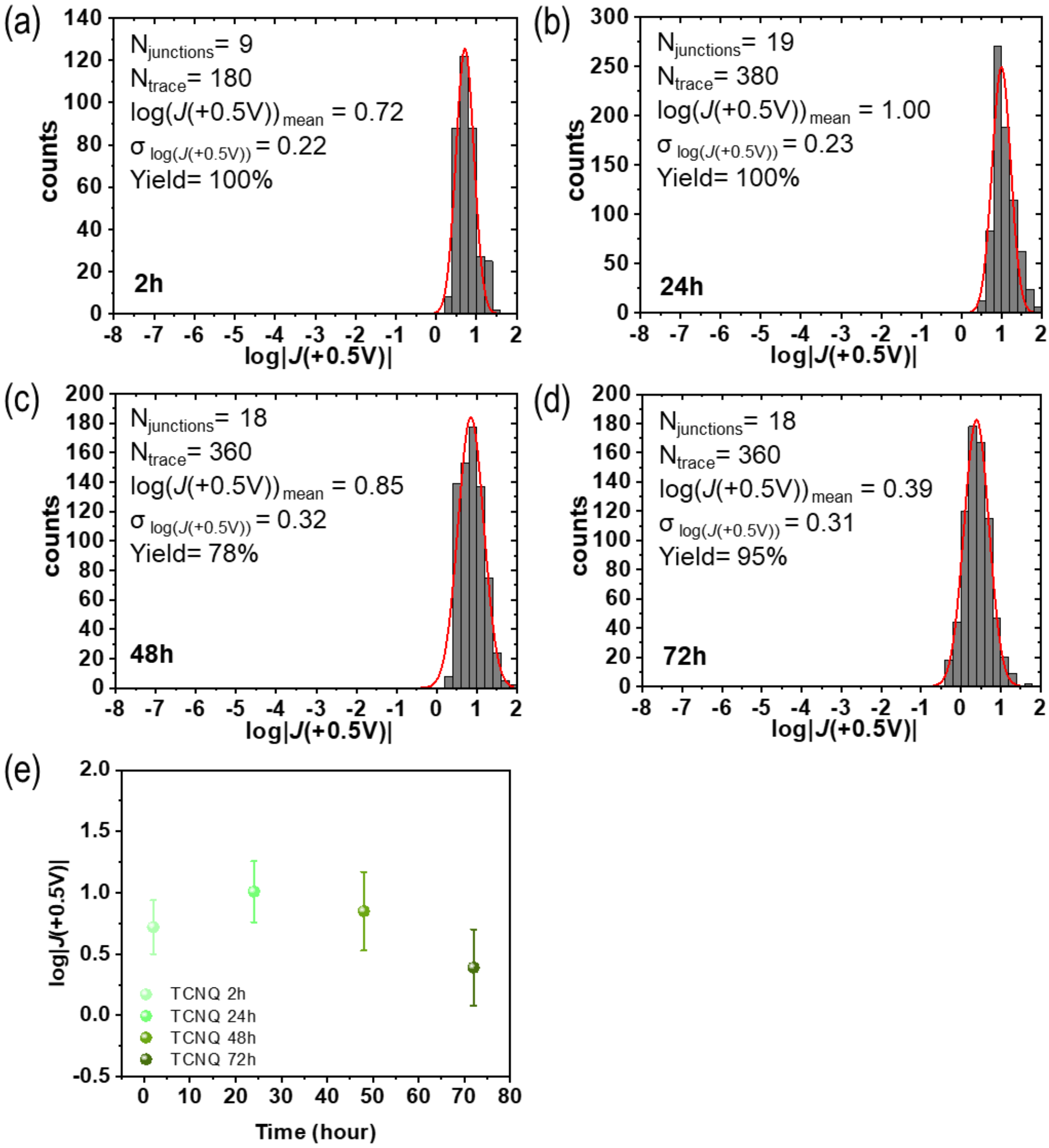

**Figure S5**. Grazing incidence X-ray diffraction (GI-XRD) pattern of HKUST-1(2) and HKUST-1(10) at 7 cycles.

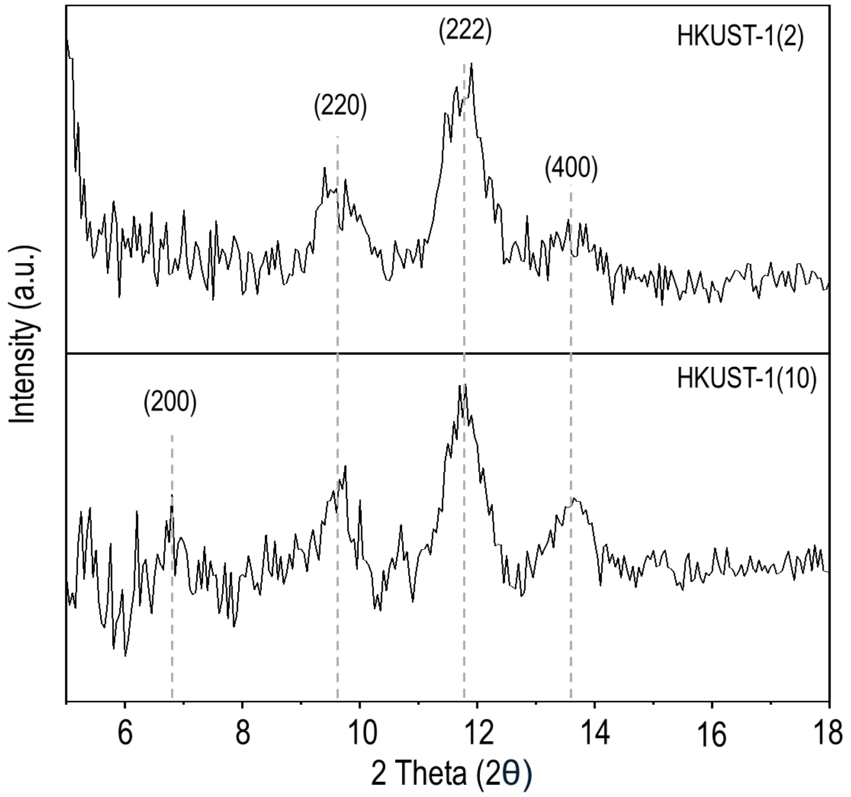

**Figure S6.** AFM surface images of HKUST-1(2) from 1 to 7 cycles.

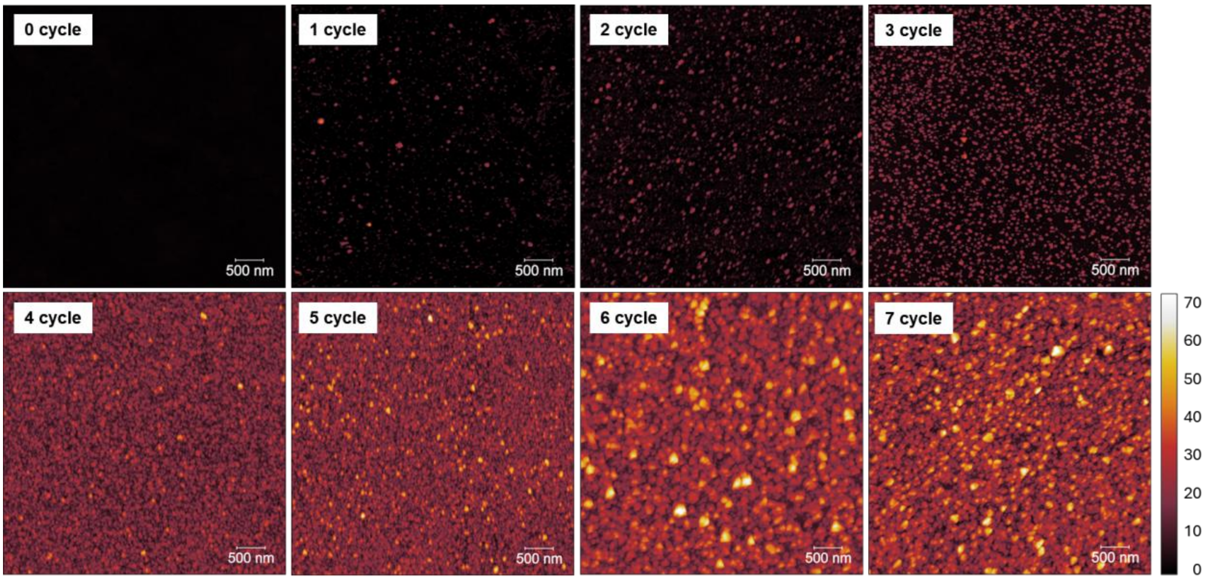


**Figure S7.** AFM surface images of HKUST-1(10) from 1 to 7 cycles.

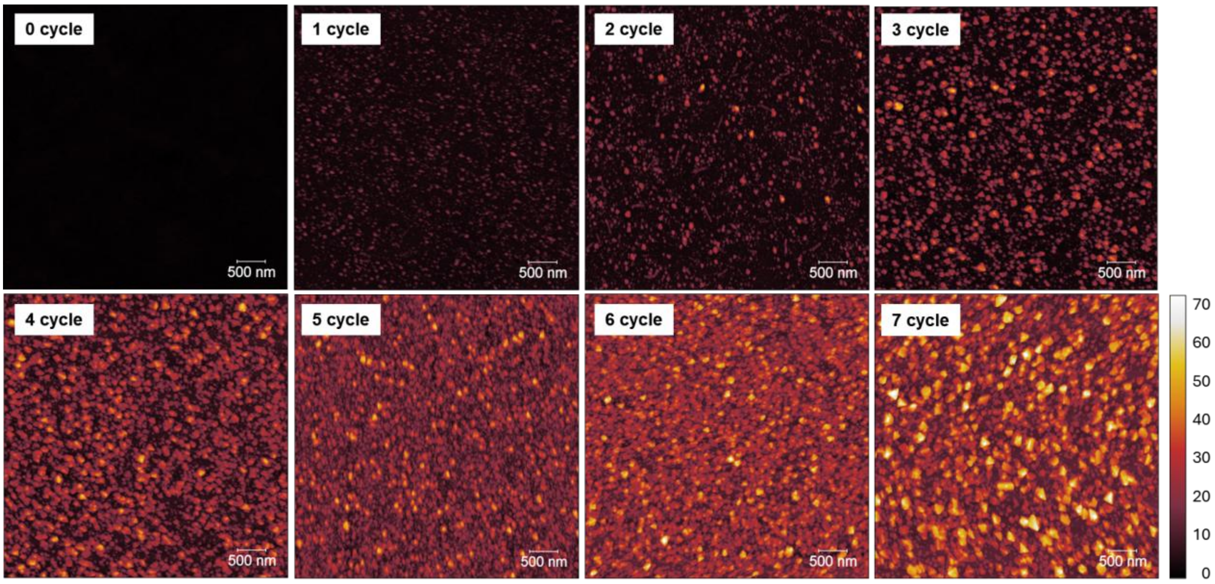

**Figure S8.** XPS high resolution spectra (Au 4f, C 1s, Cu $2p_{3/2}$, O 1s, and S 2p) for HKUST-1(2) at 1 cycle on $Au^{TS}$.

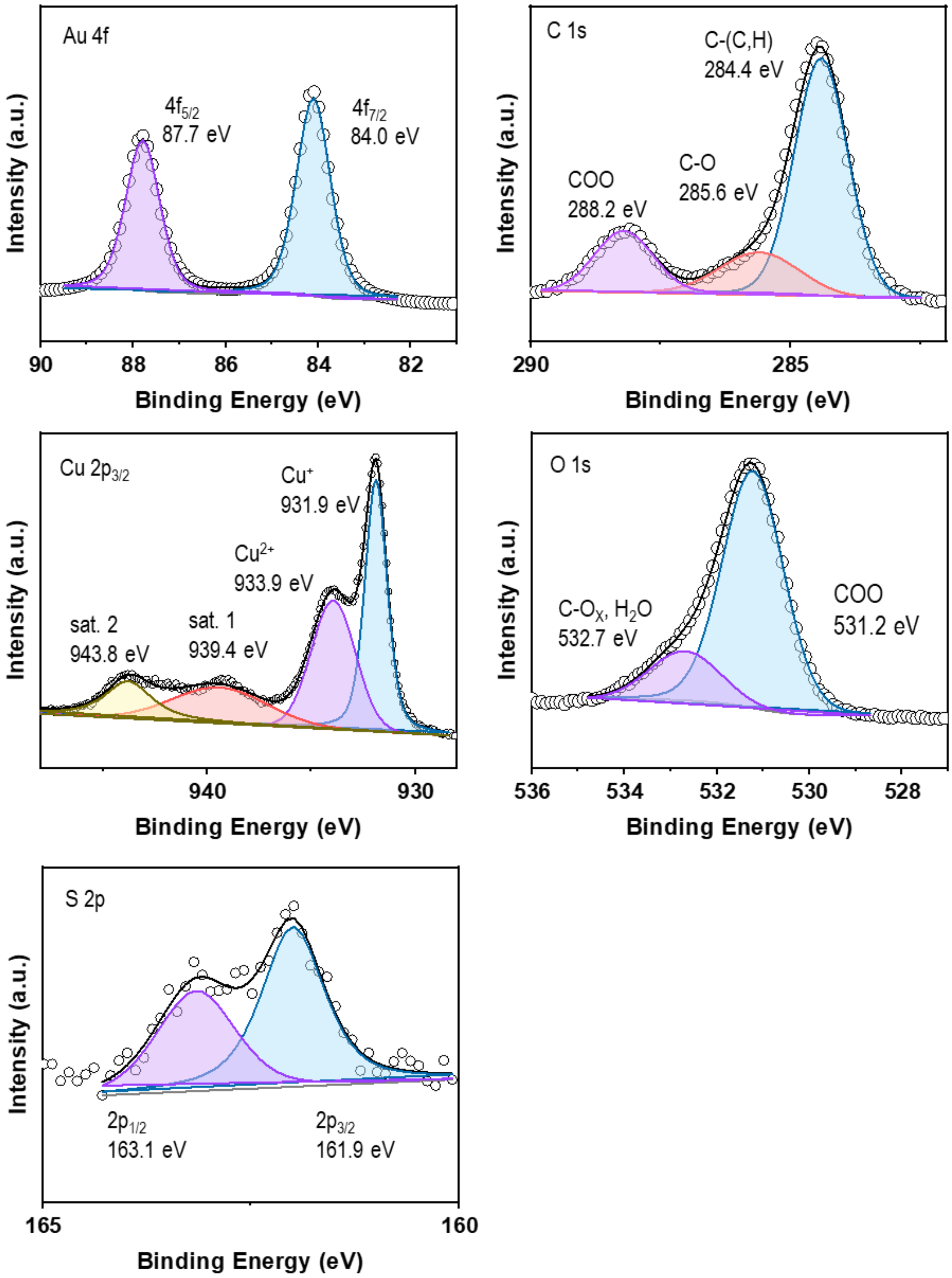

**Figure S9.** XPS high resolution spectra (Au 4f, C 1s, Cu $2p_{3/2}$, O 1s, and S 2p) for HKUST-1(2) at 2 cycles on $Au^{TS}$.

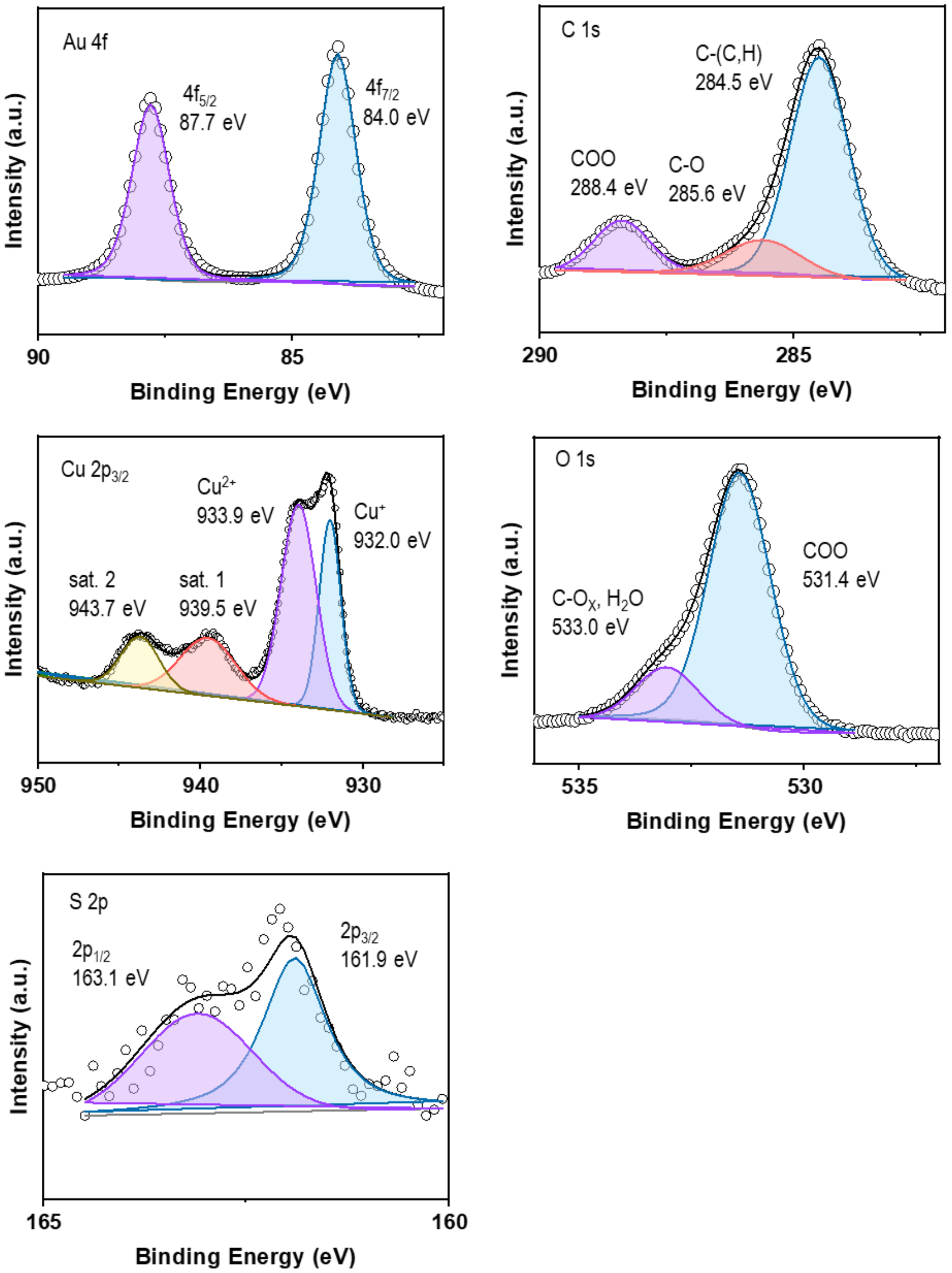

**Figure S10.** XPS high resolution spectra (Au 4f, C 1s, Cu $2p_{3/2}$, O 1s, and S 2p) for HKUST-1(2) at 3 cycles on $Au^{TS}$.

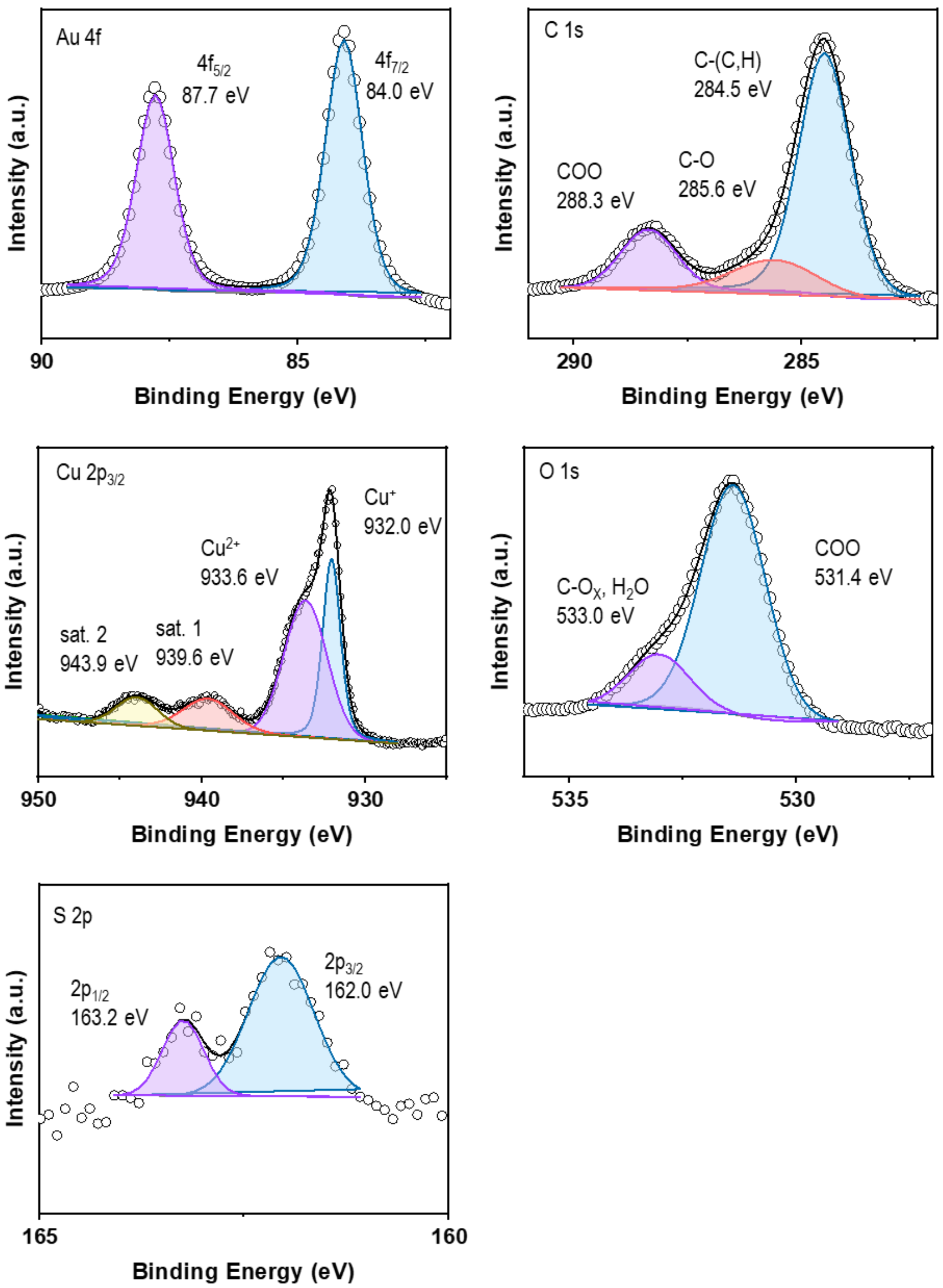

**Figure S11.** XPS high resolution spectra (Au 4f, C 1s, Cu $2p_{3/2}$, O 1s, and S 2p) for HKUST-1(2) at 4 cycles on $Au^{TS}$.

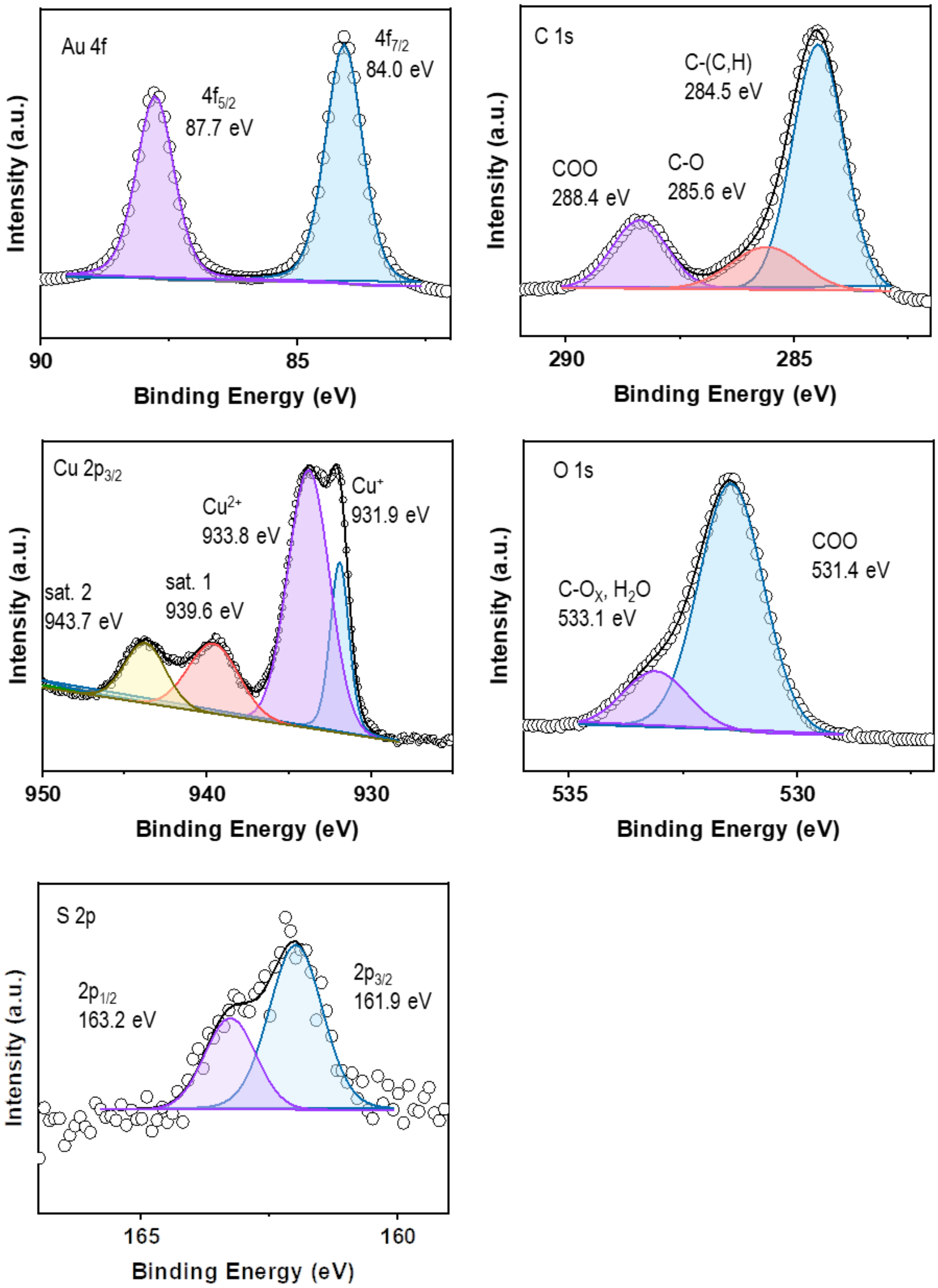

**Figure S12.** XPS high resolution spectra (Au 4f, C 1s, Cu $2p_{3/2}$, O 1s, and S 2p) for HKUST-1(2) at 5 cycles on $Au^{TS}$.

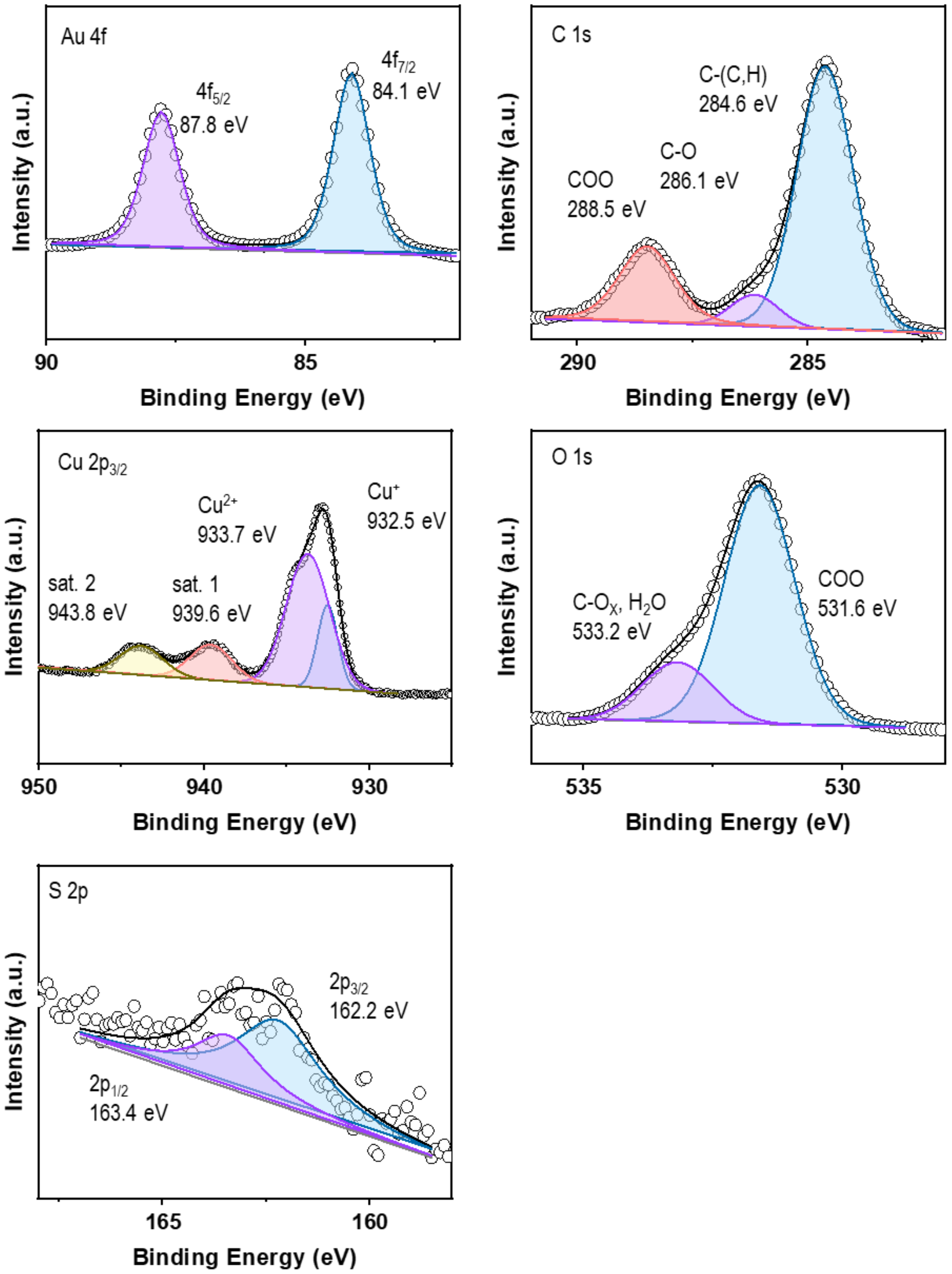

**Figure S13.** XPS high resolution spectra (Au 4f, C 1s, Cu $2p_{3/2}$, O 1s, and S 2p) for HKUST-1(2) at 6 cycles on $Au^{TS}$.

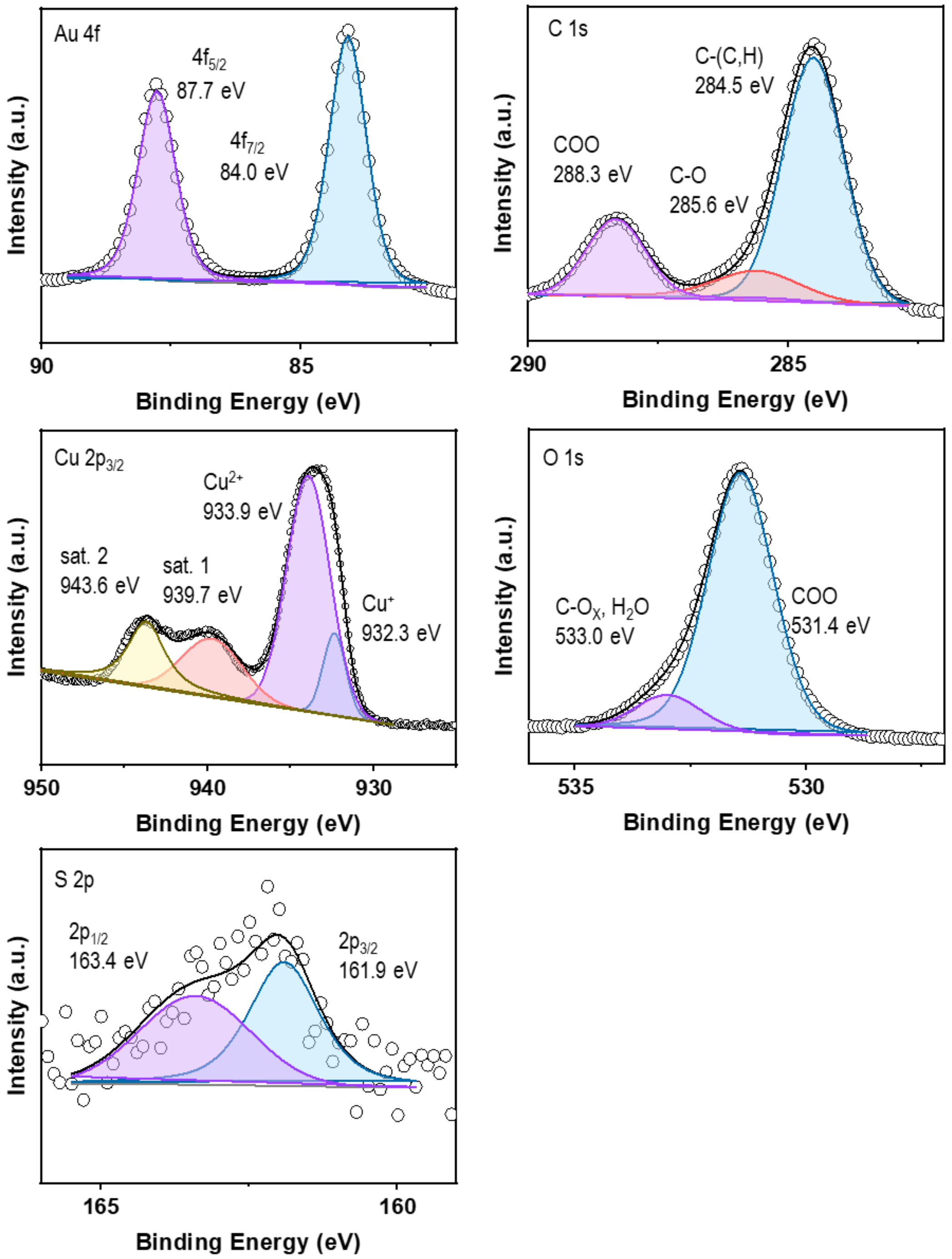

**Figure S14.** XPS high resolution spectra (Au 4f, C 1s, Cu $2p_{3/2}$, O 1s, and S 2p) for HKUST-1(2) at 7 cycles on $Au^{TS}$.

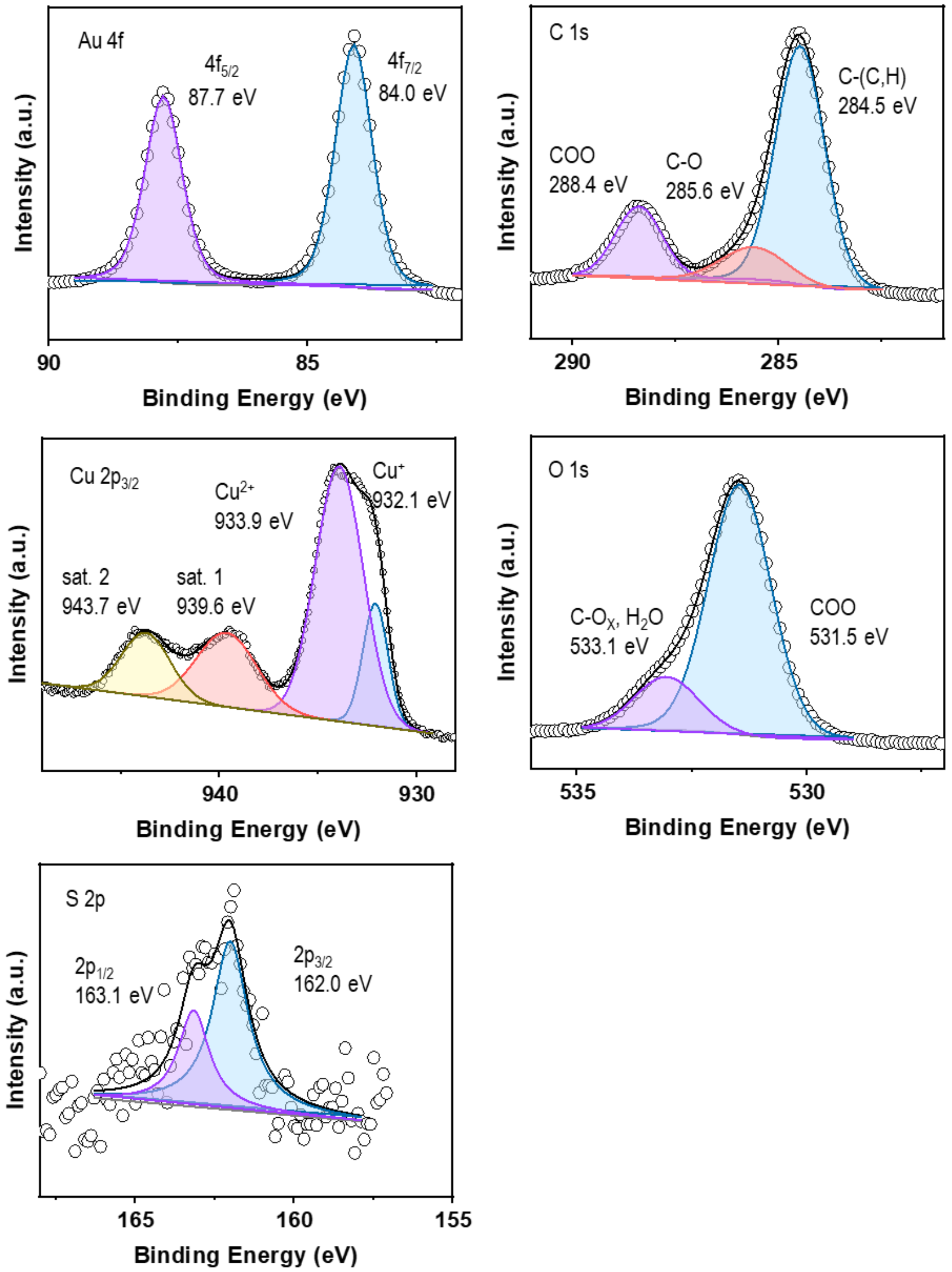

**Figure S15.** XPS high resolution spectra (Au 4f, C 1s, Cu $2p_{3/2}$, Fe 2p, O 1s, and S 2p) for Fc@HKUST-1(2) at 1 cycle on $Au^{TS}$.

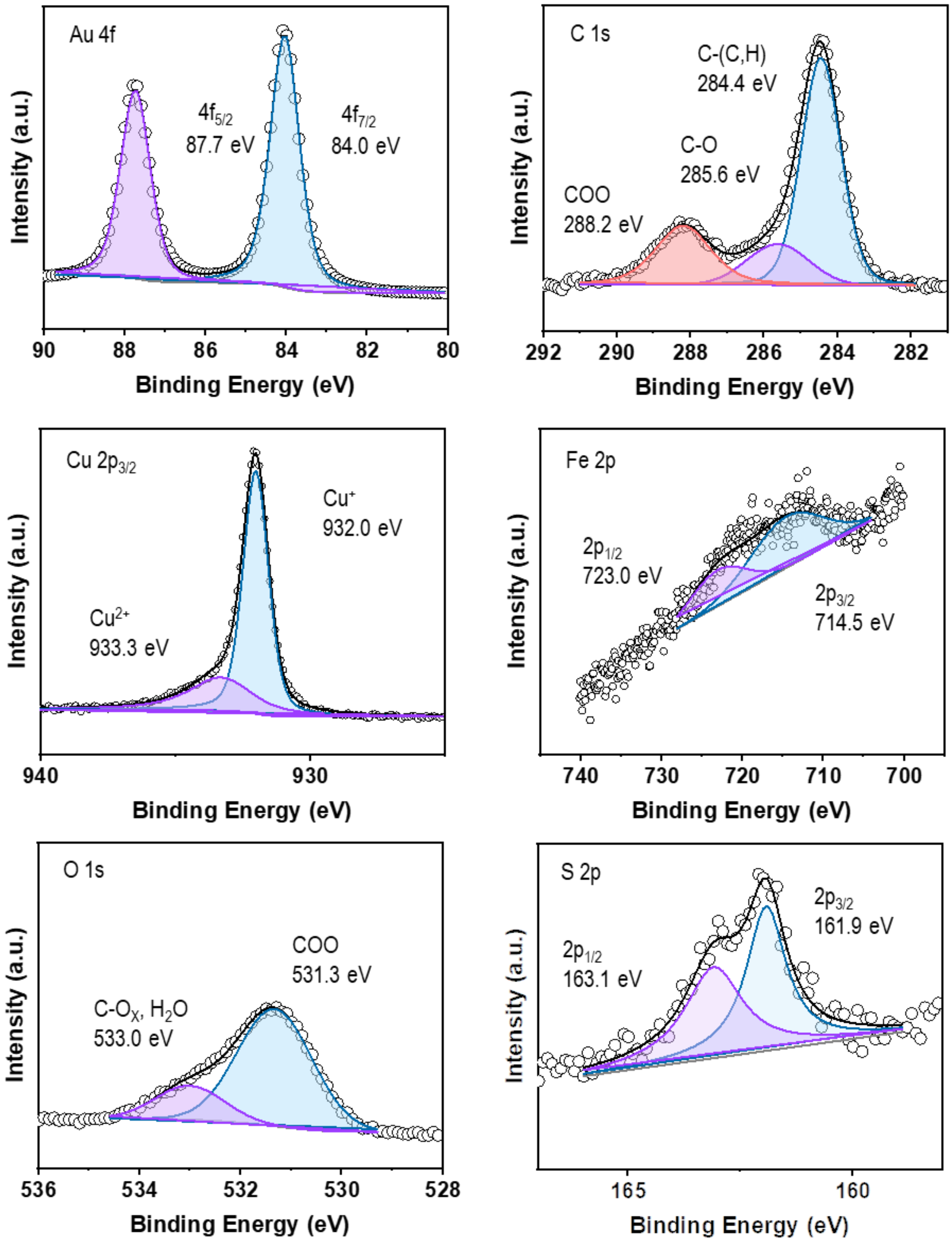

**Figure S16.** XPS high resolution spectra (Au 4f, C 1s, Cu $2p_{3/2}$, Fe 2p, O 1s, and S 2p) for Fc@HKUST-1(2) at 2 cycles on $Au^{TS}$.

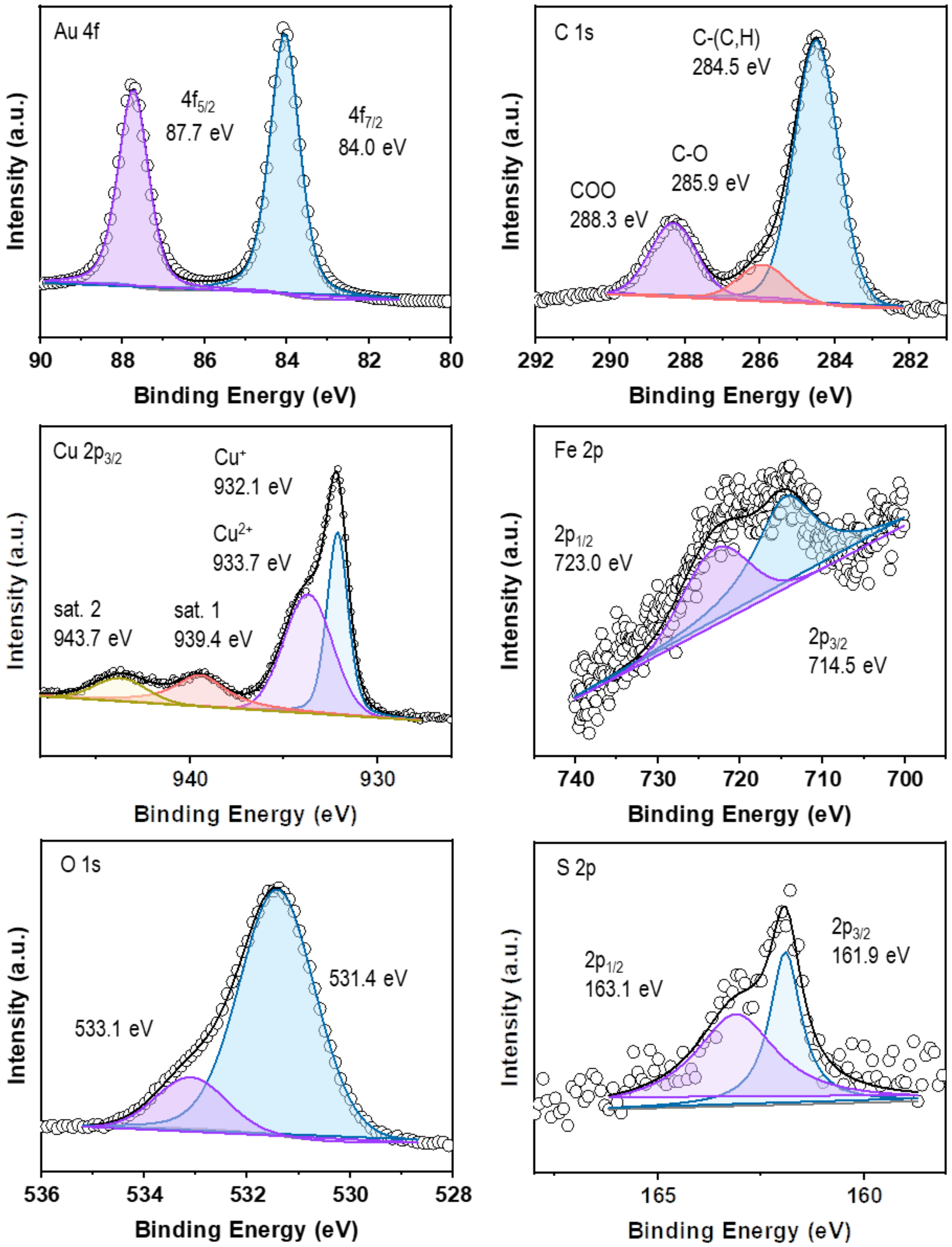

**Figure S17.** XPS high resolution spectra (Au 4f, C 1s, Cu $2p_{3/2}$, Fe 2p, O 1s, and S 2p) for Fc@HKUST-1(2) at 3 cycles on $Au^{TS}$.

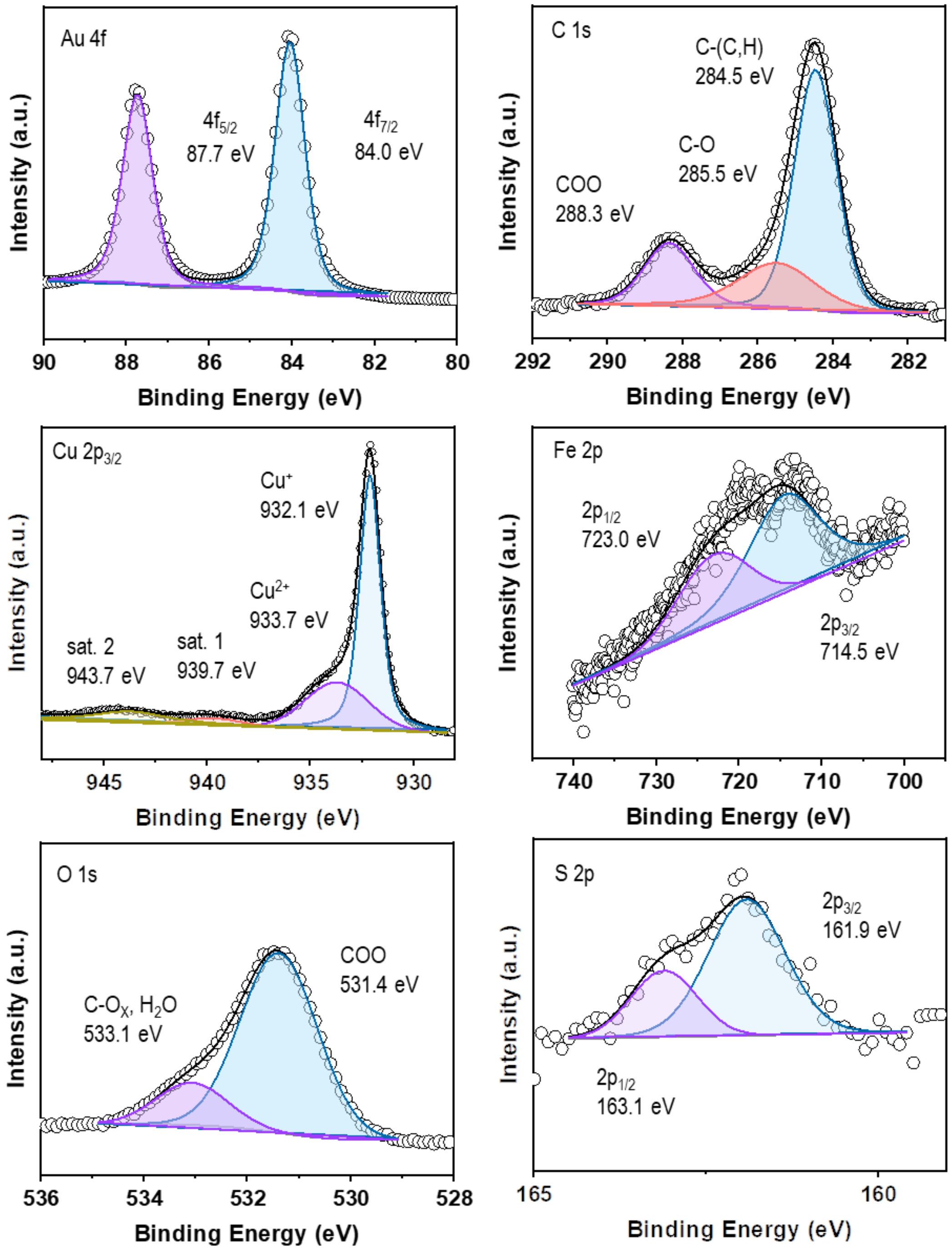

**Figure S18.** XPS high resolution spectra (Au 4f, C 1s, Cu $2p_{3/2}$, Fe 2p, O 1s, and S 2p) for Fc@HKUST-1(2) at 4 cycles on $Au^{TS}$.

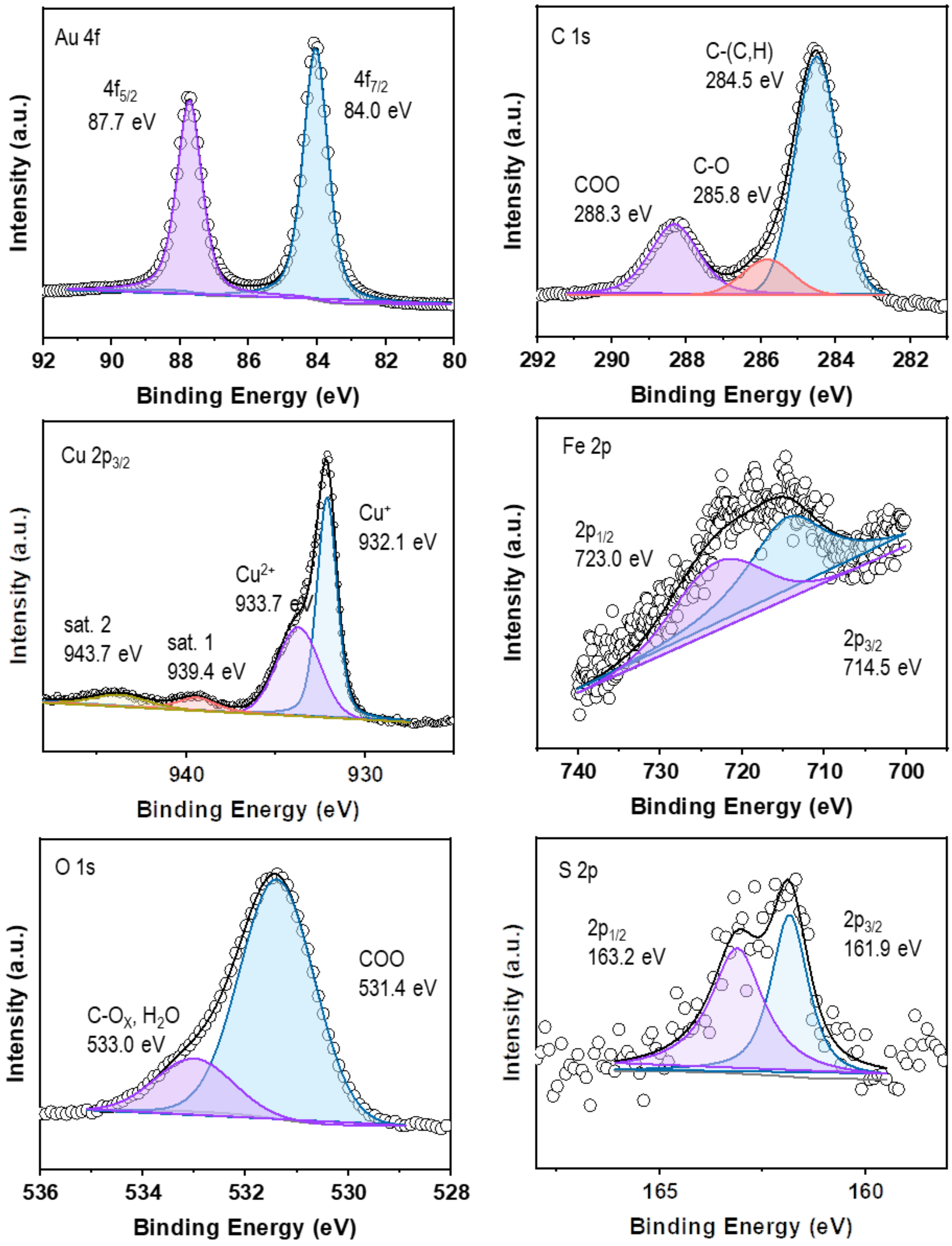

**Figure S19.** XPS high resolution spectra (Au 4f, C 1s, Cu $2p_{3/2}$, Fe 2p, O 1s, and S 2p) for Fc@HKUST-1(2) at 5 cycles on $Au^{TS}$.

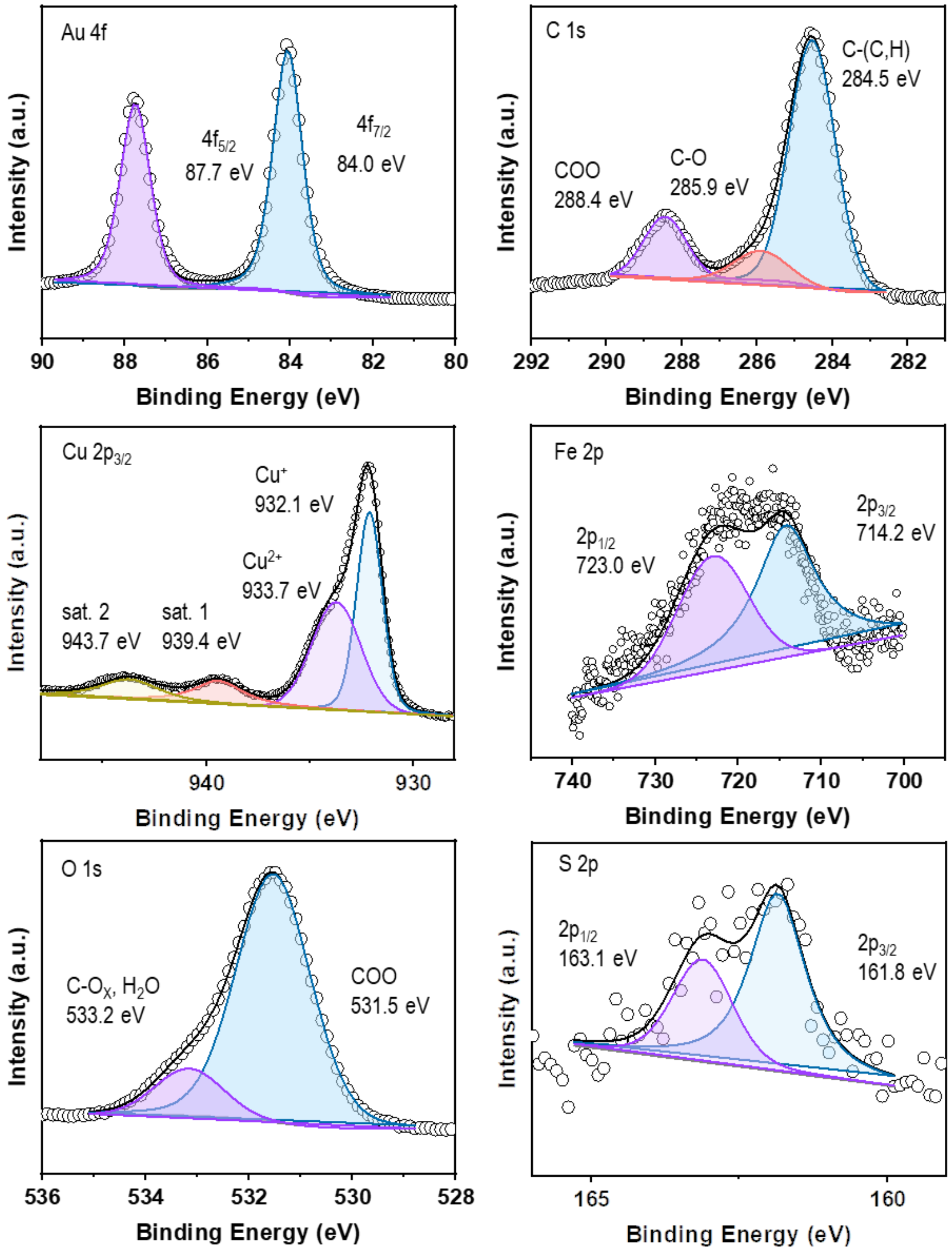

**Figure S20.** XPS high resolution spectra (Au 4f, C 1s, Cu $2p_{3/2}$, Fe 2p, O 1s, and S 2p) for Fc@HKUST-1(2) at 6 cycles on $Au^{TS}$.

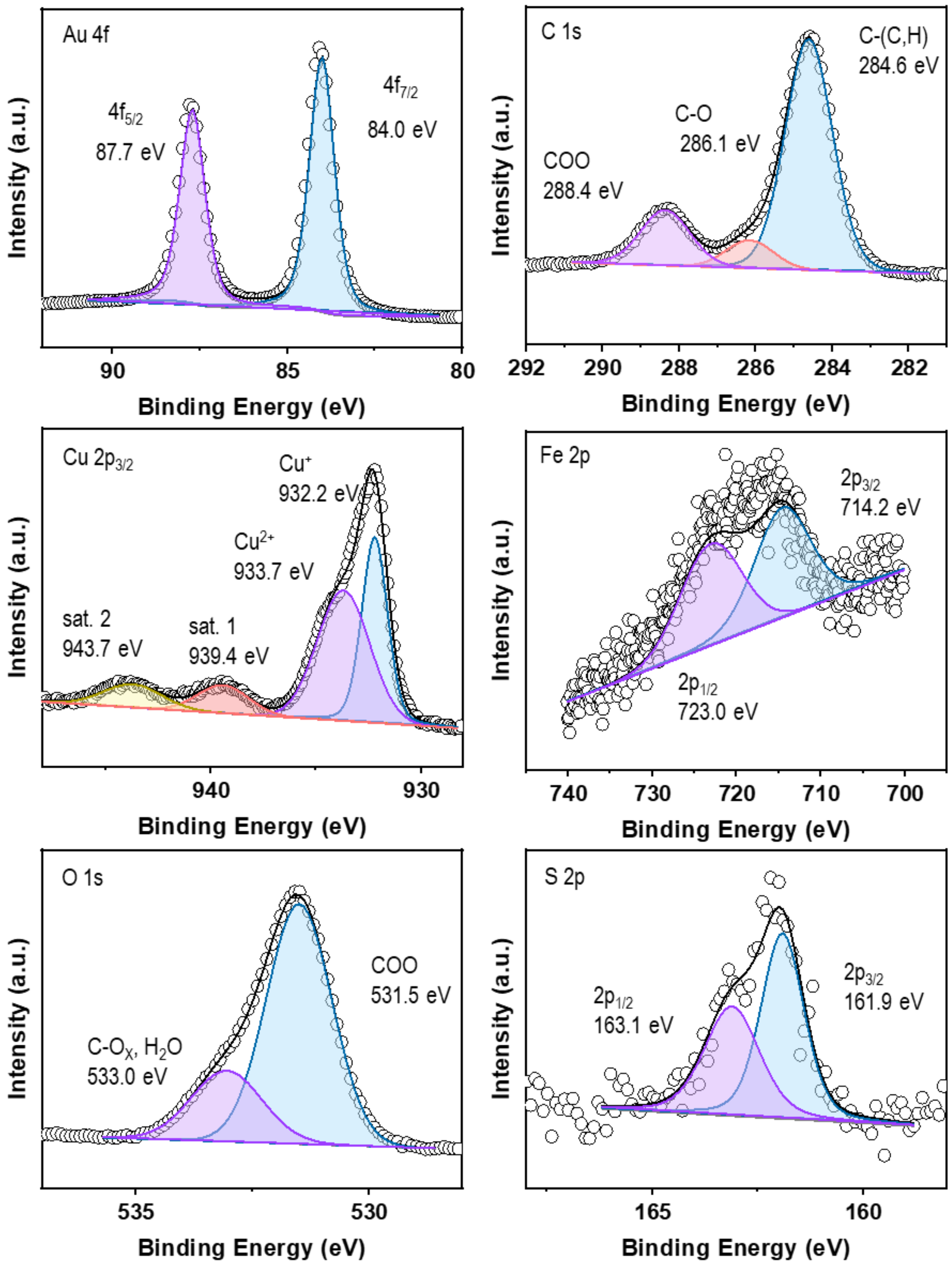

**Figure S21.** XPS high resolution spectra (Au 4f, C 1s, Cu $2p_{3/2}$, Fe 2p, O 1s, and S 2p) for Fc@HKUST-1(2) at 7 cycles on $Au^{TS}$.

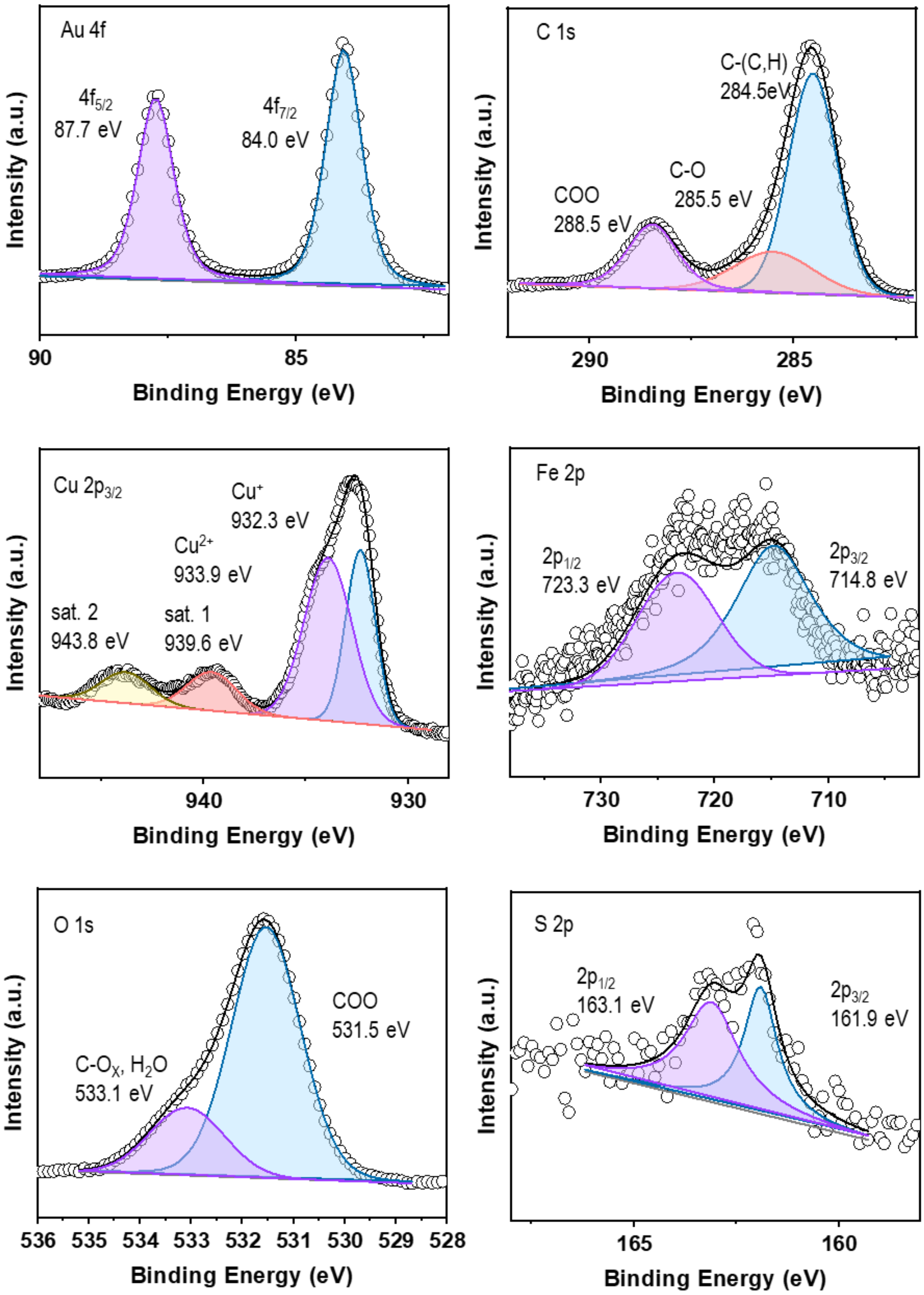

**Figure S22.** XPS high resolution spectra (Au 4f, C 1s, Cu $2p_{3/2}$, O 1s, N 1s and S 2p) for TCNQ@HKUST-1(2) at 1 cycle on $Au^{TS}$.

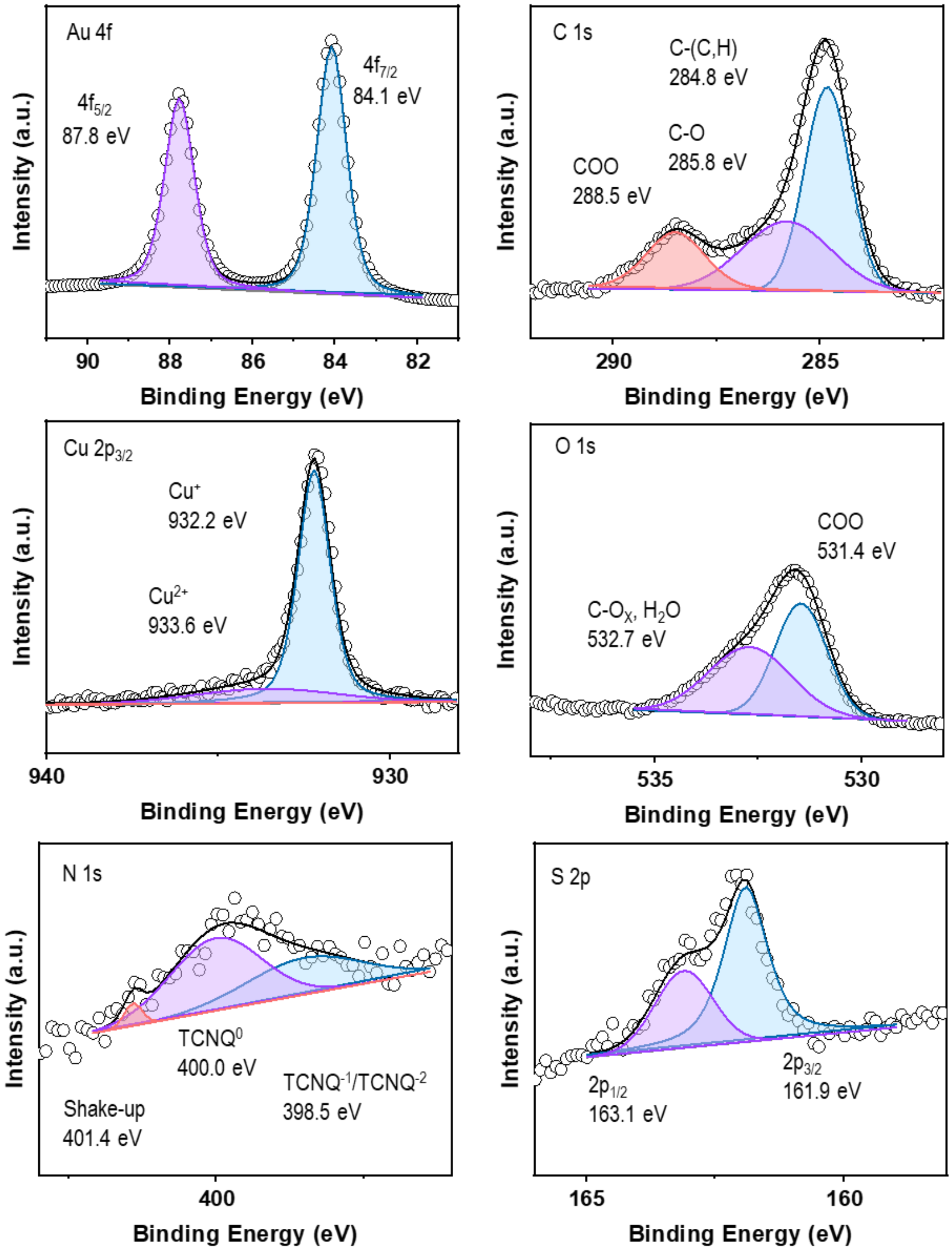

**Figure S23.** XPS high resolution spectra (Au 4f, C 1s, Cu $2p_{3/2}$, O 1s, N 1s and S 2p) for TCNQ@HKUST-1(2) at 2 cycles on $Au^{TS}$.

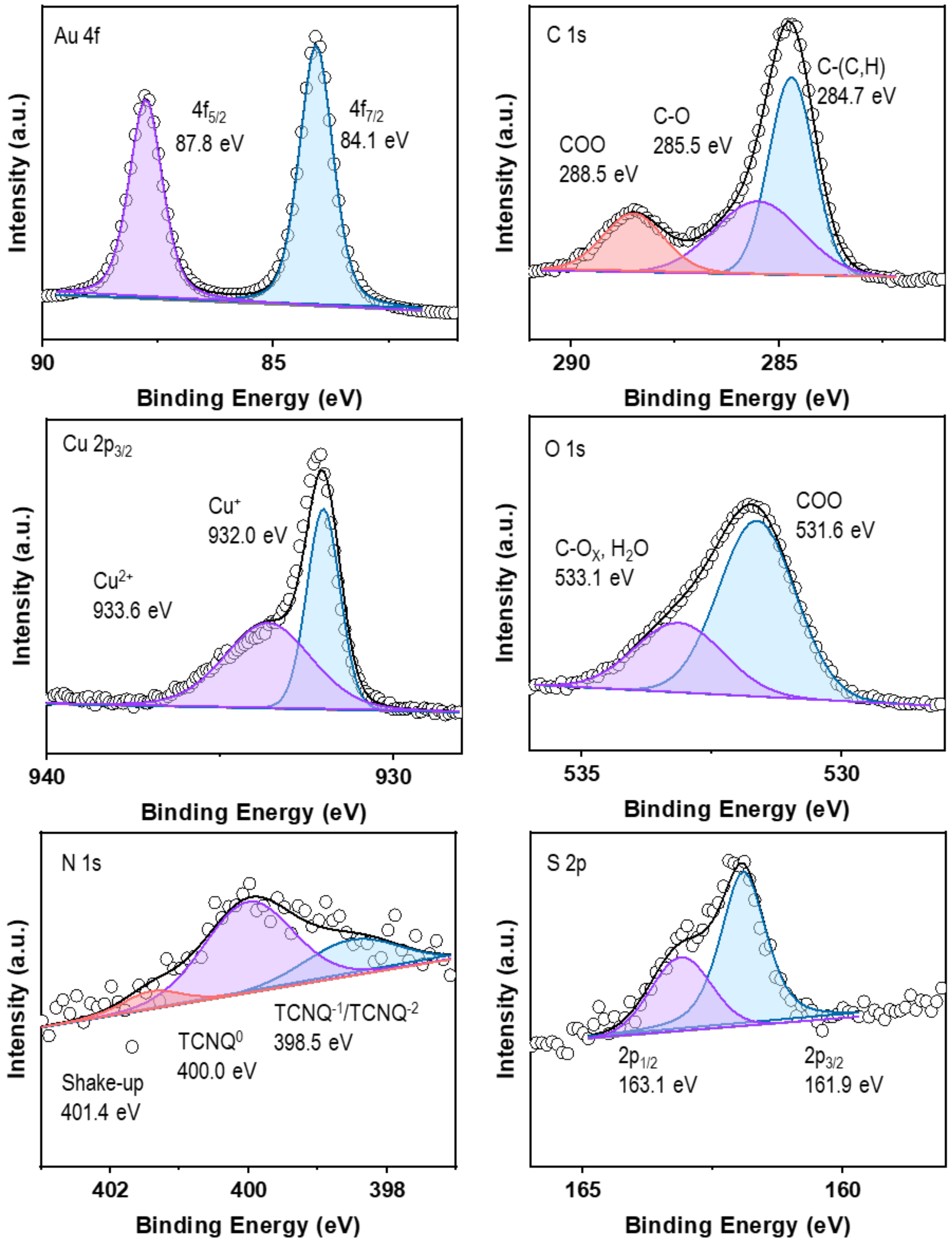

**Figure S24.** XPS high resolution spectra (Au 4f, C 1s, Cu $2p_{3/2}$, O 1s, N 1s and S 2p) for TCNQ@HKUST-1(2) at 3 cycles on $Au^{TS}$.

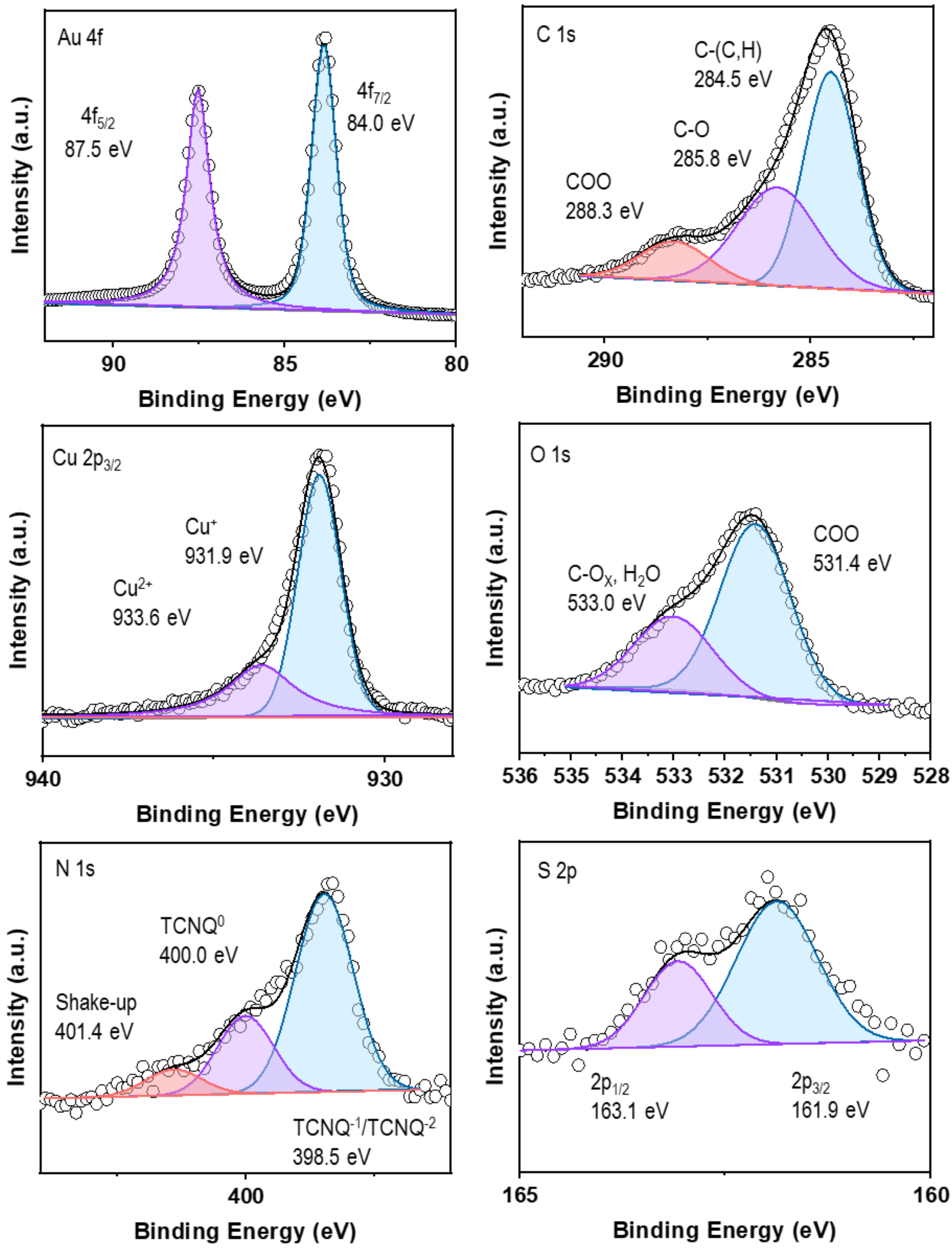

**Figure S25.** XPS high resolution spectra (Au 4f, C 1s, Cu $2p_{3/2}$, O 1s, N 1s and S 2p) for TCNQ@HKUST-1(2) at 4 cycles on $Au^{TS}$.

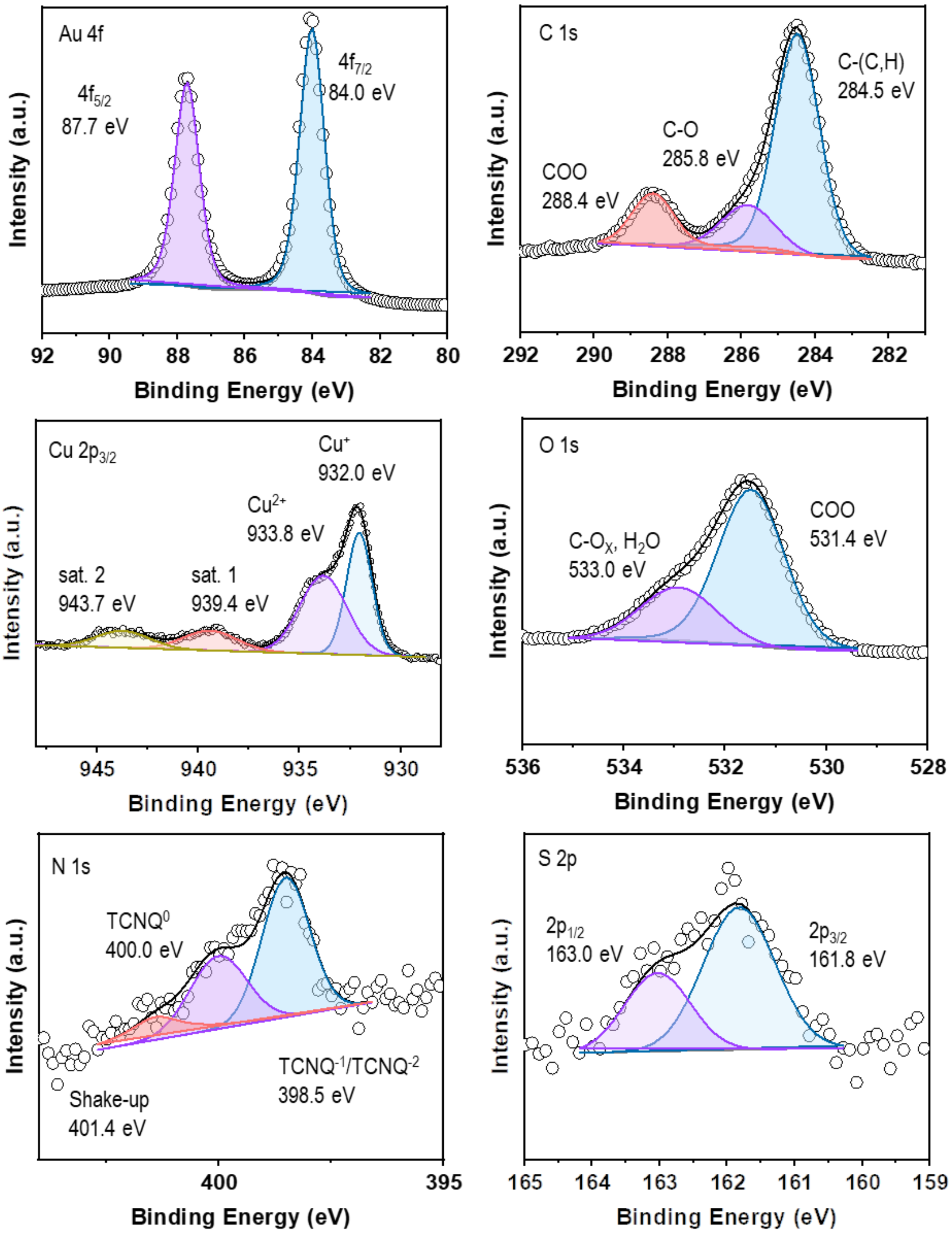

**Figure S26.** XPS high resolution spectra (Au 4f, C 1s, Cu $2p_{3/2}$, O 1s, N 1s and S 2p) for TCNQ@HKUST-1(2) at 5 cycles on $Au^{TS}$.

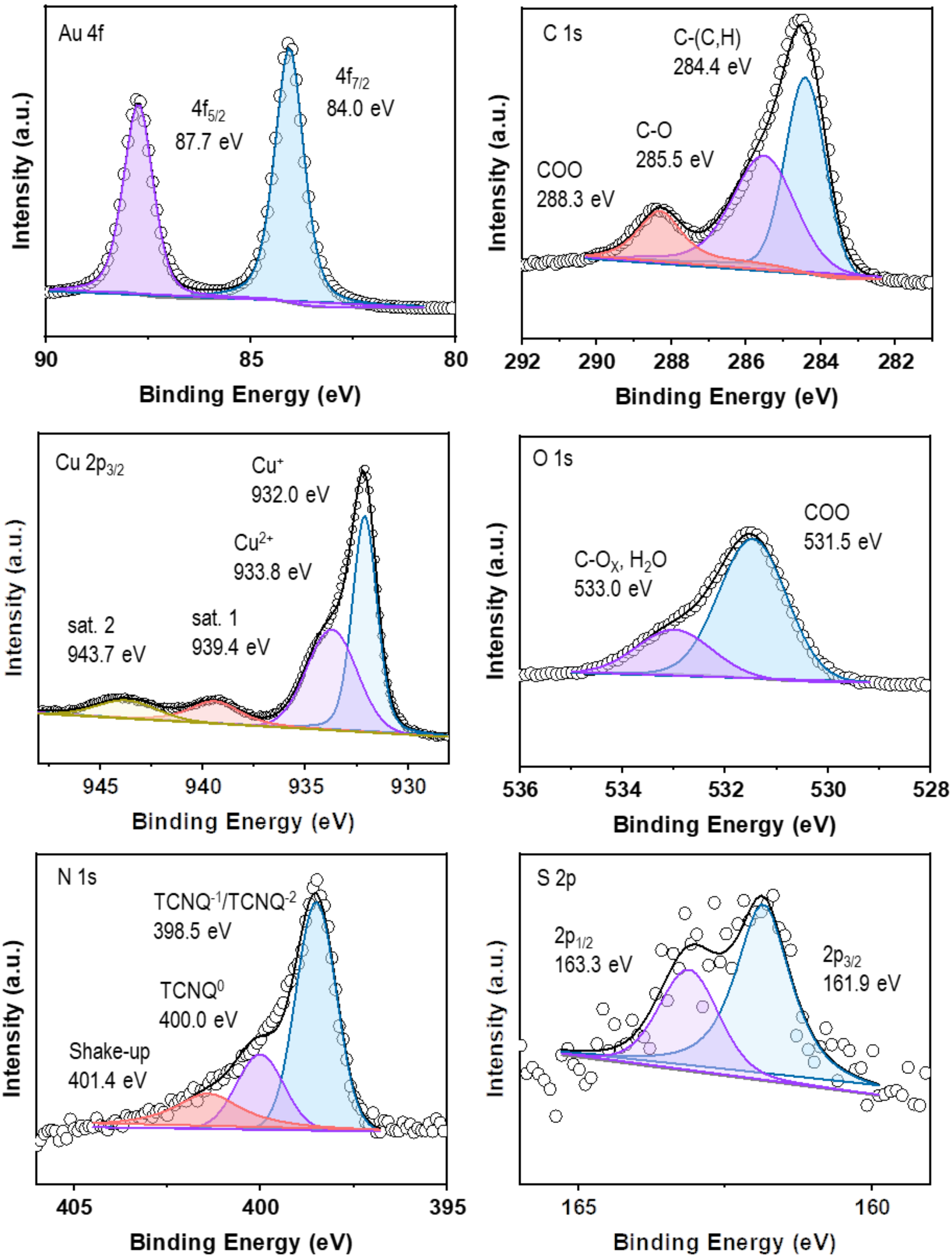

**Figure S27.** XPS high resolution spectra (Au 4f, C 1s, Cu $2p_{3/2}$, O 1s, N 1s and S 2p) for TCNQ@HKUST-1(2) at 6 cycles on $Au^{TS}$.

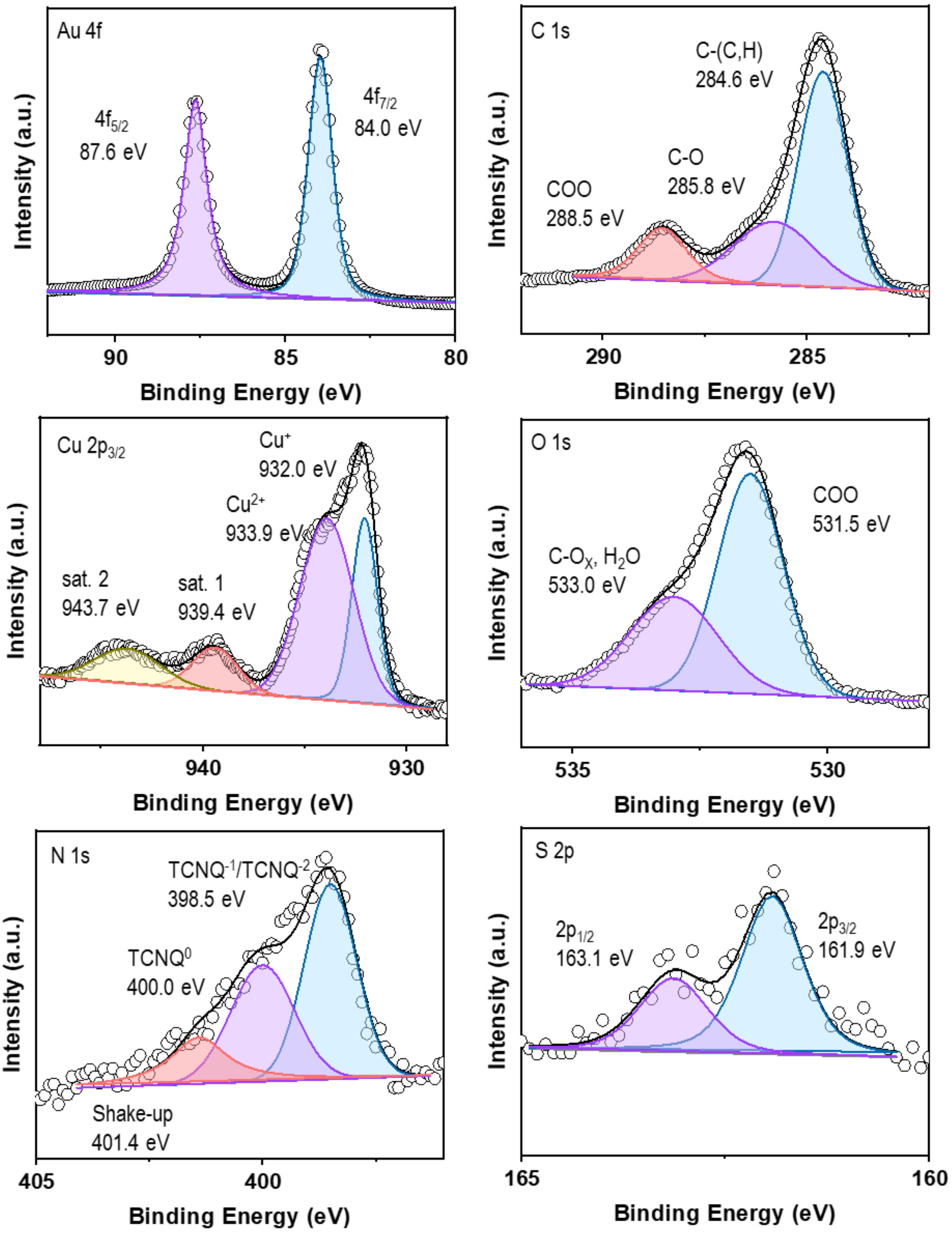

**Figure S28.** XPS high resolution spectra (Au 4f, C 1s, Cu $2p_{3/2}$, O 1s, N 1s and S 2p) for TCNQ@HKUST-1(2) at 7 cycles on $Au^{TS}$.

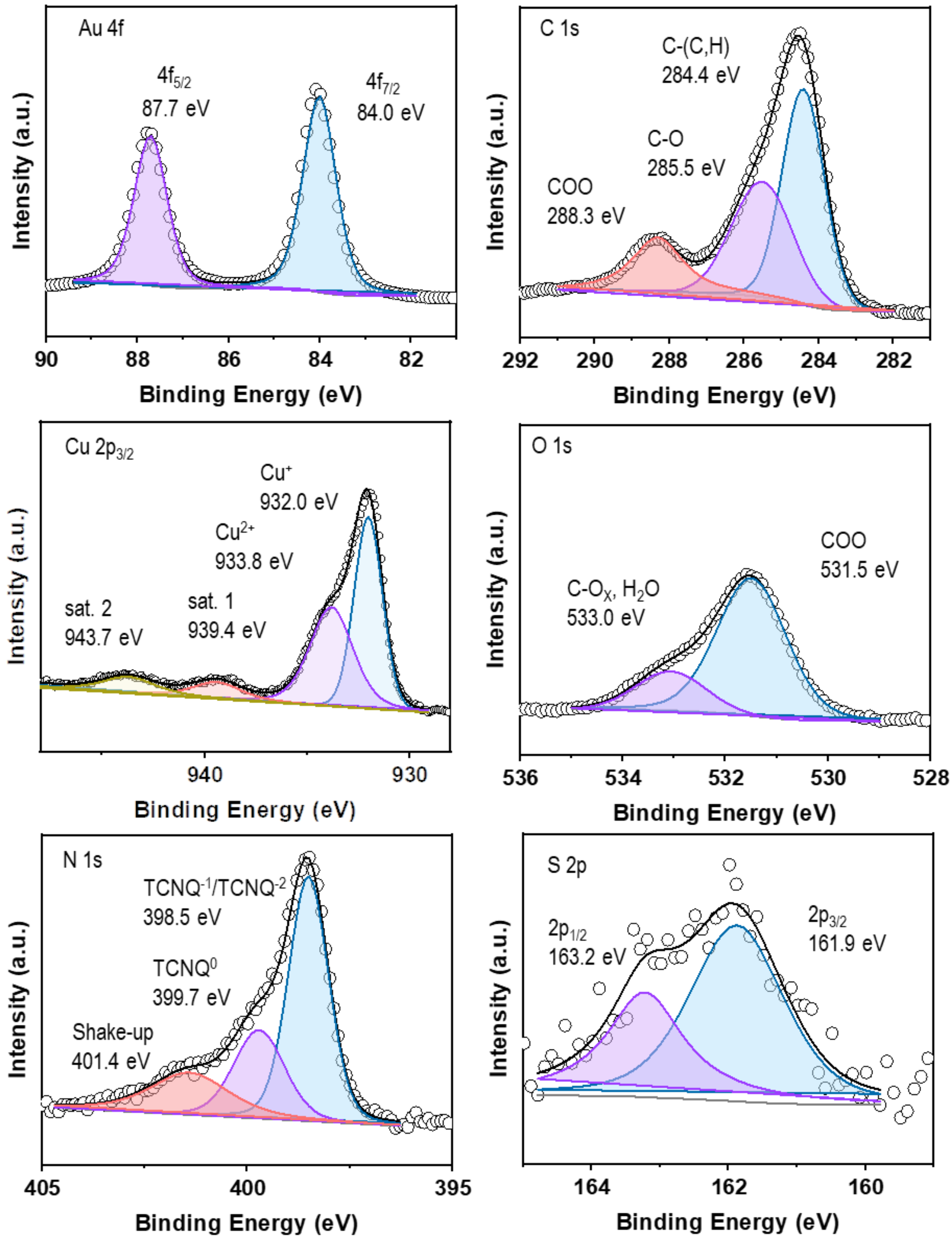

**Figure S29.** Histograms of current density measured on $Au^{TS}$/HKUST-1(2)//$Ga_2O_3$/EGaIn junctions at +0.5V. $N_{junction}$ is the number of junctions measured, $N_{traces}$ is the number of *J*-*V* traces, $\log J((+0.5\ V))_{mean}$ is mean, $\sigma_{\log J((+0.5\ V))}$ is the standard deviation value determined by fitting a Gaussian curve to the histogram.

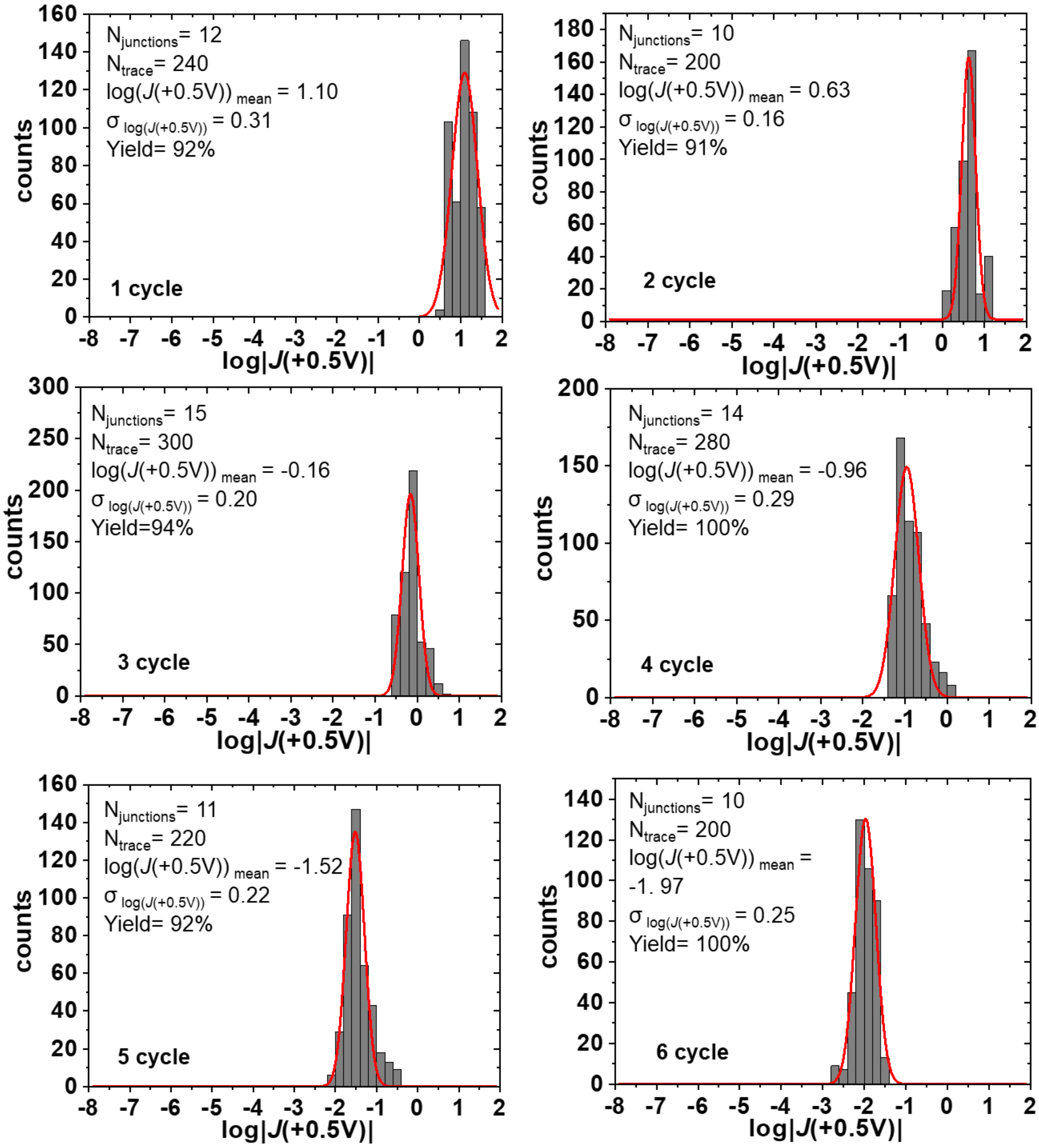

**Figure S30.** Histograms of current density measured on $Au^{TS}$/Fc@HKUST-1(2)//$Ga_2O_3$/EGaIn junctions at +0.5V.

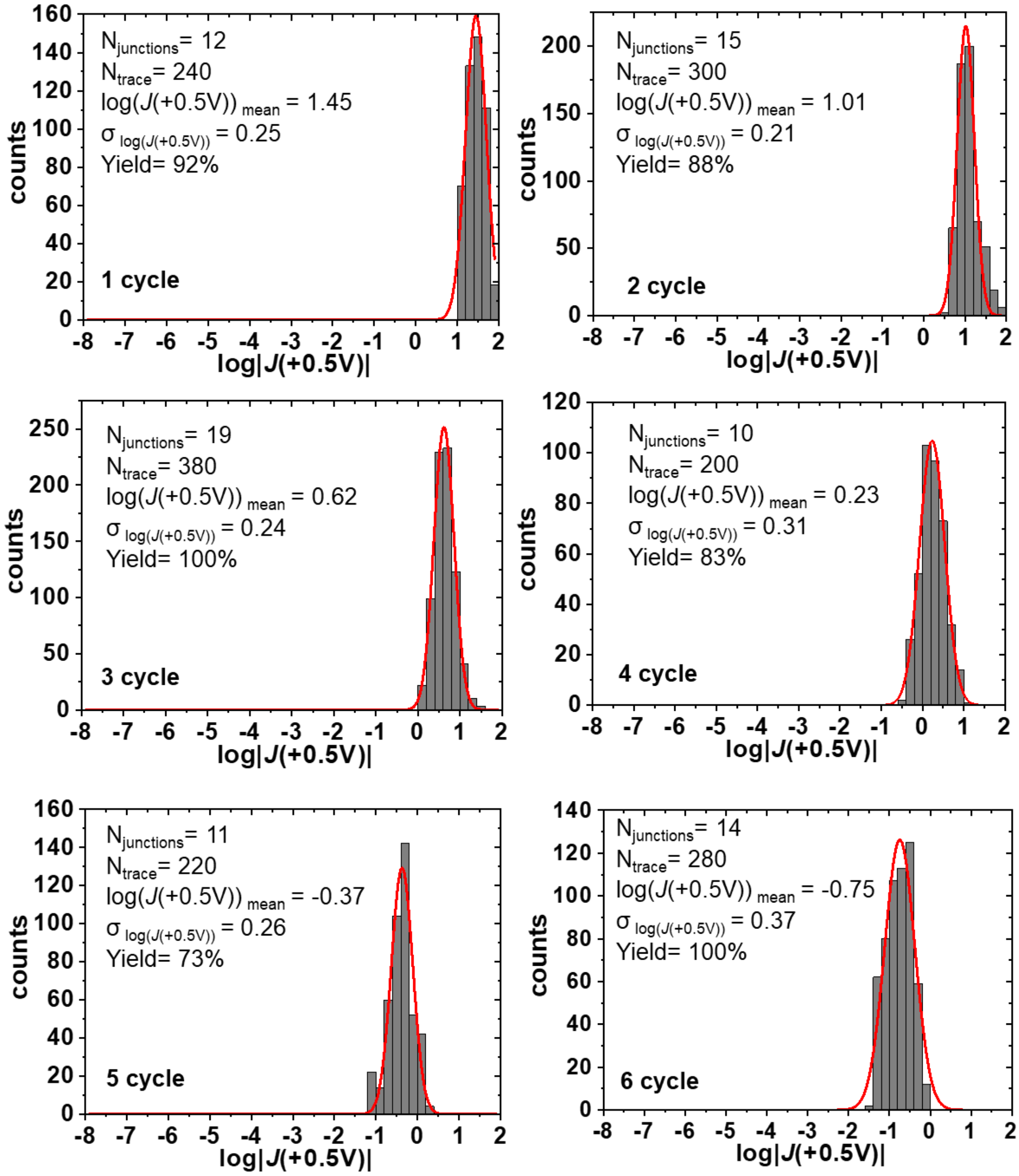

**Figure S31.** Histograms of current density measured on $Au^{TS}$/TCNQ@HKUST-1(2)//$Ga_2O_3$/EGaIn junctions at +0.5V.

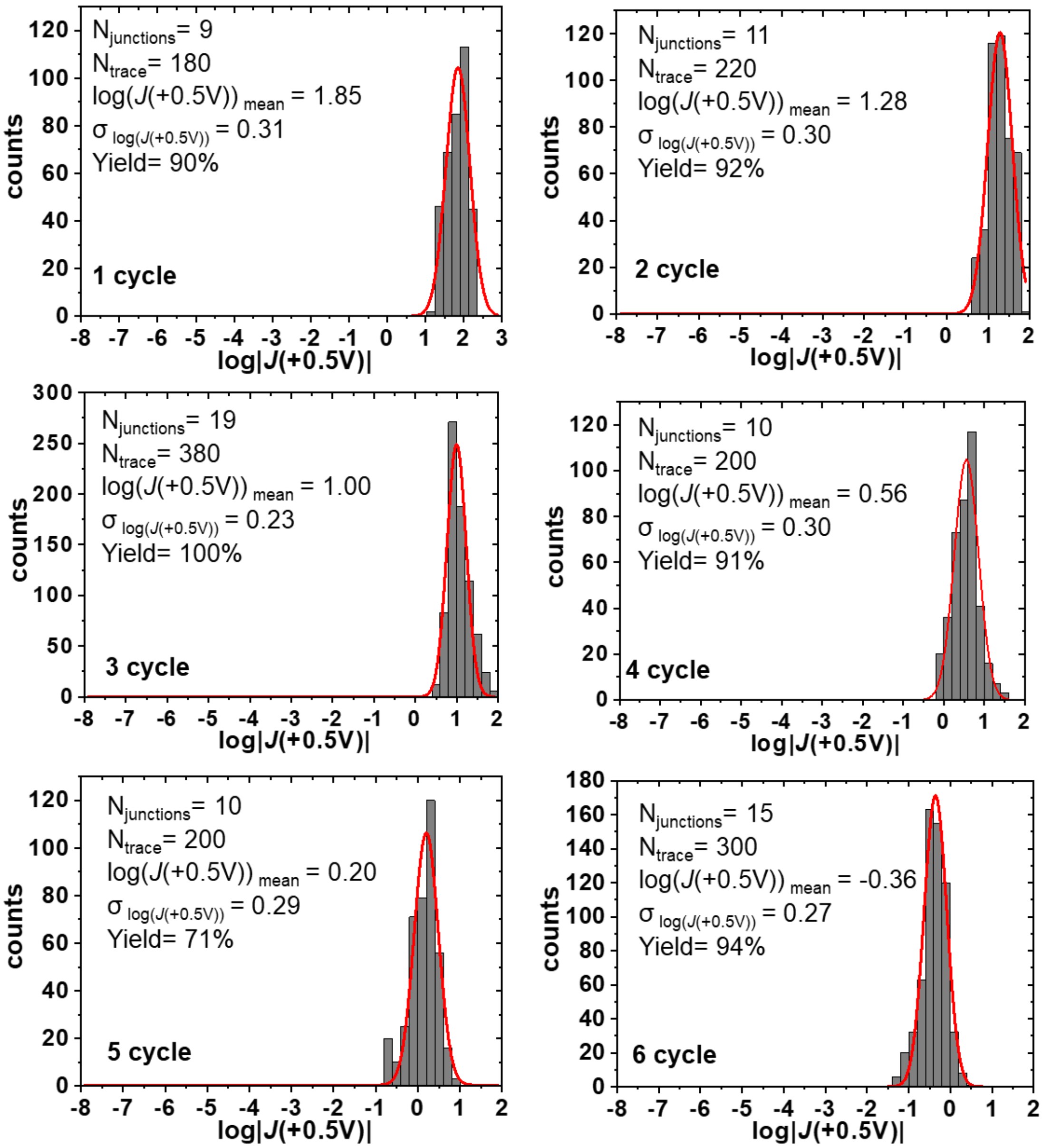

**Figure S32.** Histograms of current density measured on Au$^{TS}$/ HKUST-1(10)//$Ga_2O_3$/EGaIn junctions at +0.5V.

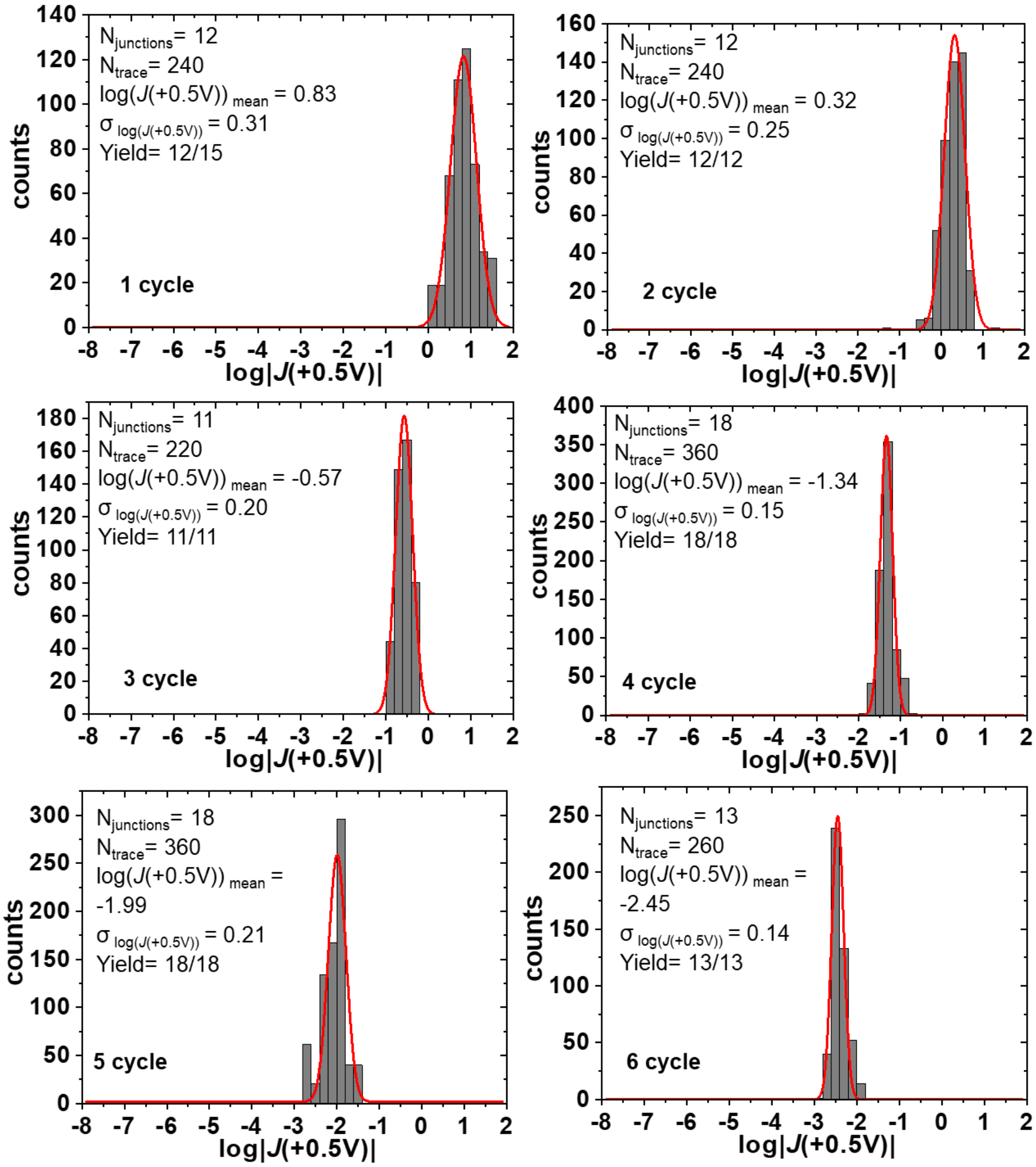

**Figure S33.** Histograms of current density measured on $Au^{TS}/SC_nCOOH(n = 2,10)//Ga_2O_3/EGaIn$ junctions at +0.5V.

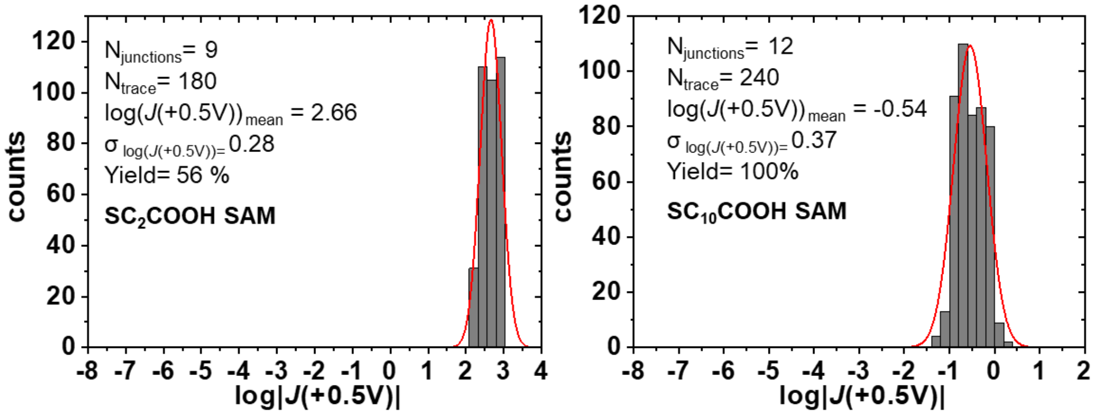

**Figure S34.** Thermopower analysis

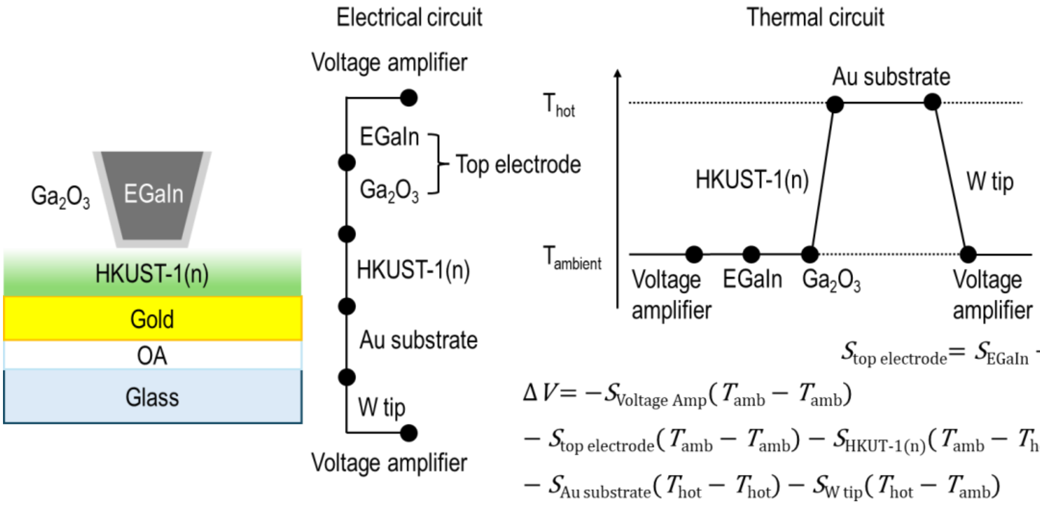


$$S_{\text{top electrode}} = S_{\text{EGaIn}} + S_{\text{Ga2O3}}$$

$$\Delta V = -S_{\text{Voltage Amp}}(T_{\text{amb}} - T_{\text{amb}})$$
$$- S_{\text{top electrode}}(T_{\text{amb}} - T_{\text{amb}}) - S_{\text{HKUT-1(n)}}(T_{\text{amb}} - T_{\text{hot}})$$
$$- S_{\text{Au substrate}}(T_{\text{hot}} - T_{\text{hot}}) - S_{\text{W tip}}(T_{\text{hot}} - T_{\text{amb}})$$
$$- S_{\text{Voltage Amp}}(T_{\text{amb}} - T_{\text{amb}}) = -(S_{\text{HKUST-1(n)}} - S_{\text{W tip}})(T_{\text{amb}} - T_{\text{hot}})$$
$$= -(S_{\text{HKUST-1(n)}} - S_{\text{W tip}})\,\Delta T$$

**Figure S35.** Histograms of thermovoltage measured on Au$^{TS}$/HKUST-1(2)//$Ga_2O_3$/EGaIn junctions.

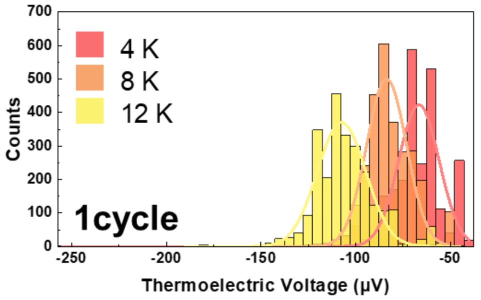


| ΔT (K) | 4 | 8 | 12 |
|---|---|---|---|
| Data | 2471 | 2727 | 2487 |
| Junctions | 24 | 26 | 24 |
| Mean | -66.5 | -83.6 | -107.4 |
| Sigma | 10.7 | 10.5 | 12.8 |
| Yield (%) | 71 | 93 | 69 |
| Seebeck coefficient | | 6.1 ± 0.5 | |

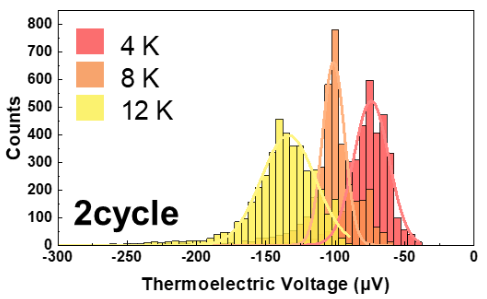


| ΔT (K) | 4 | 8 | 12 |
|---|---|---|---|
| Data | 3408 | 3484 | 4041 |
| Junctions | 33 | 33 | 40 |
| Mean | -74.1 | -101.3 | -133.8 |
| Sigma | 12.7 | 7.9 | 19.5 |
| Yield (%) | 85 | 79 | 89 |
| Seebeck coefficient | | 8.3 ± 0.5 | |

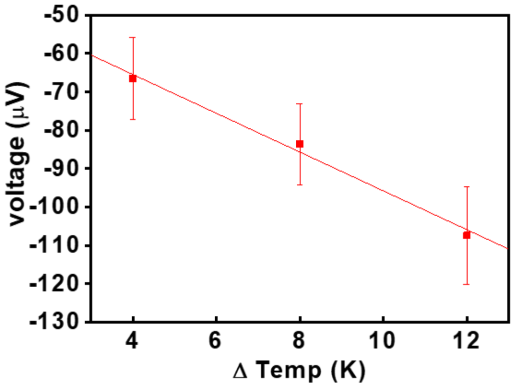


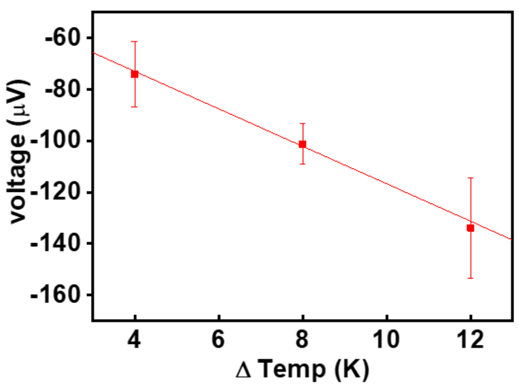

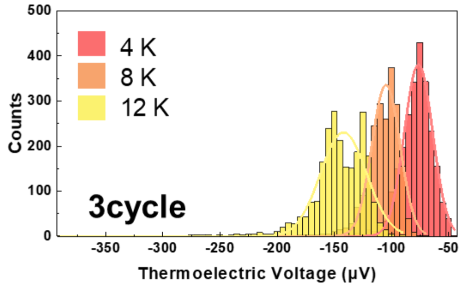


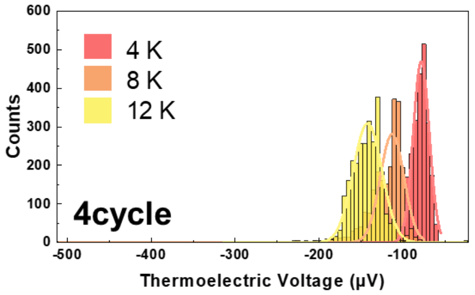


| ΔT (K) | 4 | 8 | 12 |
|---|---|---|---|
| **Data** | 2420 | 2090 | 2334 |
| **Junctions** | 23 | 20 | 22 |
| **Mean** | -76.5 | -104.9 | -142.9 |
| **Sigma** | 12.4 | 12.3 | 20.1 |
| **Yield (%)** | 59 | 71 | 76 |
| **Seebeck coefficient** | | 9.0 ± 0.7 | |

| ΔT (K) | 4 | 8 | 12 |
|---|---|---|---|
| **Data** | 2268 | 2180 | 2608 |
| **Junctions** | 22 | 21 | 25 |
| **Mean** | -78.3 | -113.0 | -142.2 |
| **Sigma** | 9.6 | 14.1 | 17.1 |
| **Yield (%)** | 85 | 100 | 89 |
| **Seebeck coefficient** | | 9.1 ± 0.4 | |

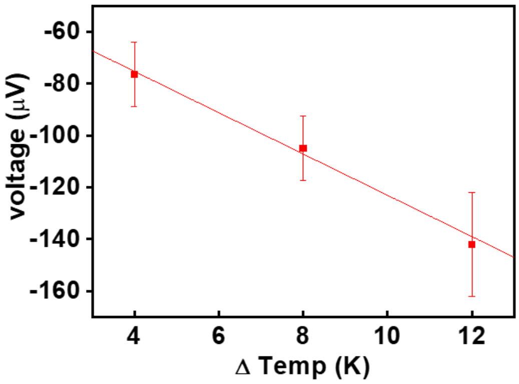


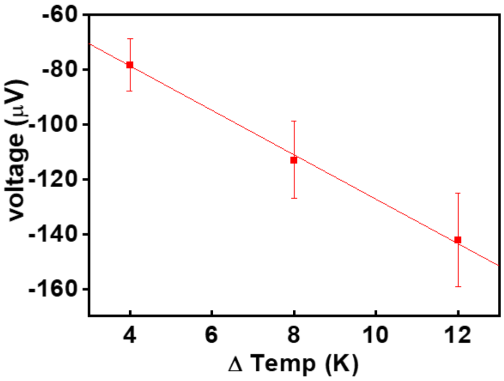

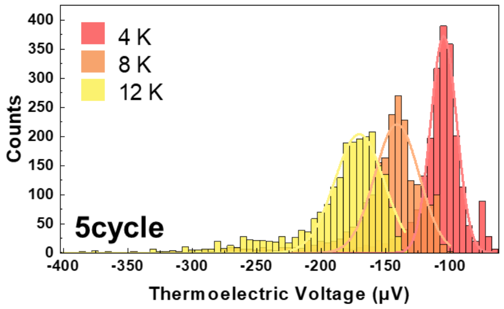


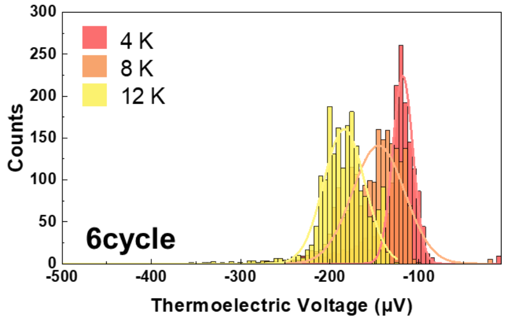


| ΔT (K) | 4 | 8 | 12 |
|---|---|---|---|
| **Data** | 2221 | 2123 | 2199 |
| **Junctions** | 21 | 20 | 21 |
| **Mean** | -105.0 | -141.2 | -170.4 |
| **Sigma** | 10.1 | 17.2 | 19.1 |
| **Yield (%)** | 78 | 87 | 75 |
| **Seebeck coefficient** | | 9.3 ± 0.4 | |

| ΔT (K) | 4 | 8 | 12 |
|---|---|---|---|
| **Data** | 1586 | 2076 | 1876 |
| **Junctions** | 15 | 20 | 18 |
| **Mean** | -118.2 | -145.3 | -183.8 |
| **Sigma** | 11.3 | 29.5 | 23.0 |
| **Yield (%)** | 88 | 95 | 67 |
| **Seebeck coefficient** | | 9.1 ± 0.5 | |

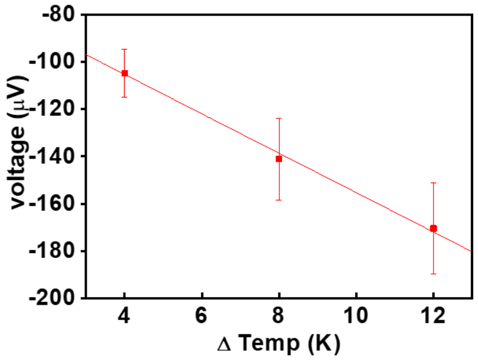


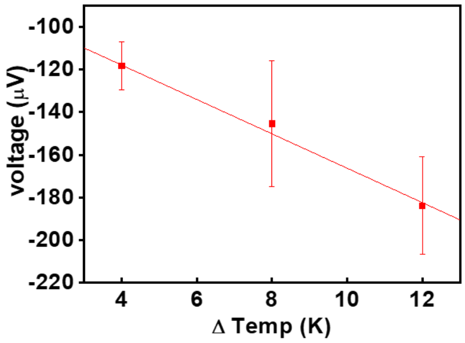

**Figure S36.** Histograms of thermovoltage measured on $Au^{TS}$/Fc@HKUST-1(2)//$Ga_2O_3$/EGaIn junctions.

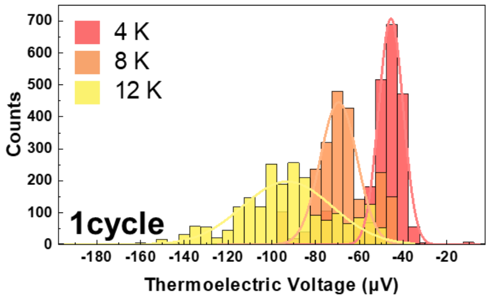


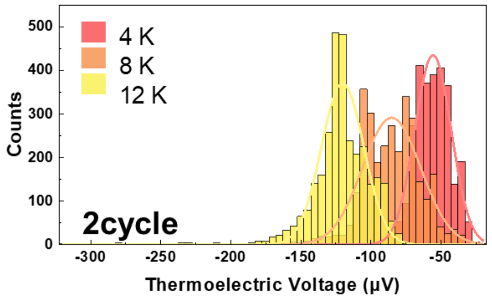


| ΔT (K) | 4 | 8 | 12 |
|---|---|---|---|
| **Data** | 2094 | 2211 | 2118 |
| **Junctions** | 20 | 21 | 20 |
| **Mean** | -45.6 | -69.5 | -92.9 |
| **Sigma** | 5.6 | 8.4 | 20.5 |
| **Yield (%)** | 91 | 88 | 83 |
| **Seebeck coefficient** | | 6.9 ± 0.0 | |

| ΔT (K) | 4 | 8 | 12 |
|---|---|---|---|
| **Data** | 2784 | 2988 | 3016 |
| **Junctions** | 27 | 29 | 29 |
| **Mean** | -55.7 | -85.2 | -120.2 |
| **Sigma** | 12.9 | 20.8 | 15.2 |
| **Yield (%)** | 84 | 74 | 85 |
| **Seebeck coefficient** | | 9.1 ± 0.3 | |

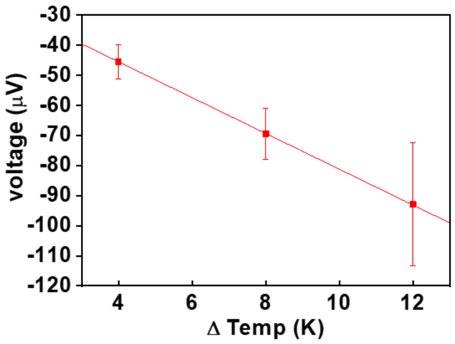


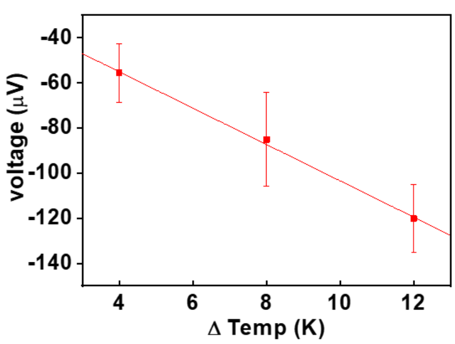

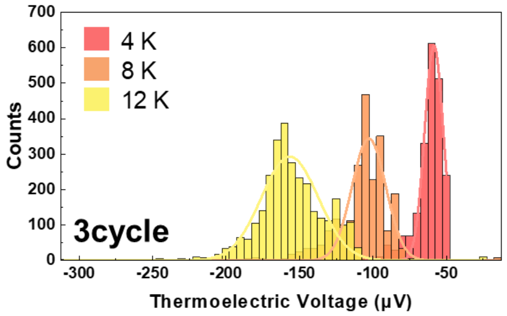


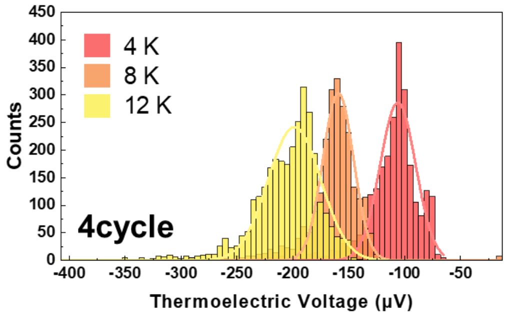


| ΔT (K) | 4 | 8 | 12 |
|---|---|---|---|
| **Data** | 2045 | 2219 | 2984 |
| **Junctions** | 20 | 21 | 27 |
| **Mean** | -58.6 | -102.8 | -156.6 |
| **Sigma** | 6.3 | 11.5 | 19.4 |
| **Yield (%)** | 83 | 84 | 84 |
| **Seebeck coefficient** | | 12.8 ± 0.7 | |

| ΔT (K) | 4 | 8 | 12 |
|---|---|---|---|
| **Data** | 2507 | 2327 | 2985 |
| **Junctions** | 24 | 23 | 29 |
| **Mean** | -106.1 | -159.4 | -198.6 |
| **Sigma** | 15.9 | 13.7 | 23.5 |
| **Yield (%)** | 53 | 92 | 94 |
| **Seebeck coefficient** | | 12.9 ± 1.2 | |

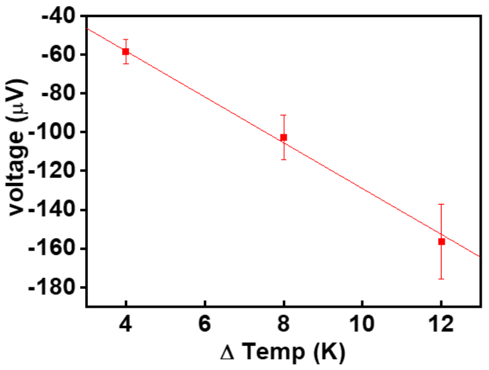


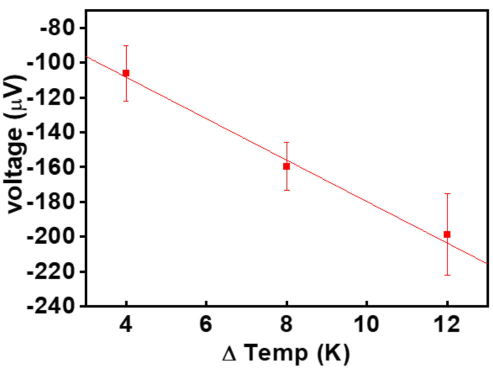

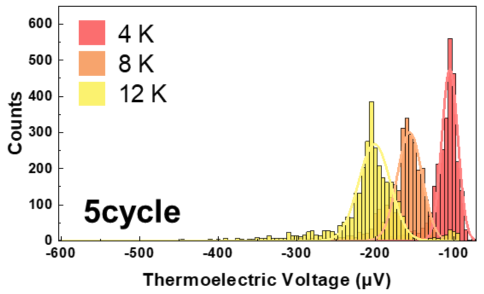


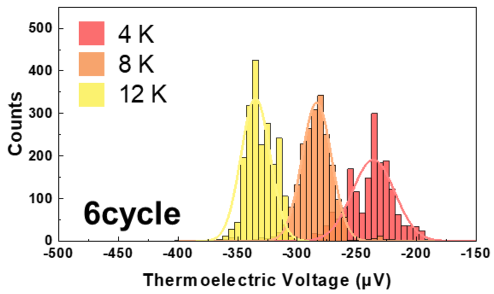


| ΔT (K) | 4 | 8 | 12 |
|---|---|---|---|
| **Data** | 2696 | 2672 | 3172 |
| **Junctions** | 26 | 25 | 30 |
| **Mean** | -105.6 | -156.6 | -201.4 |
| **Sigma** | 10.1 | 15.7 | 20.2 |
| **Yield (%)** | 90 | 89 | 94 |
| **Seebeck coefficient** | 13.1 ± 0.4 | | |

| ΔT (K) | 4 | 8 | 12 |
|---|---|---|---|
| **Data** | 1773 | 2074 | 2072 |
| **Junctions** | 18 | 20 | 20 |
| **Mean** | -236.0 | -283.6 | -335.0 |
| **Sigma** | 17.9 | 12.3 | 12.2 |
| **Yield (%)** | 89 | 95 | 95 |
| **Seebeck coefficient** | 13.5 ± 0.3 | | |

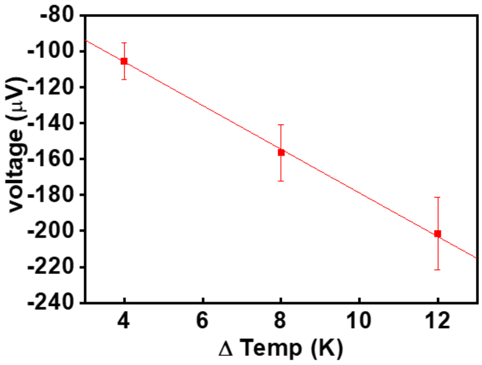


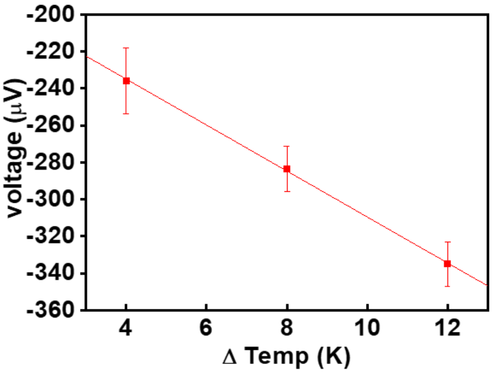

**Figure S37.** Histograms of thermovoltage measured on $Au^{TS}$/TCNQ@HKUST-1(2)//$Ga_2O_3$/EGaIn junctions.

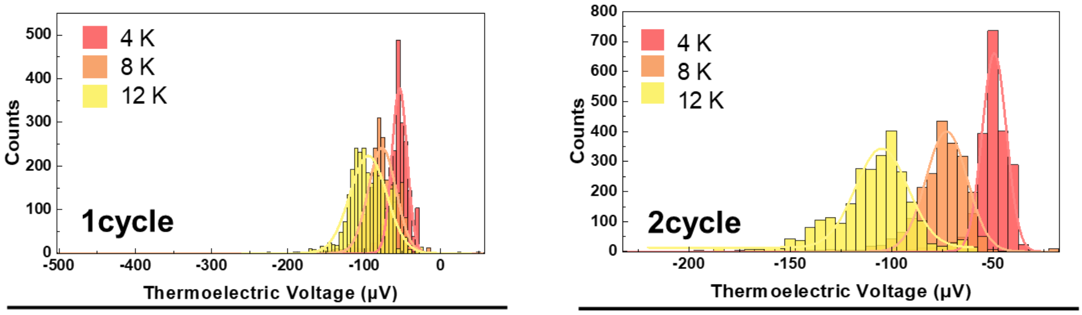


| ΔT (K) | 4 | 8 | 12 |
|---|---|---|---|
| Data | 2091 | 2262 | 2690 |
| Junctions | 20 | 22 | 28 |
| Mean | -53.8 | -76.5 | -94.7 |
| Sigma | 9.9 | 18.2 | 24.1 |
| Yield (%) | 95 | 96 | 70 |
| Seebeck coefficient | 6.3 ± 0.3 | | |

| ΔT (K) | 4 | 8 | 12 |
|---|---|---|---|
| Data | 2107 | 2118 | 2590 |
| Junctions | 20 | 20 | 25 |
| Mean | -49.4 | -72.9 | -104.9 |
| Sigma | 6.0 | 10.2 | 12.9 |
| Yield (%) | 100 | 91 | 83 |
| Seebeck coefficient | 7.7± 0.6 | | |

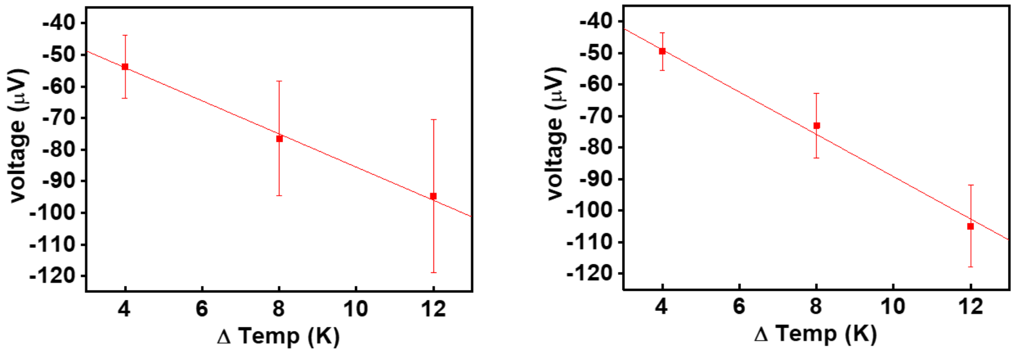

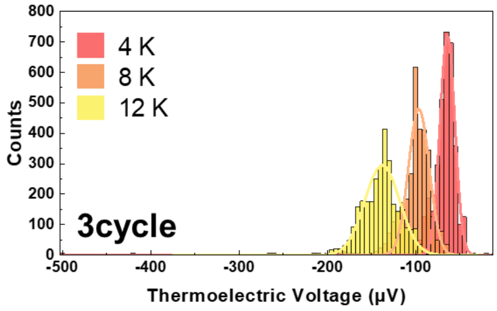


| ΔT (K) | 4 | 8 | 12 |
|---|---|---|---|
| **Data** | 2420 | 3081 | 2979 |
| **Junctions** | 23 | 29 | 28 |
| **Mean** | -76.5 | -96.5 | -137.9 |
| **Sigma** | 12.4 | 11.7 | 19.7 |
| **Yield (%)** | 82 | 94 | 82 |
| **Seebeck coefficient** | | 9.9 ± 0.7 | |

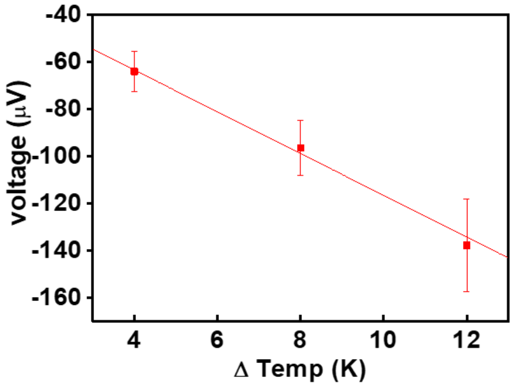


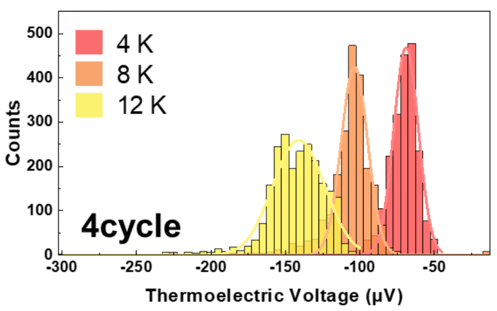


| ΔT (K) | 4 | 8 | 12 |
|---|---|---|---|
| **Data** | 2043 | 2129 | 2243 |
| **Junctions** | 20 | 21 | 21 |
| **Mean** | -69.1 | -103.7 | -141.1 |
| **Sigma** | 8.3 | 8.9 | 17.3 |
| **Yield (%)** | 100 | 95 | 100 |
| **Seebeck coefficient** | | 9.9 ± 0.2 | |

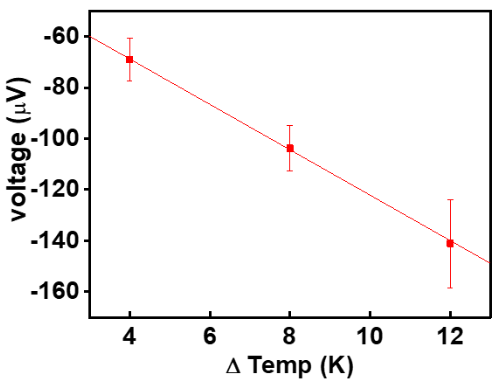

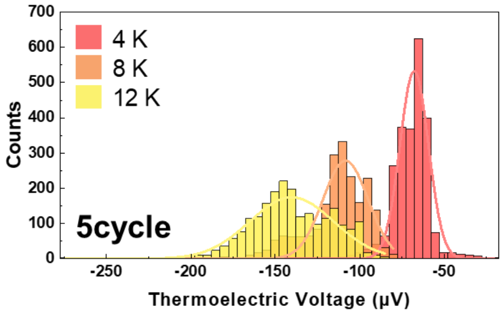


| ΔT (K) | 4 | 8 | 12 |
|---|---|---|---|
| **Data** | 2330 | 2022 | 2130 |
| **Junctions** | 23 | 20 | 21 |
| **Mean** | -67.7 | -108.4 | -140.2 |
| **Sigma** | 8.5 | 13.3 | 25.0 |
| **Yield (%)** | 82 | 100 | 91 |
| **Seebeck coefficient** | | 10.5 ± 0.6 | |

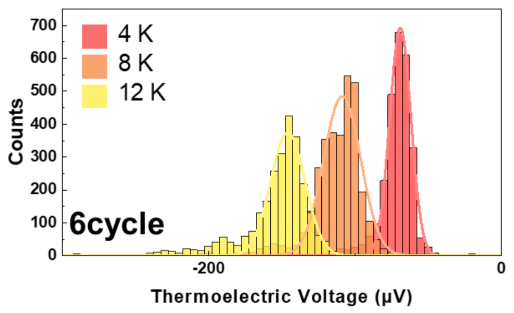


| ΔT (K) | 4 | 8 | 12 |
|---|---|---|---|
| **Data** | 2693 | 3227 | 2611 |
| **Junctions** | 26 | 31 | 26 |
| **Mean** | -69.1 | -108.9 | -145.9 |
| **Sigma** | 7.3 | 12.7 | 11.7 |
| **Yield (%)** | 100 | 89 | 74 |
| **Seebeck coefficient** | | 10.6± 0.2 | |

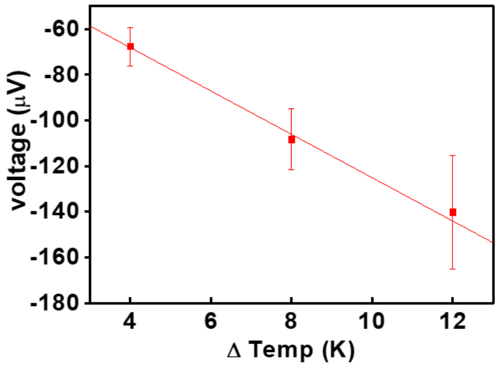


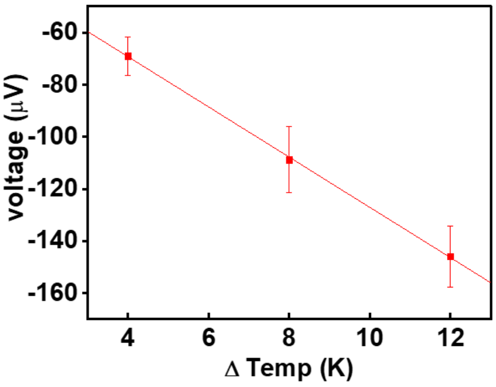

**Figure S38.** Histograms of thermovoltage measured on Au[TS]/HKUST-1(10)//$Ga_2O_3$/EGaIn junctions.

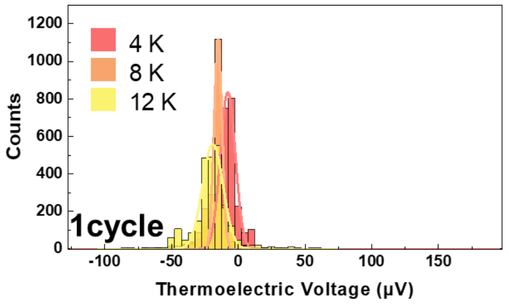


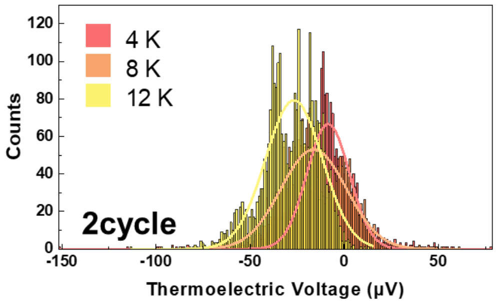


| ΔT (K) | 4 | 8 | 12 |
|---|---|---|---|
| **Data** | 2793 | 2085 | 2473 |
| **Junctions** | 27 | 20 | 24 |
| **Mean** | -7.9 | -15.2 | -19.1 |
| **Sigma** | 5.6 | 3.0 | 7.7 |
| **Yield (%)** | 93 | 100 | 89 |
| **Seebeck coefficient** | | 2.5 ± 0.3 | |

| ΔT (K) | 4 | 8 | 12 |
|---|---|---|---|
| **Data** | 2142 | 2268 | 2970 |
| **Junctions** | 23 | 22 | 29 |
| **Mean** | -8.6 | -16.3 | -26.7 |
| **Sigma** | 11.3 | 17.4 | 15.1 |
| **Yield (%)** | 96 | 88 | 100 |
| **Seebeck coefficient** | | 3.3 ± 0.2 | |

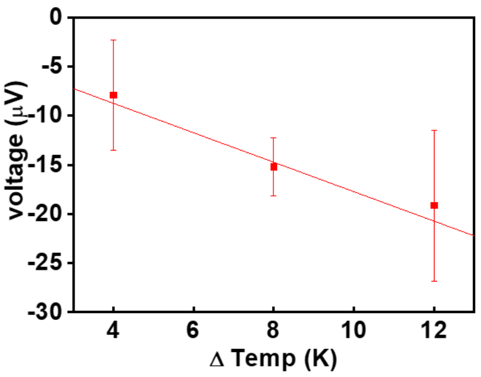


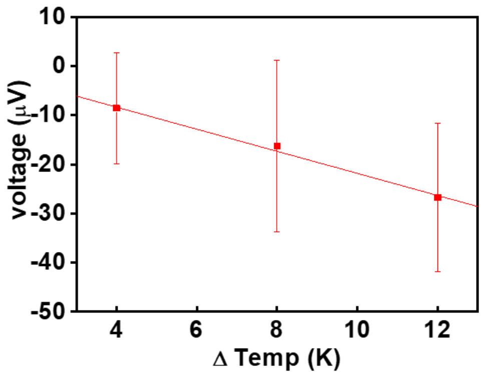

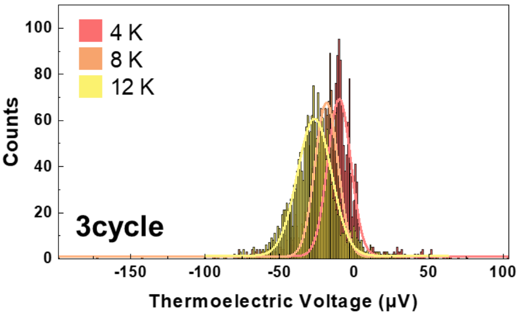


| ΔT (K) | 4 | 8 | 12 |
|---|---|---|---|
| **Data** | 1529 | 1521 | 1924 |
| **Junctions** | 15 | 15 | 19 |
| **Mean** | -10.1 | -18.6 | -26.7 |
| **Sigma** | 7.8 | 7.7 | 11.4 |
| **Yield (%)** | 88 | 100 | 100 |
| **Seebeck coefficient** | | 3.1 ± 0.0 | |

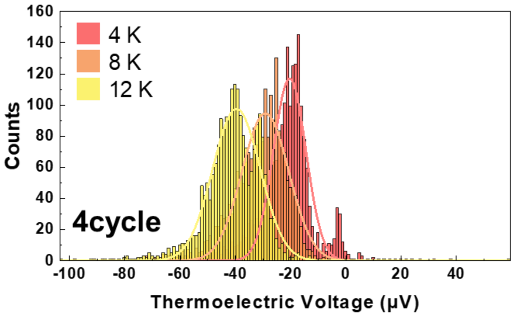


| ΔT (K) | 4 | 8 | 12 |
|---|---|---|---|
| **Data** | 2022 | 2090 | 2140 |
| **Junctions** | 20 | 20 | 21 |
| **Mean** | -20.3 | -29.5 | -39.5 |
| **Sigma** | 5.9 | 8.5 | 8.6 |
| **Yield (%)** | 95 | 83 | 95 |
| **Seebeck coefficient** | | 3.4 ± 0.1 | |

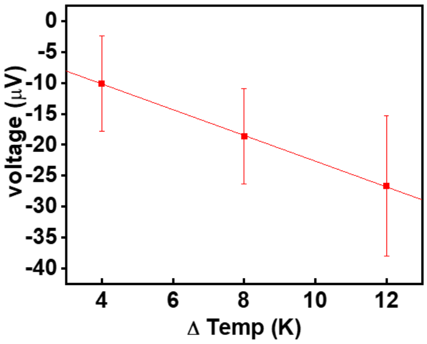


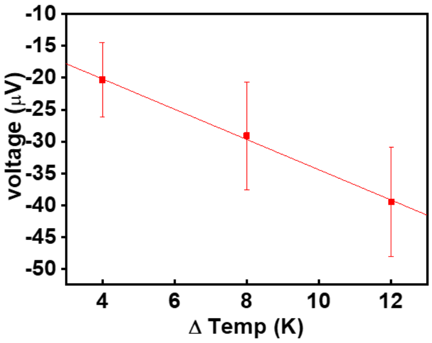

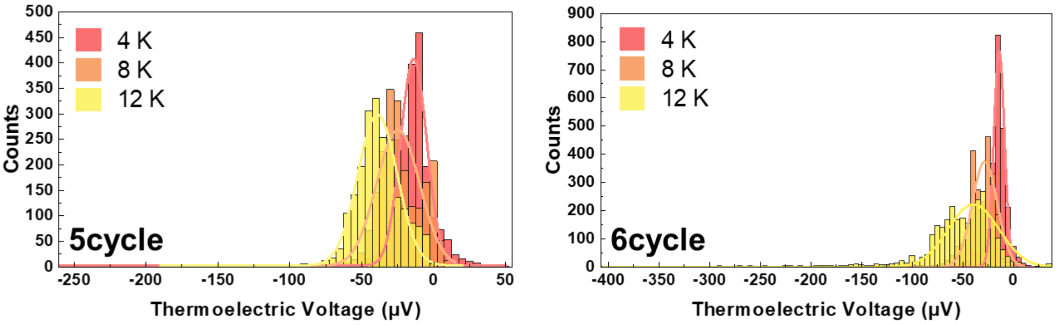


| ΔT (K) | 4 | 8 | 12 |
|---|---|---|---|
| **Data** | 2020 | 2035 | 2139 |
| **Junctions** | 20 | 20 | 21 |
| **Mean** | -14.0 | -24.8 | -39.1 |
| **Sigma** | 9.0 | 15.0 | 13.6 |
| **Yield (%)** | 77 | 77 | 84 |
| **Seebeck coefficient** | | 4.1 ± 0.2 | |

| ΔT (K) | 4 | 8 | 12 |
|---|---|---|---|
| **Data** | 2349 | 2334 | 2926 |
| **Junctions** | 23 | 22 | 28 |
| **Mean** | -14.3 | -28.5 | -39.5 |
| **Sigma** | 5.3 | 12.4 | 25.0 |
| **Yield (%)** | 96 | 85 | 82 |
| **Seebeck coefficient** | | 4.3 ± 0.2 | |

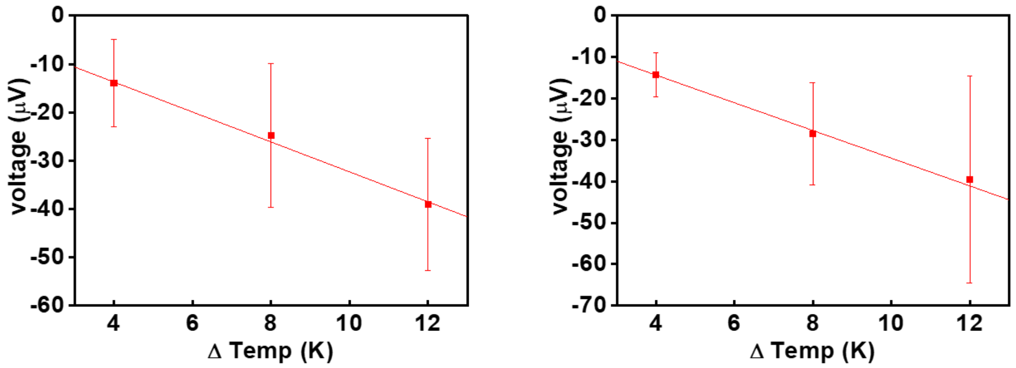

**Figure S39.** Histograms of thermovoltage measured on $Au^{TS}$/ $SC_nCOOH(n=2,10)//Ga_2O_3$/EGaIn junctions.

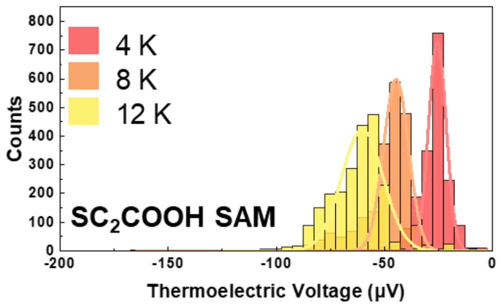


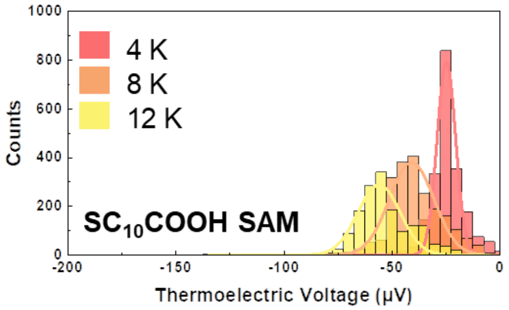


| ΔT (K) | 4 | 8 | 12 |
|---|---|---|---|
| **Data** | 1756 | 2063 | 2175 |
| **Junctions** | 17 | 20 | 21 |
| **Mean** | -25.6 | -44.7 | -60.3 |
| **Sigma** | 4.1 | 5.8 | 9.7 |
| **Yield (%)** | 63% | 77% | 72% |
| **Seebeck coefficient** | 5.5 ± 0.3 | | |

| ΔT (K) | 4 | 8 | 12 |
|---|---|---|---|
| **Data** | 2066 | 2070 | 1550 |
| **Junctions** | 20 | 20 | 15 |
| **Mean** | -24.9 | -40.9 | -56.0 |
| **Sigma** | 4.3 | 10.3 | 9.8 |
| **Yield (%)** | 95% | 71% | 65% |
| **Seebeck coefficient** | 4.9 ± 0.1 | | |

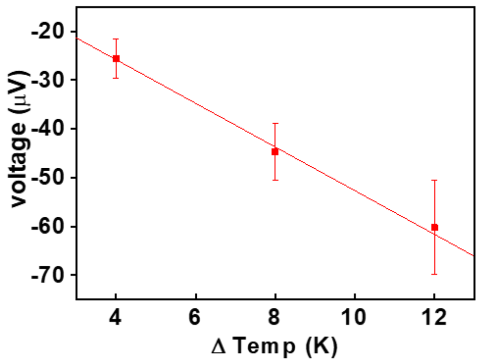


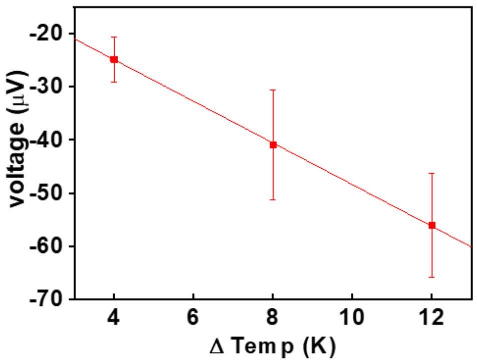

**Figure S40.** UPS spectra of (a) Au$^{TS}$ and (b) HKUST-1(2).

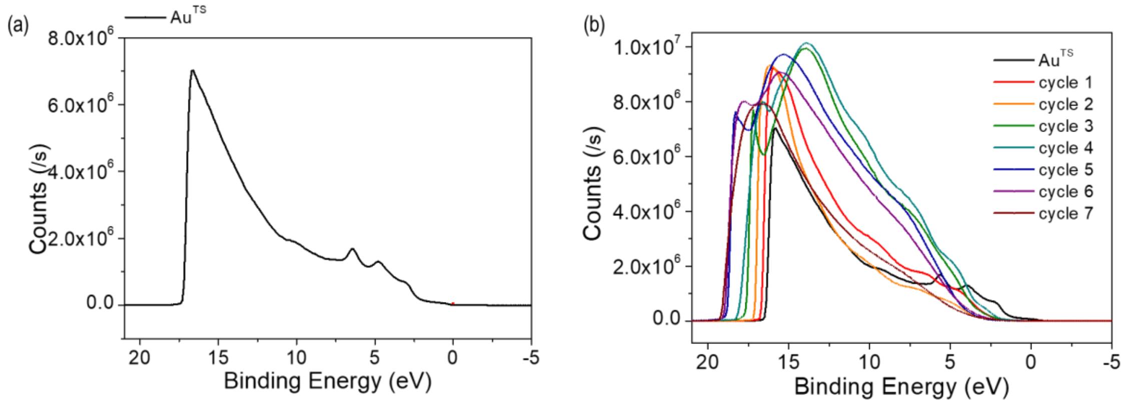


**Figure S41.** UPS spectra of Fc@HKUST-1(2) (a) in the cut-off region and (b) in the on-set region.

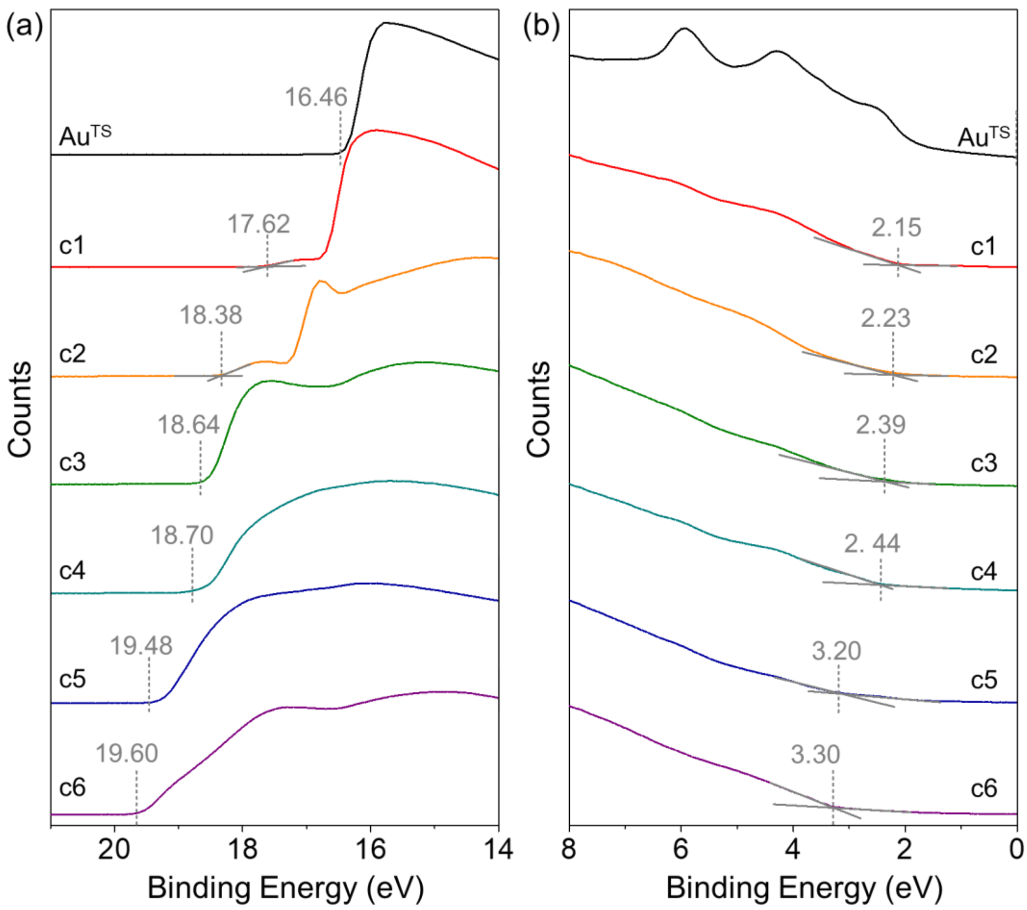

**Figure S42.** UPS spectra of TCNQ@HKUST-1(2) (a) in the cut-off region and (b) in the on-set region.

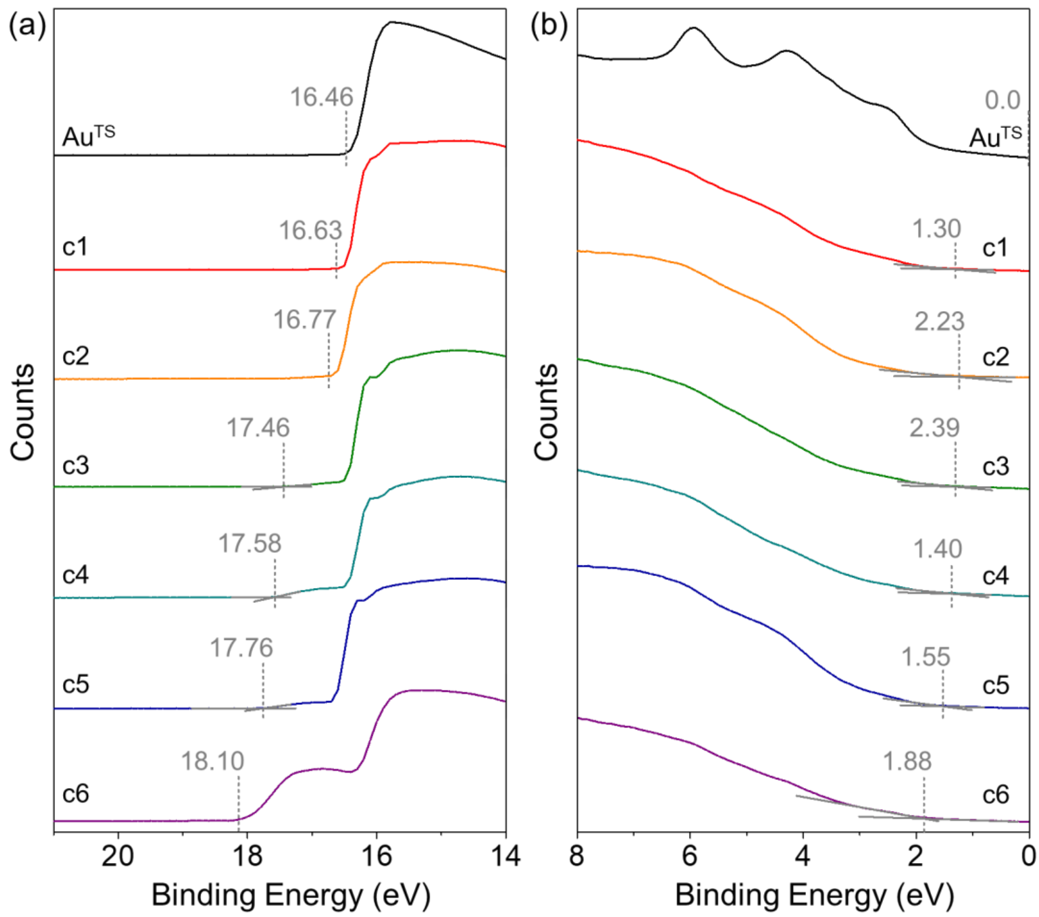

**Table S1.** Summary of current density measurements for $Au^{TS}$/HKUST-1(2)//$Ga_2O_3$/EGaIn junctions at +0.5V.

| Cycle | $N_{junctions}$ | $N_{traces}$ | $Log(J(+0.5V))_{mean}$ | $\sigma_{Log(J(+0.5V))}$ | Yield(%) |
|---|---|---|---|---|---|
| 1 | 12 | 240 | 1.10 | 0.31 | 92 |
| 2 | 10 | 200 | 0.63 | 0.16 | 91 |
| 3 | 15 | 300 | -0.16 | 0.20 | 94 |
| 4 | 14 | 280 | -0.96 | 0.29 | 100 |
| 5 | 11 | 220 | -1.52 | 0.22 | 92 |
| 6 | 10 | 200 | -1.97 | 0.25 | 100 |

**Table S2.** Summary of current density measurements for $Au^{TS}$/Fc@HKUST-1(2)//$Ga_2O_3$/EGaIn junctions at +0.5V.

| Cycle | $N_{junctions}$ | $N_{traces}$ | $Log(J(+0.5V))_{mean}$ | $\sigma_{Log(J(+0.5V))}$ | Yield(%) |
|---|---|---|---|---|---|
| 1 | 12 | 240 | 1.45 | 0.25 | 92 |
| 2 | 15 | 300 | 1.01 | 0.21 | 88 |
| 3 | 19 | 380 | 0.62 | 0.24 | 100 |
| 4 | 10 | 200 | 0.23 | 0.31 | 83 |
| 5 | 11 | 220 | -0.37 | 0.26 | 73 |
| 6 | 14 | 280 | -0.75 | 0.37 | 100 |

**Table S3.** Summary of current density measurements for Au$^{TS}$/TCNQ@HKUST-1(2)//$Ga_2O_3$/EGaIn junctions at +0.5V.

| Cycle | $N_{junctions}$ | $N_{traces}$ | $Log(J(+0.5V))_{mean}$ | $\sigma_{Log(J(+0.5V))}$ | Yield(%) |
|---|---|---|---|---|---|
| 1 | 9 | 180 | 1.85 | 0.31 | 90 |
| 2 | 11 | 220 | 1.28 | 0.30 | 92 |
| 3 | 19 | 380 | 1.00 | 0.23 | 100 |
| 4 | 10 | 200 | 0.56 | 0.30 | 91 |
| 5 | 10 | 200 | 0.20 | 0.29 | 71 |
| 6 | 15 | 300 | -0.36 | 0.27 | 94 |

**Table S4.** Summary of current density measurements for Au$^{TS}$/HKUST-1(10)//$Ga_2O_3$/EGaIn junctions at +0.5V.

| Cycle | $N_{junctions}$ | $N_{traces}$ | $Log(J(+0.5V))_{mean}$ | $\sigma_{Log(J(+0.5V))}$ | Yield(%) |
|---|---|---|---|---|---|
| 1 | 12 | 240 | 0.83 | 0.31 | 80 |
| 2 | 12 | 240 | 0.32 | 0.25 | 100 |
| 3 | 11 | 220 | -0.57 | 0.20 | 100 |
| 4 | 18 | 360 | -1.34 | 0.15 | 100 |
| 5 | 18 | 390 | -1.99 | 0.21 | 100 |
| 6 | 13 | 360 | -2.45 | 0.14 | 100 |

**Table S5.** Summary of current density measurements for $Au^{TS}/SC_nCOOH$($n$ = 2,10)//$Ga_2O_3$/EGaIn junctions at +0.5V.

| $n$ | $N_{junctions}$ | $N_{traces}$ | $Log(J(+0.5V))_{mean}$ | $\sigma_{Log(J(+0.5V))}$ | Yield(%) |
|---|---|---|---|---|---|
| 2 | 9 | 180 | 2.66 | 0.37 | 56 |
| 10 | 12 | 240 | -0.54 | 0.28 | 100 |

**Table S6.** Summary of thermoelectric voltage measurements for $Au^{TS}$/HKUST-1(2)//$Ga_2O_3$/EGaIn junctions.

| Cycle | $\Delta T$ (K) | $N_{junctions}$ | counts | $\Delta V_{mean} \pm \sigma_{\Delta V}$ | $S_{HKUST-1}$ (μV/K) | Yield (%) |
|---|---|---|---|---|---|---|
| | 4 | 24 | 2471 | -66.5 ± 10.7 | | 71 |
| 1 | 8 | 26 | 2727 | -83.6 ± 10.5 | 6.1 ± 0.5 | 93 |
| | 12 | 24 | 2487 | -107.4 ± 12.8 | | 69 |
| | 4 | 33 | 3408 | -74.1 ± 12.7 | | 85 |
| 2 | 8 | 33 | 3484 | -101.3 ± 7.9 | 8.3 ± 0.5 | 79 |
| | 12 | 40 | 4041 | -133.8±19.5 | | 89 |
| | 4 | 23 | 2420 | -76.5 ± 12.4 | | 59 |
| 3 | 8 | 20 | 2090 | -104.9 ± 12.3 | 9.0 ± 0.7 | 71 |
| | 12 | 22 | 2334 | -142.9 ± 20.1 | | 76 |
| | 4 | 22 | 2268 | -78.3 ± 9.6 | | 85 |
| 4 | 8 | 21 | 2180 | -113.0 ± 14.1 | 9.1 ± 0.4 | 100 |
| | 12 | 25 | 2608 | -142.0 ± 17.1 | | 89 |
| | 4 | 21 | 2221 | -105.0 ± 10.1 | | 78 |
| 5 | 8 | 20 | 2123 | -141.2 ± 17.2 | 9.3 ± 0.4 | 87 |
| | 12 | 21 | 2199 | -170.4 ± 19.1 | | 75 |
| | 4 | 15 | 1586 | -118.2 ± 11.3 | | 88 |
| 6 | 8 | 20 | 2076 | -145.3 ± 29.5 | 9.1 ± 0.5 | 95 |
| | 12 | 18 | 1876 | -183.8 ± 23.0 | | 67 |

**Table S7.** Summary of thermoelectric voltage measurements for $Au^{TS}$/Fc@HKUST-1(2)//$Ga_2O_3$/EGaIn junctions.

| Cycle | $\Delta T$ (K) | $N_{junctions}$ | counts | $\Delta V_{mean} \pm \sigma_{\Delta V}$ | $S_{HKUST-1}$ (μV/K) | Yield (%) |
|---|---|---|---|---|---|---|
| 1 | 4 | 20 | 2094 | -45.6 ± 5.6 | 6.9 ± 0.0 | 91 |
| | 8 | 21 | 2211 | -69.5 ± 8.4 | | 88 |
| | 12 | 20 | 2118 | -92.9 ± 20.5 | | 83 |
| 2 | 4 | 27 | 2784 | -55.7 ± 12.9 | 9.1 ± 0.3 | 84 |
| | 8 | 29 | 2988 | -85.2 ± 20.8 | | 74 |
| | 12 | 29 | 3016 | -120.2 ± 15.2 | | 85 |
| 3 | 4 | 20 | 2045 | -58.6 ± 6.3 | 12.8 ± 0.7 | 83 |
| | 8 | 21 | 2219 | -102.8 ± 11.5 | | 84 |
| | 12 | 27 | 2984 | -156.6 ± 19.4 | | 84 |
| 4 | 4 | 24 | 2507 | -106.1 ± 15.9 | 12.9 ± 1.2 | 53 |
| | 8 | 23 | 2327 | -159.4 ± 13.7 | | 92 |
| | 12 | 29 | 2985 | -198.6 ± 23.5 | | 94 |
| 5 | 4 | 26 | 2696 | -105.6 ± 10.1 | 13.1 ± 0.4 | 90 |
| | 8 | 25 | 2672 | -156.6 ± 15.7 | | 89 |
| | 12 | 30 | 3172 | -201.4 ± 20.2 | | 94 |
| 6 | 4 | 18 | 1773 | -236.0 ± 17.9 | 13.5 ± 0.3 | 89 |
| | 8 | 20 | 2074 | -283.6 ± 12.3 | | 95 |
| | 12 | 20 | 2072 | -355.0 ± 12.2 | | 95 |

**Table S8.** Summary of thermoelectric voltage measurements for $Au^{TS}$/TCNQ@HKUST-1(2)//$Ga_2O_3$/EGaIn junctions.

| Cycle | $\Delta T$ (K) | $N_{junctions}$ | counts | $\Delta V_{mean} \pm \sigma_{\Delta V}$ | $S_{HKUST\text{-}1}$ (μV/K) | Yield (%) |
|---|---|---|---|---|---|---|
| 1 | 4 | 20 | 2091 | -53.8 ± 9.9 | 6.3 ± 0.3 | 95 |
| | 8 | 22 | 2262 | -76.5 ± 18.2 | | 96 |
| | 12 | 28 | 2690 | -94.7 ± 24.1 | | 70 |
| 2 | 4 | 20 | 2107 | - 49.4 ± 6.0 | 7.7 ± 0.6 | 100 |
| | 8 | 20 | 2118 | -72.9 ± 10.2 | | 91 |
| | 12 | 25 | 2590 | -104.9 ± 12.9 | | 83 |
| 3 | 4 | 23 | 2420 | -76.5 ± 12.4 | 9.9 ± 0.7 | 82 |
| | 8 | 29 | 3081 | -96.5 ± 11.7 | | 94 |
| | 12 | 28 | 2979 | -137.9 ± 19.7 | | 82 |
| 4 | 4 | 20 | 2043 | -69.1 ± 8.3 | 9.9 ± 0.2 | 100 |
| | 8 | 21 | 2129 | -103.7 ± 8.9 | | 95 |
| | 12 | 21 | 2243 | -141.1 ± 17.3 | | 100 |
| 5 | 4 | 23 | 2330 | -67.7 ± 8.5 | 10.5 ± 0.6 | 82 |
| | 8 | 20 | 2022 | -108.4 ± 13.3 | | 100 |
| | 12 | 21 | 2130 | -140.2 ± 25.0 | | 91 |
| 6 | 4 | 26 | 2693 | -69.1 ± 7.3 | 10.6 ± 0.2 | 100 |
| | 8 | 31 | 3227 | -108.9 ± 12.7 | | 89 |
| | 12 | 26 | 2611 | -145.9 ± 11.7 | | 74 |

**Table S9.** Summary of thermoelectric voltage measurements for $Au^{TS}$/HKUST-1(10)//$Ga_2O_3$/EGaIn junctions.

| Cycle | $\Delta T$ (K) | $N_{junctions}$ | counts | $\Delta V_{mean} \pm \sigma_{\Delta V}$ | $S_{HKUST-1}$ (μV/K) | Yield (%) |
|---|---|---|---|---|---|---|
| 1 | 4 | 27 | 2793 | -7.9 ± 5.6 | 2.5 ± 0.3 | 93 |
| | 8 | 20 | 2085 | -15.2 ± 3.0 | | 100 |
| | 12 | 24 | 2473 | -19.1 ± 7.7 | | 89 |
| 2 | 4 | 23 | 2142 | -8.6 ± 11.3 | 3.3 ± 0.2 | 96 |
| | 8 | 22 | 2268 | -16.3 ± 17.4 | | 88 |
| | 12 | 29 | 2970 | -26.7 ± 15.1 | | 100 |
| 3 | 4 | 15 | 1529 | -10.1 ± 7.8 | 3.1 ± 0.0 | 88 |
| | 8 | 15 | 1521 | -18.6 ± 7.7 | | 100 |
| | 12 | 19 | 1924 | -26.7 ± 11.4 | | 100 |
| 4 | 4 | 20 | 2022 | -20.3 ± 5.9 | 3.4 ± 0.1 | 95 |
| | 8 | 20 | 2090 | -29.5 ± 8.5 | | 83 |
| | 12 | 21 | 2140 | -39.5 ± 8.6 | | 95 |
| 5 | 4 | 20 | 2020 | -14.0 ± 9.0 | 4.1 ± 0.2 | 77 |
| | 8 | 20 | 2035 | -24.8 ± 15.0 | | 77 |
| | 12 | 21 | 2139 | -39.1 ± 13.6 | | 84 |
| 6 | 4 | 23 | 2349 | -14.3 ± 5.3 | 1.3 ± 0.2 | 96 |
| | 8 | 22 | 2334 | -28.5 ± 12.4 | | 85 |
| | 12 | 28 | 2926 | -39.5 ± 25.0 | | 82 |

**Table S10.** Summary of thermoelectric voltage measurements for $Au^{TS}/SC_nCOOH$($n$=2,10)//$Ga_2O_3$/EGaIn junctions.

| $n$ | $\Delta T$ (K) | $N_{junctions}$ | counts | $\Delta V_{mean} \pm \sigma_{\Delta V}$ | $S_{HKUST\text{-}1}$ (μV/K) | Yield (%) |
|---|---|---|---|---|---|---|
| | 4 | 17 | 1756 | -25.6 ± 4.1 | | 63 |
| 2 | 8 | 20 | 2063 | -44.7 ± 5.8 | 5.5 ± 0.3 | 77 |
| | 12 | 21 | 2175 | -60.3 ± 9.7 | | 72 |
| | 4 | 20 | 2066 | -24.9 ± 4.3 | | 95 |
| 10 | 8 | 20 | 2070 | -40.9 ± 10.3 | 4.9 ± 0.1 | 71 |
| | 12 | 15 | 1550 | -56.0 ± 9.8 | | 65 |

**3. References**

[1] L. G. Albano, D. H. de Camargo, G. R. Schleder, S. G. Deeke, T. P. Vello, L. D. Palermo, C. C. Corrêa, A. Fazzio, C. Wöll, C. C. Bufon, *Small* **2021**, *17*, 2101475

[2] L. G. Albano, T. P. Vello, D. H. de Camargo, R. M. da Silva, A. C. Padilha, A. Fazzio, C. C. Bufon, *Nano Lett.* **2020**, *20*, 1080

[3] K. Müller, N. Vankova, L. Schöttner, T. Heine, L. Heinke, *Chem. Sci.* **2019**, *10*, 153

[4] T. P. Vello, M. Strauss, C. A. R. Costa, C. C. Corrêa, C. C. B. Bufon, *Phys. Chem. Chem. Phys.* **2020**, *22*, 5839

[5] S. Park, J. W. Jo, J. Jang, T. Ohto, H. Tada, H. J. Yoon, *Nano Lett.* **2022**, *22*, 7682

[6] S. Park, J. Jang, Y. Tanaka, H. J. Yoon, *Nano Lett.* **2022**, *22*, 9693

[7] M. Kjærvik, P. M. Dietrich, A. Thissen, J. Radnik, A. Nefedov, C. Natzeck, C. Wöll, W. E. Unger, *J. Electron Spectrosc. Relat. Phenom.* **2021**, *247*, 147042
